# Design, synthesis and structure-activity relationship of 3,6-diaryl-7*H*-[1,2,4]triazolo[3,4-b][1,3,4]thiadiazines as novel tubulin inhibitors

**DOI:** 10.1038/s41598-017-10860-7

**Published:** 2017-09-20

**Authors:** Qile Xu, Kai Bao, Maolin Sun, Jingwen Xu, Yueting Wang, Haiqiu Tian, Daiying Zuo, Qi Guan, Yingliang Wu, Weige Zhang

**Affiliations:** 1 Key Laboratory of Structure-Based Drug Design and Discovery, Ministry of Education, Shenyang Pharmaceutical University, 103 Wenhua Road, Shenhe District Shenyang, 110016 China; 2 Department of Pharmacology, Shenyang Pharmaceutical University, 103 Wenhua Road, Shenhe District Shenyang, 110016 China

## Abstract

A novel series of 3,6-diaryl-7*H*-[1,2,4]triazolo[3,4-b][1,3,4]thiadiazines were designed, synthesized and biologically evaluated as vinylogous CA-4 analogues, which involved a rigid [1,2,4]triazolo[3,4-b][1,3,4]thiadiazine scaffold to fix the configuration of (*Z*,*E*)-butadiene linker of A-ring and B-ring. Among these rigidly vinylogous CA-4 analogues, compounds **4d**, **5b**, **5i**, **6c**, **6e**, **6g**, **6i** and **6k** showed excellent antiproliferative activities against SGC-7901, A549 and HT-1080 cell lines with IC_50_ values at the nanomolar level. Compound **6i** showed the most highly active antiproliferative activity against the three human cancer cell lines with an IC_50_ values of 0.011–0.015 µM, which are comparable to those of CA-4 (IC_50_ = 0.009–0.013 µM). Interestingly, SAR studies revealed that 3,4-methylenedioxyphenyl, 3,4-dimethoxyphenyl, 3-methoxyphenyl and 4-methoxyphenyl could replace the classic 3,4,5-trimethoxyphenyl in CA-4 structure and keep antiproliferative activity in this series of designed compounds. Tubulin polymerization experiments showed that **6i** could effectively inhibit tubulin polymerization, which was corresponded with CA-4, and immunostaining experiments suggested that **6i** significantly disrupted microtubule/tubulin dynamics. Furthermore, **6i** potently induced cell cycle arrest at G_2_/M phase in SGC-7901 cells. Competitive binding assays and docking studies suggested that compound **6i** binds to the tubulin perfectly at the colchicine binding site. Taken together, these results revealed that **6i** may become a promising lead compound for new anticancer drugs discovery.

## Introduction

Microtubules are composed of dynamic polymers of α- and β-tubulin subunits, which play an essential role in a variety of fundamental cell functions including the maintenance of cell shape, intracellular transport and cell division^1,2^. Due to the multiple functions of microtubules in cell mitosis, tubulin has become a highly attractive target for new anticancer drugs discovery^3,4^. Microtubule-targeting drugs normally can be grouped into microtubule stabilizing and microtubule destabilizing drugs that disrupt tubulin/microtubule dynamics by binding to the protein tubulin leading to cell death^5,6^. Microtubule-targeting drugs are known to interact with tubulin through many binding sites: the laulimalide, taxane/epothilone, taccolonolide, vinca alkaloid and colchicine sites. Paclitaxel, colchicine, and vinblastine represent the well-known three major types of tubulin binding agents which bind at three major sites on tubulin: the taxane, colchicine, and vinca alkaloid sites, respectively^7,8^. While clinically applied microtubule targeting drugs binding at the vinca alkaloid or taxanes sites in tubulin are greatly successful, there are no clinically applied colchicine-binding site anticancer drugs currently available^9^.

**Figure 1:**
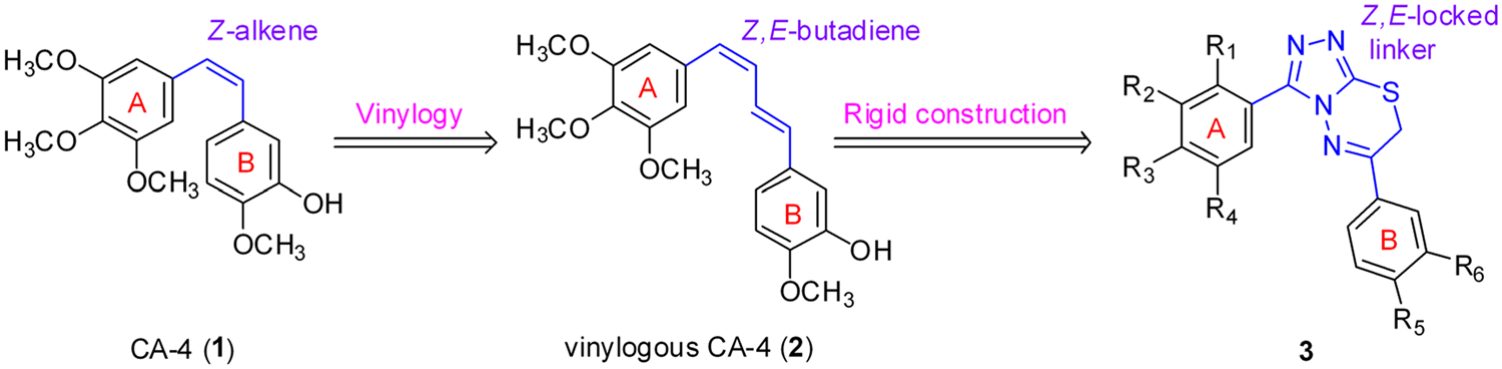
Structures of known tubulin inhibtors and design strategy for the target compounds.

Combretastatin A-4 (CA-4, 1, Fig. 1) is one of the most antiproliferative agents that bind at the colchicine binding site of tubulin^10^. Structure-activity relationship studies for CA-4 have shown that the *cis*-olefin bond (*Z*-alkene) and the presence of a 3,4,5-trimethoxyphenyl were essential for the activity^11^. Unfortunately, the *Z*-alkene of CA-4 tends to isomerize to the more stable but inactive *E*-alkene under the external conditions of light, heat, and protic media, resulting in a dramatic reduction in both antitubulin and antiproliferative activities^9,12^.

In the past several years, a wide variety of CA-4 analogues have been reported^13–25^. The modifications of CA-4 analogues can be generally classified into three major classes^23^: (I) introducing an extended side chain to the B ring; (II) replacing the alkene linker with another flexible linker such as a carbonyl, a methylene, or another suitable connection group; (III) replacing the alkene linker with a conformational rigid heterocyclic linker.

Accordingly, replacement of the *cis*-olefin bond by rigid heterocycle rings (such as five-membered and six-membered rings) that facilitate a *Z*-restricted configuration has proven to be one of the most effectively strategies in maintaining both antiproliferative and antitubulin activity^26–29^. The vinylogous CA-4 (**2**, Fig. 1) with a (*Z*,*E*)-butadiene linker has potent antiproliferative and tubulin polymerization inhibitory activity, but the main problem is that it tends to isomerize to the more stable but inactive (*E*,*E*)-isomeric derivative^30^. To the best of our knowledge, no report has attempted to lock the *Z,E*-configuration of vinylogous CA-4 with a rigid fused heterocycle to maintain biological activities.

The unique [1,2,4]triazolo[3,4-b][1,3,4]thiadiazine scaffold which was fused by triazole and thiadiazine represents an important kind of nitrogen heterocyclic with a broad spectrum of biological activities including anticancer activity^31,32^. As one of our research directions^33–37^, here we designed a series of 3,6-diaryl-7*H*-[1,2,4]triazolo[3,4-b][1,3,4]thiadiazines as vinylogous CA-4 analogues with general structure **3**, in which the (*Z*,*E*)-butadiene linker of vinylogous CA-4 was replaced with a novel rigid [1,2,4]triazolo[3,4-b][1,3,4]thiadiazine scaffold (Fig. 1). To explore the SAR of vinylogous CA-4 analogues, various substituents were introduced at different positions of the B-ring. Specifically we also have attempted to make modifications on the classical trimethoxy A-ring, although the 3,4,5-trimethoxyphenyl is a crucial fragment in CA-4, vinylogous CA-4 and their analogues^38^. Thirty three target compounds were synthesized and classified into the following three groups according to the A-ring substituents: (I) 2,3,4-trimethoxyphenyl (**4a**–**m**), (II) 3,4,5-trimethoxyphenyl (**5a**–**i**), (III) other A-ring substituent (**6a**–**k**). The representative compound **6i** was selected to investigate its mechanism of action. Besides, molecular modeling studies with the colchicine binding site of tubulin were performed with the most potent compound **6i**.

## Result and Discussion

### Chemistry

**Figure 2:**
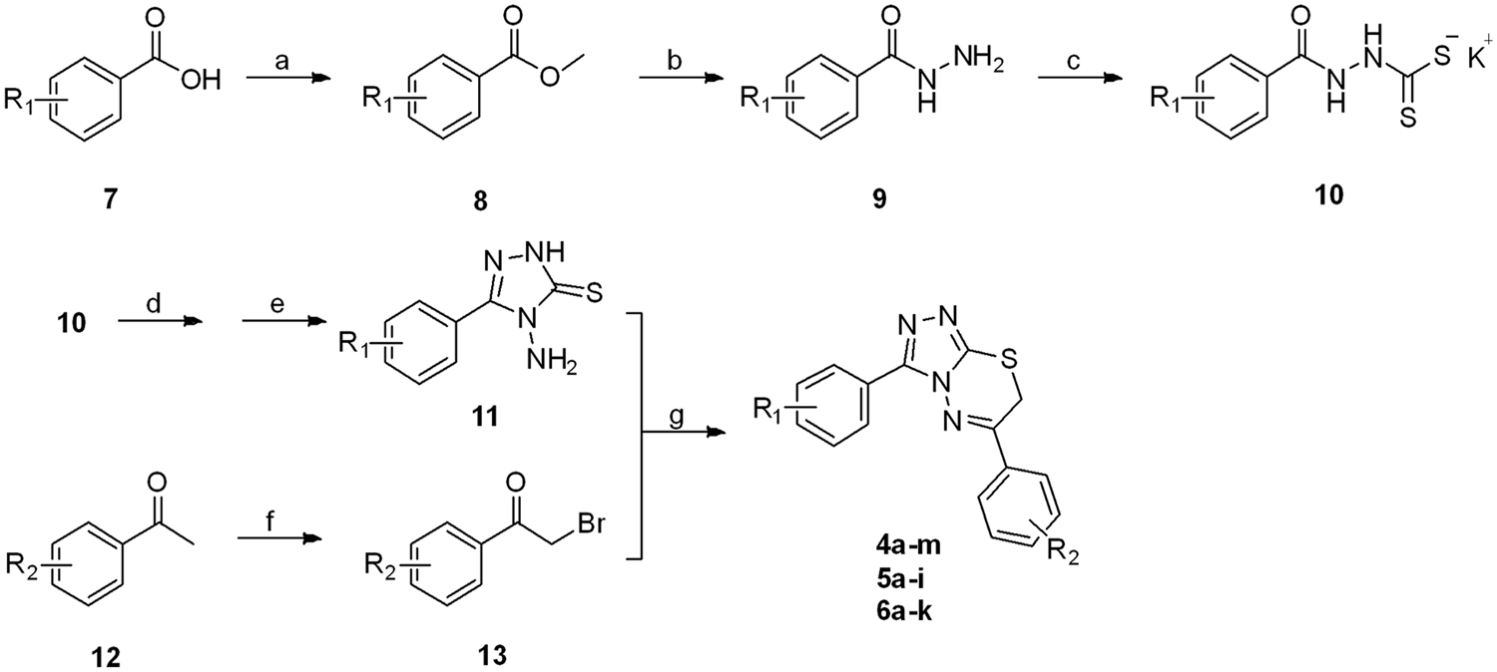
Reagents and conditions: (**a**) MeOH, conc. H_2_SO_4_, MW, 70 °C.; (**b**) N_2_H_4_·H_2_O, MeOH, reflux; (**c**) CS_2_, KOH, MeOH, 25 °C; (**d**) N_2_H_4_·H_2_O, H_2_O, MW, 100 °C.; (**e**) HCl, 0 °C; (f) CuBr_2_, CHCl_3_, EtOAc, reflux; (**g**) EtOH, MW, 80 °C.

The target compounds **4a**–**m**, **5a**–**i** and **6a**–**k** were prepared as outlined in Fig. 2. The substituted benzoic acids **7** were reacted with excess methanol with concentrated sulfuric acid as catalyst to afford the corresponding esters **8** which were further reacted with 80% hydrazine monohydrate in methanol to get hydrazides **9** under microwave (250 W, 70 ^o^C) irradiation. The hydrazides **9** were reacted with carbon disulphide and potassium hydroxide in methanol to give corresponding dithiocarbazinates **10** and then dithiocarbazinates **10** further reacted with excess 80% hydrazine monohydrate to get the key intermediate aryl triazoles **11**. On the other hand, the commercially available starting acetophenones **12** were subjected to α-bromination with copper bromide in refluxing chloroform/ethyl acetate to get α-bromoacetophenones **13**. Finally, α-bromoacetophenones **13** were reacted with aryl triazoles **11** to afford the target compounds in ethanol within 5 min under microwave (250 W, 80 ^o^C) irradiation in the absence of catalysts.

### *In vitro* anti-proliferative activity

*In vitro* antiproliferative activity against three human cancer cell lines, including gastric adenocarcinoma SGC-7901 cells, lung adenocarcinoma A549 cells and fibrosarcoma HT-1080 cells, was determined using a standard MTT assay with CA-4 as the positive control. As shown in Table 1, it is evident that most of these new compounds showed moderate to excellent antiproliferative activity, indicating that the utilization of the rigid [1,2,4]triazolo[3,4-b][1,3,4]thiadiazine scaffold to lock the (*Z*,*E*)-butadiene linker of vinylogous CA-4 is an effective strategy to maintain potent antiproliferative activity. When A-ring was a 2,3,4-trimethoxyphenyl, compounds **4a**–**m** displayed only modest antiproliferative activity. Interestingly, the 4-methyl-substituted (B-ring) compound **4d** displayed the most active antiproliferative activity (0.028–0.073 µM); however, compound **4j** (B-ring was a 3-amino-4-methoxyphenyl) and **4 l** (B-ring was a 3-hydroxy-4-methoxyphenyl) exhibited only moderate activity. Compared to the 2,3,4-trimethoxyphenyl-substituted **4a**–**m**, 3,4,5-trimethoxy substitution at A-ring tended to enhance the potency of corresponding compounds **5a**–**i**. Among compounds **5a**–**i**, 4-methyl-substituted (B-ring) **5b** displayed the most potent antiproliferative activity with IC_50_ values of 0.016–0.027 μM and compound **5i** also effectively inhibited the three cell lines growth with IC_50_

**Table 1:** Antiproliferative activities of the target compounds and CA-4.

							IC_50_^a^ (μM)		
Comp.	R_1_	R_2_	R_3_	R_4_	R_5_	R_6_	SGC-7901	A549	HT-1080
**4a**	OCH_3_	OCH_3_	OCH_3_	H	F	H	32.6	21.2	31.3
**4b**	OCH_3_	OCH_3_	OCH_3_	H	Cl	H	24.5	23.2	24.7
**4c**	OCH_3_	OCH_3_	OCH_3_	H	Br	H	5.55	7.37	3.77
**4d**	OCH_3_	OCH_3_	OCH_3_	H	CH_3_	H	**0.066**	**0.073**	**0.028**
**4e**	OCH_3_	OCH_3_	OCH_3_	H	CF_3_	H	2.42	85.4	10.5
**4f**	OCH_3_	OCH_3_	OCH_3_	H	OCH_3_	H	62.7	50.2	61.4
**4g**	OCH_3_	OCH_3_	OCH_3_	H	SCH_3_	H	8.02	12.5	2.7
**4h**	OCH_3_	OCH_3_	OCH_3_	H	OCH_3_	F	43.2	16.3	51.9
**4i**	OCH_3_	OCH_3_	OCH_3_	H	OCH_3_	NO_2_	13.2	16.5	15.9
**4j**	OCH_3_	OCH_3_	OCH_3_	H	OCH_3_	NH_2_	1.57	1.58	0.89
**4k**	OCH_3_	OCH_3_	OCH_3_	H	OCH_3_	OBn	28.9	8.9	28.3
**4l**	OCH_3_	OCH_3_	OCH_3_	H	OCH_3_	OH	16.3	7.73	0.63
**4m**	OCH_3_	OCH_3_	OCH_3_	H	F	F	73.2	78.1	81.3
**5a**	H	OCH_3_	OCH_3_	OCH_3_	Cl	H	1.37	0.27	0.88
**5b**	H	OCH_3_	OCH_3_	OCH_3_	CH_3_	H	**0.027**	**0.019**	**0.016**
**5c**	H	OCH_3_	OCH_3_	OCH_3_	CF_3_	H	1.17	7.72	0.75
**5d**	H	OCH_3_	OCH_3_	OCH_3_	SCH_3_	H	0.13	0.46	0.18
**5e**	H	OCH_3_	OCH_3_	OCH_3_	OCH_3_	F	4.28	3.21	1.31
**5f**	H	OCH_3_	OCH_3_	OCH_3_	OCH_3_	NO_2_	11.3	8.88	5.76
**5g**	H	OCH_3_	OCH_3_	OCH_3_	OCH_3_	NH_2_	1.25	1.08	0.18
**5h**	H	OCH_3_	OCH_3_	OCH_3_	OCH_3_	OBn	1.66	2.94	1.57
**5i**	H	OCH_3_	OCH_3_	OCH_3_	OCH_3_	OH	**0.026**	**0.071**	**0.047**
**6a**	H	OCH_2_O		H	OCH_3_	F	0.065	0.19	0.13
**6b**	H	OCH_2_O		H	OCH_3_	NO_2_	10.5	15.7	0.080
**6c**	H	OCH_2_O		H	OCH_3_	NH_2_	**0.018**	**0.026**	**0.021**
**6d**	H	OCH_2_O		H	OCH_3_	OBn	8.49	16.3	7.32
**6e**	H	OCH_2_O		H	OCH_3_	OH	0.037	0.079	0.035
**6f**	H	OCH_3_	OCH_3_	H	OCH_3_	NO_2_	5.04	3.68	3.32
**6g**	H	OCH_3_	OCH_3_	H	OCH_3_	NH_2_	0.023	0.032	0.018
**6h**	H	OCH_3_	H	H	OCH_3_	NO_2_	2.55	4.2	1.91
**6i**	H	OCH_3_	H	H	OCH_3_	NH_2_	**0.011**	**0.015**	**0.014**
**6j**	H	H	OCH_3_	H	OCH_3_	NO_2_	8.04	5.68	2.32
**6k**	H	H	OCH_3_	H	OCH_3_	NH_2_	**0.036**	**0.038**	**0.023**
**CA-4** ^b^	—	—	—	—	—	—	**0.011**	**0.009**	**0.013**

^a^IC_50_: 50% inhibitory concentration. Each experiment was carried out in triplicate. ^b^positive control.

values of 0.026–0.071 μM.

We further examined the cytotoxic effect of **6a**–**k** by replacement of trimethoxy substituents with other substituents (3,4-methylenedioxy, 3,4-dimethoxy, 3-methoxy, 4-methoxy) at A-ring along with various substituents at ring B. Surprisingly, compounds **6c**, **6e**, **6 g**, **6i** and **6k** displayed nanomolar IC_50_ values against all tested cells and compound **6i** with a 3-methoxyphenyl at A-ring showed the most potent antiproliferative activity with an IC_50_ of 0.011–0.015 µM (comparable to CA-4 with an IC_50_ of 0.009–0.013 µM). These results clearly indicated that the classic 3,4,5-trimethoxyphenyl in CA-4 structure could be replaced by 3,4-methylenedioxyphenyl, 3,4-dimethoxyphenyl, 3-methoxyphenyl and 4-methoxyphenyl without significant reduction of antiproliferative activity in this series of designed compounds. A comparison of vinylogous CA-4 (*Z,E*) with the current compound **6i** (Table S1) was included in the Supporting Information.

### Tubulin assembly

**Figure 3:**
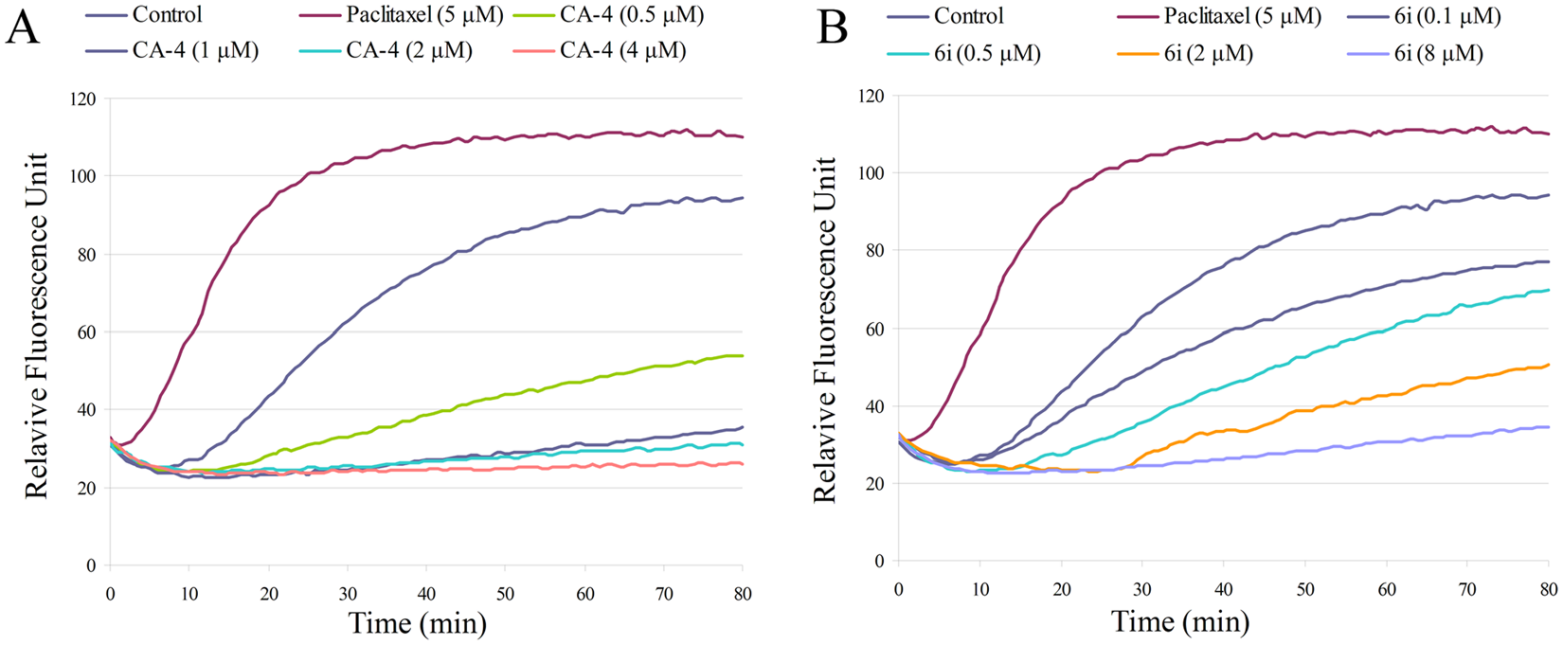
(**A**) CA-4 inhibits microtubule polymerization *in vitro*. (**B**) Compound **6i** inhibits microtubule polymerization *in vitro*. Tubulin was mixed with reaction buffer and incubated with CA-4 (0.5, 1, 2, 4 µM), paclitaxel (5.0 µM), **6i** (0.1, 0.5, 2.0, 8.0 µM) or vehicle DMSO (control). The reaction was monitored at 37 ^o^C.

To investigate whether the antiproliferative activity was produced by interaction between the target compounds and tubulin, the most active compound **6i** was investigated for its inhibition of tubulin polymerisation. This assay uses highly purified tubulin from porcine brain. CA-4 (**1**) and paclitaxel were used as positive and negative controls, respectively (Fig. 3A). As shown in Figs 3B, **6i** effectively inhibited tubulin polymerization with an IC_50_ value of 1.6 µM, which was slightly higher to that of CA-4 (IC_50 = _0.92 μM). The representative raw data for the polymerization assay of compound **6i** and CA-4 showed that both of them caused a dose-dependent inhibition of tubulin polymerization; in contrast, paclitaxel, a microtubule stabilizing agent, could distinctly promoted this process (Fig. 3). Thus, the results indicated that **6i** was a tubulin inhibitor.

### Immunofluorenscence studies

**Figure 4:**
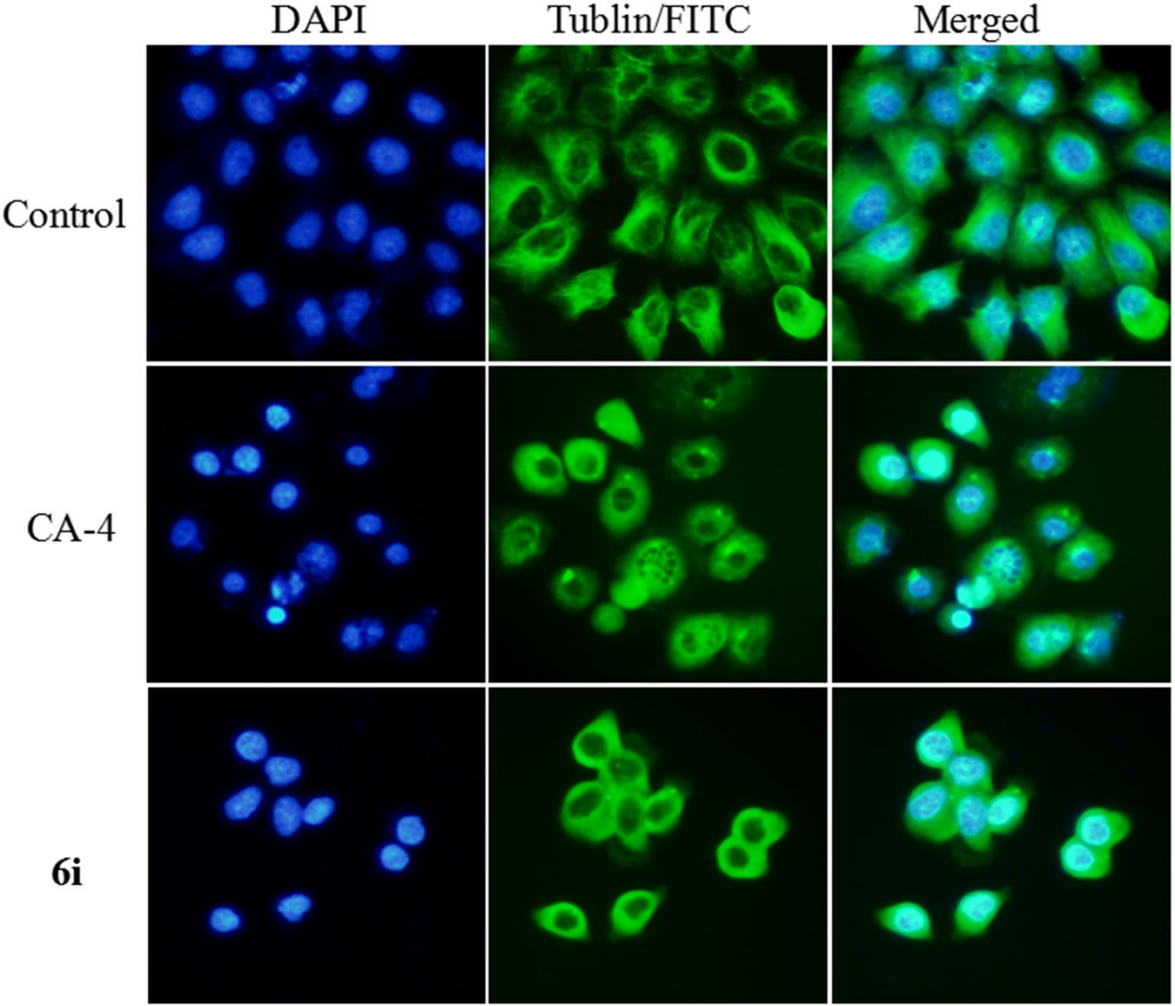
Effects of **6i** on tubulin network of SGC-7901 cells by immunofluorenscence. SGC-7901 cells were treated with DMSO (vehicle control), CA-4 (0.022 µM) or **6i** (0.022 µM) for 12 h. The left and middle panels represent the tubulin assembly stained with DAPI and FITC and the right panel represents a merge of the corresponding left and middle panels.

To directly test **6i** can target microtubule/tubulin, we treated SGC-7901 cells with 2-fold IC_50_ of **6i** and analyzed microtubules by immunocytochemistry staining. CA-4 was employed as positive control in this experiment. As illustrated in Fig. 4, the control cells displayed well-organized microtubule network throughout the cells. After treatment with **6i** and CA-4 (at their respective 2-fold IC_50_ concentrations, respectively), microtubules became irregular arrangement and organization, and the tubulin network showed a disruption. These results further confirmed that the target of **6i** was tubulin.

### Cell cycle analysis

**Figure 5:**
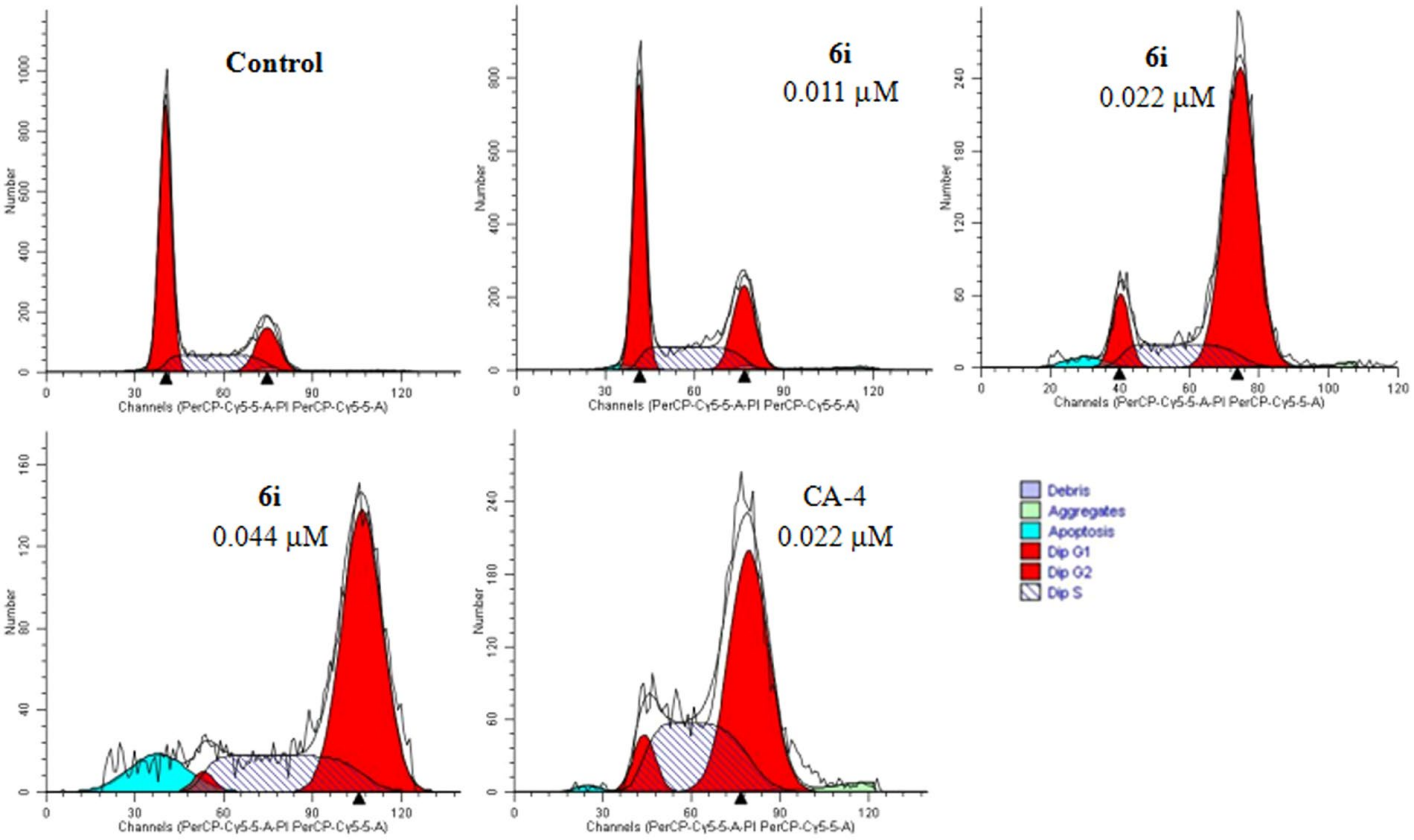
Compound **6i** affects the cell cycle progression in SGC-7901 cells. SGC-7901 cells were treated with compound **6i** (0.011, 0.022 and 0.044 µM, respectively) or CA-4 (0.022 µM) for 12 h.

It is well known that tubulin inhibitors such as colchicine and CA-4 arrest cell cycle distribution in G_2_/M phase. Thus, the effect of compound **6i** on the cell cycle progression of the SGC-7901 cells was investigated by flow cytometry analysis (Fig. 5). The SGC-7901 cells were treated with compound **6i** (1-, 2-, 4-fold IC_50_) for 12 h, and CA-4 (2-fold IC_50_) was used as a positive control. Flow cytometry analysis showed that both **6i** and CA-4 caused a potent cell cycle arrests in G_2_/M phase. In comparison, the untreated cells (control) showed normal distribution with more cell population in the G_1_ phase. Compound **6i** could arrest cell cycle distribution at the G_2_/M phase in a dose-dependent manner. Moreover, compound **6i** was found to cause subsequent apoptosis after G_2_/M arrest in SGC-7901 cells (Figure S1).

### Competitive tubulin-binding assay

**Figure 6:**
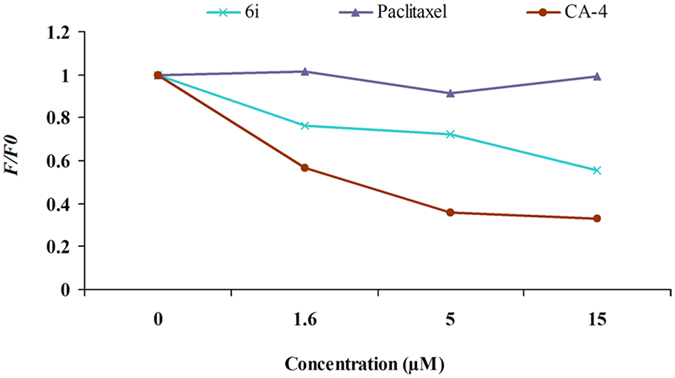
Competitive binding of **6i** to colchicine-binding site on tubulin. F/F_0_ represents inhibition rate (IR = F/F_0_) whereas F_0_ refers to fluorescence of the 5.0 µM colchicine-tubulin complex, and F describes the fluorescence of a given concentration (1.6 µM, 5.0 µM, and 15.0 µM) of CA-4, compounds **6i** and paclitaxel competition with the 5.0 µM colchicine-tubulin complex.

To confirm whether these designed compounds could bind to the colchicine site on tubulin, compound **6i** was investigated for its ability of competitive inhibition of colchicine binding^39,40^. CA-4 and paclitaxel were used as a positive and a negative control, respectively. The intrinsic fluorescence of colchicine increases upon binding to the tubulin, which could be used as an index for **6i** or CA-4 competition with colchicine in tubulin binding. As shown in Fig. 6, the fluorescence of a colchicine-tubulin complex was reduced in the presence of CA-4 or **6i** in a dose-dependent manner. These observations indicated that they inhibit the binding of colchicine to the tubulin, thereby suggesting the direct binding of compound **6i** at the colchicine binding site of tubulin.

### Molecular docking studies

**Figure 7:**
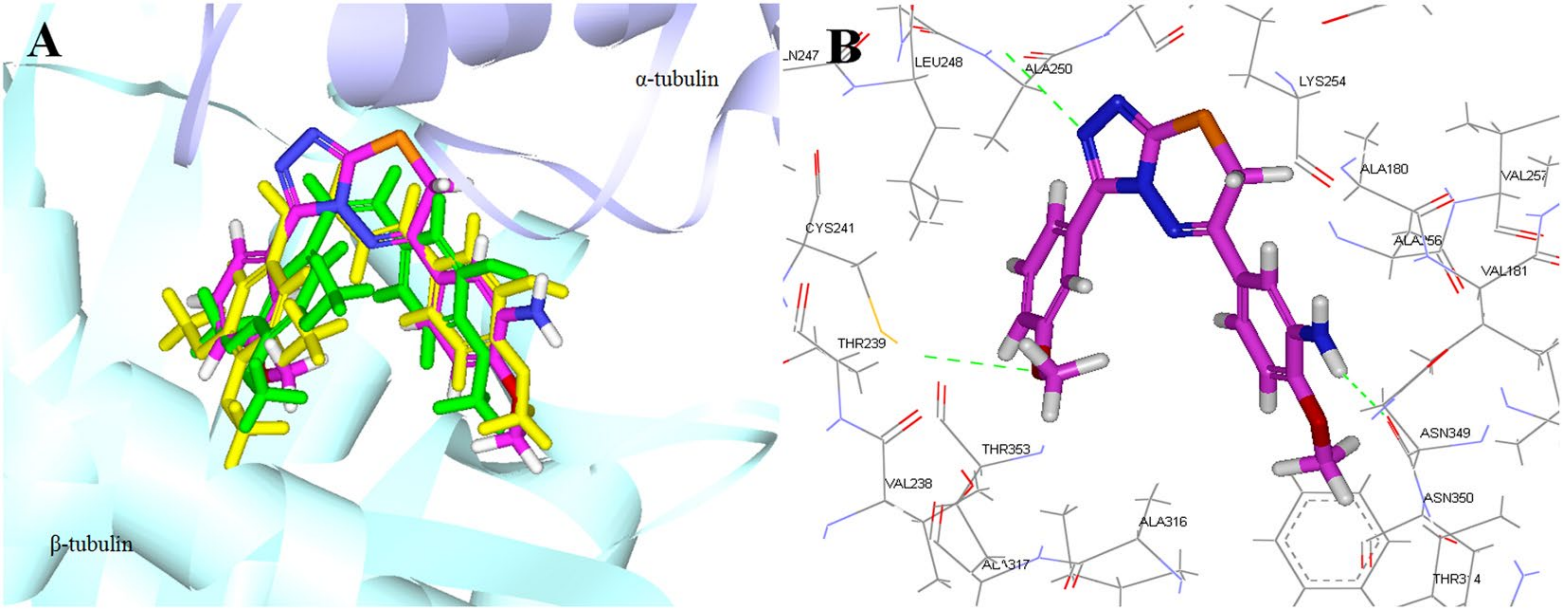
(**A**) Overlay of **6i** (pink), CA-4 (green) and vinylogous CA-4 (yellow) in the colchicine binding site. (**B**) The hydrogen bond interactions are displayed as green dotted lines and the amino acids are displayed as stick models.

Molecular docking studies were performed by using the CDOCKER program in Discovery Studio 3.0 software to explore the binding ability of **6i** to the colchicine binding site of tubulin (PDB: 1SA0). Docking studies revealed that the compound **6i** coincides closely with CA-4 and vinylogous CA-4, and they occupied the colchicine binding site of α,β-tubulin mostly buried in the β subunit (Fig. 7A). For compound **6i**, a hydrogen bond formed between the oxygen atom of methoxyl group and the thiol group of Cysβ241 (note: In some publications this residue is numbered as Cysβ239)^9^. The nitrogen atom of the amino group on the B-ring formed another hydrogen bond with the residue of Asnβ349. Additionally, the nitrogen atom on triazolothiadiazine linker of compound **6i** formed a direct hydrogen bond with the residue of Alaβ250 (Fig. 7B). The results of docking studies suggested that compound **6i** binds to the tubulin possibly at the colchicine binding site on tubulin, albeit with lesser affinity than CA-4.

In summary, we designed and synthesized a novel series of 3,6-diaryl-7*H*-[1,2,4]triazolo[3,4-b][1,3,4]thiadiazines as analogues of vinylogous CA-4. Among them, compounds **4d**, **5b**, **5i**, **6c**, **6e**, **6g**, **6i** and **6k** showed excellent antiproliferative activities against SGC-7901, A549 and HT-1080 cell lines with IC_50_ values at nanomolar level. Compound **6i** with a 3-methoxyphenyl in place of the classic trimethoxyphenyl A-ring, showed the most potent antiproliferative activity with an IC_50_ of 0.011–0.015 µM, which was comparable to that of CA-4 (0.009–0.013 µM). The tubulin polymerization and immunofluorescence experiments showed that **6i** effectively inhibited tubulin polymerization and resulted in a disruption on the tubulin network in SGC-7901 cells. Furthermore, **6i** caused a potent cell cycle arrest in G_2_/M phase in a dose-dependent manner. Competitive binding assay and molecular docking studies indicated that compound **6i** binds to the colchicine binding site of tubulin.

Taken together, a novel rigid [1,2,4]triazolo[3,4-b][1,3,4]thiadiazine scaffold was applied to lock the (*Z,E*)-butadiene linker of A-ring and B-ring of vinylogous CA-4 and proved to be a successful strategy in retaining both antiproliferative and antitubulin activities. For the new skeleton, it is worth noting that 3,4-methylenedioxyphenyl, 3,4-dimethoxyphenyl, 3-methoxyphenyl and 4-methoxyphenyl could replace the crucially classic 3,4,5-trimethoxyphenyl in CA-4 and vinylogous CA-4. The present study not only resulted in a series of excellent tubulin inhibitors, but transcended the common understanding of structure-activities relationships of CA-4, vinylogous CA-4 and their analogues. Further modifications of these novel CBSIs and detailed mechanistic studies for the anticancer properties are underway in our lab.

## Methods

### Reagents and equipment

All solvents and chemical reagent were got from commercially available sources and were used without purification. The microwave reactions were performed on a discover-sp single mode microwave reactor from CEM Corporation. The progress of reactions was monitored by TLC using silica gel plates under UV light. NMR spectra were recorded on a Bruker AVANCE 400, or 600 spectrometer (^1^H, 400 MHz, 600 MHz;^13^C, 100 MHz, 150 MHz), in CDCl_3_ or DMSO-*d*_6_ (TMS as internal standard). Chemical shifts are expressed as parts per million downfield from tetramethylsilane. Mass spectra (MS) were measured on an Agilent 1100-sl mass spectrometer with an electrospray ionisation source. Melting points were measured on a hot stage microscope (X-4, Beijing Taike Ltd.) and are uncorrected.

### General synthetic procedures for target compounds

To a solution of appropriately intermediates **11** (10 mmol) in absolute ethyl alcohol (15 mL), was added the appropriate α-bromoacetophenones **13** (10 mmol). The mixture was heated under microwave irradiation at 80 °C for 30 min. After the reaction completed, water was then added to the mixture and the precipitate formed was collected and crystallized from the proper solvent. Reduction of the nitro groups of **4i**, **5f**, **6b**, **6f**, **6h** and **6j** in a mixture of hydrazine hydrate, ferric chloride hexahydrate and activated carbon in methanol provided the corresponding **4j**, **5g**, **6c**, **6g**, **6i** and **6k**, respectively^33^. Debenzylation of compounds **4k**, **5h** and **6d** with titanium tetrachloride afforded, respectively the phenol derivatives, **4l**, **5i** and **6e** ^33^.

### 3-(2,3,4-Trimethoxyphenyl)-6-(4-fluorophenyl)-7H-[1,2,4]triazolo[3,4-b][1,3,4]thiadiazine (4a)

White solid; yield: 65%; M.p.: 92–94 ^o^C; ^1^H-NMR (400 MHz, CDCl_3_): δ 7.81–7.85 (m, 2 H), 7.32 (d, *J* = 8.6 Hz, 1 H), 7.11–7.15 (m, 2 H), 6.78 (d, *J* = 8.6 Hz, 1 H), 3.98 (s, 2 H), 3.93 (s, 3 H), 3.87 (s, 3 H), 3.77 (s, 3 H); ^13^C-NMR (100 MHz, CDCl_3_): δ 166.1, 163.6, 156.0, 152.7, 151.8, 142.0, 141.1, 129.7, 126.5 (d, *J* = 8.8 Hz, 2 C), 126.2, 116.2 (d, *J* = 21.8 Hz, 2 C), 112.9, 107.1, 61.6, 60.9, 56.1, 23.5; ESI-MS: m/z = 401.3 [M + H]^+^, 423.2 [M + Na]^+^.

### 3-(2,3,4-Trimethoxyphenyl)-6-(4-chlorophenyl)-7H-[1,2,4]triazolo[3,4-b][1,3,4]thiadiazine (4b)

White solid; yield: 82%; M.p.: 88–90 ^o^C; ^1^H-NMR (400 MHz, CDCl_3_): δ 7.75 (d, *J* = 8.6 Hz, 2 H), 7.39 (d, *J* = 8.6 Hz, 2 H), 7.29 (d, *J* = 8.7 Hz, 1 H), 6.77 (d, *J* = 8.7 Hz, 1 H), 3.98 (s, 2 H), 3.92 (s, 3 H), 3.86 (s, 3 H), 3.74 (s, 3 H); ^13^C-NMR (100 MHz, CDCl_3_): δ 155.9, 152.6, 151.7, 151.7, 141.9, 141.0, 138.0, 131.9, 129.2 (2 C), 128.5 (2 C), 126.1, 112.8, 107.0, 61.5, 60.8, 56.0, 23.3; ESI-MS: m/z = 417.3 [M + H]^+^, 439.2 [M + Na]^+^.

### 3-(2,3,4-Trimethoxyphenyl)-6-(4-bromophenyl)-7H-[1,2,4]triazolo[3,4-b][1,3,4]thiadiazines (4c)

White solid; yield: 63%; M.p.: 96–98 ^o^C; ^1^H-NMR (400 MHz, CDCl_3_): δ 7.68 (d, *J* = 8.6 Hz, 2 H), 7.56 (d, *J* = 8.6 Hz, 2 H), 7.30 (d, *J* = 8.7 Hz, 1 H), 6.78 (d, *J* = 8.7 Hz, 1 H), 3.98 (s, 2 H), 3.92 (s, 3 H), 3.86 (s, 3 H), 3.75 (s, 3 H); ^13^C-NMR (100 MHz, CDCl_3_): δ 156.0, 152.7, 151.9 (2 C), 142.0, 141.1, 132.4, 132.3 (2 C), 128.7 (2 C), 126.6, 126.2, 112.8, 107.1, 61.6, 60.9, 56.1, 23.3; ESI-MS: m/z = 460.9 [M + H]^+^.

### 3-(2,3,4-Trimethoxyphenyl)-6-(4-methyphenyl)-7H-[1,2,4]triazolo[3,4-b][1,3,4]thiadiazine (4d)

Yellow solid; yield: 79%; M.p.: 89–92 ^o^C; ^1^H-NMR (400 MHz, CDCl_3_): δ 7.71 (d, *J* = 8.2 Hz, 2 H), 7.33 (d, *J* = 8.6 Hz, 1 H), 7.24 (d, *J* = 8.2 Hz, 2 H), 6.78 (d, *J* = 8.6 Hz, 1 H), 3.97 (s, 2 H), 3.93 (s, 3 H), 3.87 (s, 3 H), 3.76 (s, 3 H), 2.39 (s, 3 H); ^13^C-NMR (100 MHz, CDCl_3_): δ 155.9, 152.8, 152.7, 151.7, 142.5, 142.0, 141.3, 130.7, 129.7 (2 C), 127.2 (2 C), 126.3, 113.0, 107.0, 61.6, 60.9, 56.1, 23.4, 21.5; ESI-MS: m/z = 397.3 [M + H]^+^, 419.3 [M + Na]^+^.

### 3-(2,3,4-Trimethoxyphenyl)-6-(4-(trifluoromethyl)phenyl)-7H-[1,2,4]triazolo[3,4-b][1,3,4]thiadiazine (4e)

Yellow solid; yield: 67%; M.p.: 95–96 ^o^C; ^1^H-NMR (400 MHz, CDCl_3_): δ 7.94 (d, *J* = 8.3 Hz, 2 H), 7.68 (d, *J* = 8.3 Hz, 2 H), 7.30 (d, *J* = 8.6 Hz, 1 H), 6.78 (d, *J* = 8.6 Hz, 1 H), 4.04 (s, 2 H), 3.92 (s, 3 H), 3.86 (s, 3 H), 3.74 (s, 3 H); ^13^C-NMR (100 MHz, CDCl_3_): δ 156.1, 152.6, 152.0, 151.5, 142.0, 141.1, 137.0, 133.2 (d, *J* = 29.8 Hz), 127.7 (2 C), 126.2, 126.0, 124.9, 122.2, 112.8, 107.1, 61.6, 60.9, 56.1, 23.5; ESI-MS: m/z = 451.0 [M + H]^+^.

### 3-(2,3,4-Trimethoxyphenyl)-6-(4-methoxyphenyl)-7H-[1,2,4]triazolo[3,4-b][1,3,4]thiadiazine (4 f)

Yellow solid; yield: 80%; M.p.: 92–95 ^o^C; ^1^H-NMR (400 MHz, CDCl_3_): δ 7.76 (d, *J* = 8.8 Hz, 2 H), 7.29 (d, *J* = 8.6 Hz, 1 H), 6.90 (d, *J* = 8.8 Hz, 2 H), 6.75 (d, *J* = 8.6 Hz, 1 H), 3.95 (s, 2 H), 3.90 (s, 3 H), 3.84 (s, 3 H), 3.81 (s, 3 H), 3.74 (s, 3 H); ^13^C-NMR (100 MHz, CDCl_3_): δ 162.5, 155.9, 152.7, 152.5, 151.6, 142.0, 141.3, 129.0 (2 C), 126.2, 125.7, 114.4 (2 C), 113.1, 107.0, 61.6, 60.9, 56.1, 55.5, 23.3; ESI-MS: m/z = 413.3 [M + H]^+^, 432.3 [M + Na]^+^.

### 3-(2,3,4-Trimethoxyphenyl)-6-(4-methylthiophenyl)-7H-[1,2,4]triazolo[3,4-b][1,3,4]thiadiazine (4g)

Red solid; yield: 75%; M.p.: 86–88 ^o^C; ^1^H-NMR (400 MHz, CDCl_3_): δ 7.72 (d, *J* = 8.5 Hz, 2 H), 7.31 (d, *J* = 8.6 Hz, 1 H), 7.23 (d, *J* = 8.5 Hz, 2 H), 6.77(d, *J* = 8.6 Hz, 1 H), 3.96 (s, 2 H), 3.92 (s, 3 H), 3.86 (s, 3 H), 3.75 (s, 3 H), 2.49 (s, 3 H); ^13^C-NMR (100 MHz, CDCl_3_): δ 155.9, 152.7, 152.3, 151.7, 144.4, 142.0, 129.5, 127.5 (3 C), 126.2, 125.6 (2 C), 113.0, 107.0, 61.6, 60.9, 56.1, 23.2, 14.9; ESI-MS: m/z = 429.0 [M + H]^+^.

### 3-(2,3,4-Trimethoxyphenyl)-6-(3-fluoro-4-methoxyphenyl)-7H-[1,2,4]triazolo[3,4-b][1,3,4]thiadiazine (4h)

Yellow solid; yield: 71%; M.p.: 92–94 ^o^C; ^1^H-NMR (400 MHz, CDCl_3_): δ 7.62 (d, *J* = 12.2 Hz, 1 H), 7.54 (d, *J* = 8.3 Hz, 1 H), 7.32 (d, *J* = 8.6 Hz, 1 H), 6.97–7.01 (m, 1 H), 6.79 (d, *J* = 8.6 Hz, 1 H), 3.93 (s, 8 H), 3.88 (s, 3 H), 3.76 (s, 3 H); ^13^C-NMR (100 MHz, CDCl_3_): δ 155.0, 152.6, 151.7, 150.7, 150.3, 150.1, 149.9, 149.8, 141.0, 140.1, 125.2, 123.1 (d, *J* = 3.0 Hz), 113.7 (d, *J* = 20.0 Hz), 111.9, 106.1, 60.6, 59.9, 55.3, 55.1, 22.1; ESI-MS: m/z = 431.3 [M + H]^+^, 453.3 [M + Na]^+^.

### 3-(2,3,4-Trimethoxyphenyl)-6-(3-nitro-4-methoxyphenyl)-7H-[1,2,4]triazolo[3,4-b][1,3,4]thiadiazine (4i)

Yellow solid; yield: 79%; M.p.: 134–136 ^o^C; ^1^H-NMR (600 MHz, DMSO-*d*_6_): δ 8.39 (d, *J* = 2.4 Hz, 1 H), 8.16 (dd, *J* = 8.9 Hz, *J* = 2.4 Hz, 1 H), 7.52 (d, *J* = 8.9 Hz, 1 H), 7.24 (d, *J* = 8.7 Hz, 1 H), 6.96 (d, *J* = 8.7 Hz, 1 H), 4.45 (s, 2 H), 3.99 (s, 3 H), 3.88 (s, 3 H), 3.79(s, 3 H), 3.70 (s, 3 H); ^13^C-NMR (150 MHz, DMSO-*d*_6_): δ 156.0, 154.7, 153.3, 152.5, 151.0, 141.9, 141.6, 139.6, 133.5, 126.4, 125.9, 124.5, 115.9, 112.9, 108.1, 61.8, 60.8, 57.6, 56.4, 23.1; ESI-MS: m/z = 458.3 [M + H]^+^, 480.2 [M + Na]^+^.

### 3-(2,3,4-Trimethoxyphenyl)-6-(3-amino-4-methoxyphenyl)-7H-[1,2,4]triazolo[3,4-b][1,3,4]thiadiazine (4j)

Yellow solid; yield: 74%; M.p.: 93–94 ^o^C; ^1^H-NMR (400 MHz, CDCl_3_): δ 7.30 (d, *J* = 8.7 Hz, 1 H), 7.22 (d, *J* = 2.0 Hz, 1 H), 7.14 (dd, *J* = 8.7 Hz, *J* = 2.0 Hz, 1 H), 6.76 (d, *J* = 8.6 Hz, 2 H), 3.91 (s, 5 H), 3.87 (s, 3 H), 3.85 (s, 3 H), 3.76(s, 3 H); ^13^C-NMR (100 MHz, CDCl_3_): δ 155.8, 153.0, 152.7, 151.6, 150.3, 142.0, 136.9, 129.8, 126.2, 126.0, 118.5, 113.3, 112.6, 109.8, 107.0, 61.6, 60.9, 56.1, 55.7, 23.3; ESI-MS: m/z = 428.3 [M + H]^+^, 450.3 [M + Na]^+^.

### 3-(2,3,4-Trimethoxyphenyl)-6-(3-benzyloxy-4-methoxyphenyl)-7H-[1,2,4]triazolo[3,4-b][1,3,4]thiadiazine (4k)

3Gray solid; yield: 82%; M.p.: 85–86 ^o^C; ^1^H-NMR (400 MHz, CDCl_3_): δ 7.45 (d, *J* = 1.8 Hz, 1 H), 7.32–7.36 (m, 4 H), 7.26–7.28 (m, 3 H), 6.90 (d, *J* = 8.6 Hz, 1 H), 6.80 (d, *J* = 8.6 Hz, 1 H), 5.10 (s, 2 H), 3.93 (s, 6 H), 3.89 (s, 2 H), 3.86 (s, 3 H), 3.71 (s, 3 H); ^13^C-NMR (100 MHz, CDCl_3_): δ 154.9, 152.0, 151.7, 151.2, 150.6, 147.3, 141.0, 140.3, 135.3, 127.6 (2 C), 127.1, 126.4 (2 C), 125.2, 124.9, 120.5, 112.2, 111.2, 110.0, 105.9, 70.1, 60.5, 59.8, 55.1 (2 C), 22.2; ESI-MS: m/z = 519.2 [M + H]^+^, 541.1 [M + Na]^+^.

### 3-(2,3,4-Trimethoxyphenyl)-6-(3-hydroxy-4-methoxyphenyl)-7H-[1,2,4]triazolo[3,4-b][1,3,4]thiadiazine (4l)

Brown solid; yield: 83%; M.p.: 88–89 ^o^C; ^1^H-NMR (400 MHz, DMSO-*d*_6_): δ 9.48 (s, 1 H), 7.39 (dd, *J* = 8.6 Hz, *J* = 2.3 Hz, 1 H), 7.33 (d, *J* = 2.3 Hz, 1 H), 7.21 (d, *J* = 8.6 Hz, 1 H), 7.03 (d, *J* = 8.6 Hz, 1 H), 6.96 (d, *J* = 8.6 Hz, 1 H), 4.35 (s, 2 H), 3.88 (s, 3 H), 3.82 (s, 3 H), 3.78 (s, 3 H), 3.72 (s, 3 H); ^13^C-NMR (100 MHz, DMSO-*d*_6_): δ 155.9, 155.0, 152.6, 151.7, 151.0, 147.2, 142.0, 142.0, 126.5, 126.0, 120.7, 113.6, 113.3, 112.1, 108.0, 61.9, 60.9, 56.5, 56.1, 23.1; ESI-MS: m/z = 429.1 [M + H]^+^, 451.1 [M + Na]^+^.

### 3-(2,3,4-Trimethoxyphenyl)-6-(3,4-difluorophenyl)-7H-[1,2,4]triazolo[3,4-b][1,3,4]thiadiazine (4m)

Pale-yellow solid; yield: 66%; M.p.: 95–97 ^o^C; ^1^H-NMR (400 MHz, CDCl_3_): δ 7.68–7.72 (m, 1 H), 7.54–7.56 (m, 1 H), 7.31 (d, *J* = 8.6 Hz, 1 H), 7.22–7.26 (m, 1 H), 6.79 (d, *J* = 8.6 Hz, 1 H), 3.96 (s, 2 H), 3.94 (s, 3 H), 3.89 (s, 3 H), 3.76 (s, 3 H); ^13^C-NMR (100 MHz, CDCl_3_): δ 156.0, 152.6, 151.8, 150.5, 142.0, 130.6, 126.1 (2 C), 123.8, 117.9, 117.8, 116.5, 116.4, 112.8, 107.1, 61.5, 60.8, 56.0, 23.2; ESI-MS: m/z = 419.3 [M + H]^+^, 441.2 [M + Na]^+^.

### 3-(3,4,5-Trimethoxyphenyl)-6-(4-chlorophenyl)-7H-[1,2,4]triazolo[3,4-b][1,3,4]thiadiazine (5a)

Pale-yellow solid; yield: 62%; M.p.: 125–127 ^o^C; ^1^H-NMR (400 MHz, CDCl_3_): δ 7.88 (d, *J* = 8.6 Hz, 2 H), 7.49 (d, *J* = 8.6 Hz, 2 H), 7.42 (s, 2 H), 4.01 (s, 2 H), 3.91 (s, 3 H), 3.88(s, 6 H); ^13^C-NMR (100 MHz, CDCl_3_): δ 153.2 (2 C), 152.5, 152.1, 141.4, 139.9, 138.5, 131.9, 129.5 (2 C), 128.3 (2 C), 121.1, 105.4 (2 C), 60.9, 56.2 (2 C), 22.9; ESI-MS: m/z = 417.3 [M + H]^+^.

### 3-(3,4,5-Trimethoxyphenyl)-6-(4-methyphenyl)-7H-[1,2,4]triazolo[3,4-b][1,3,4]thiadiazine (5b)

Brown solid; yield: 70%; M.p.: 183–185 ^o^C; ^1^H-NMR (600 MHz, DMSO-*d*_6_): δ 7.96 (d, *J* = 8.0 Hz, 2 H), 7.41 (s, 2 H), 7.39 (d, *J* = 8.0 Hz, 2 H), 4.42 (s, 2 H), 3.84 (s, 6 H), 3.75 (s, 3 H), 2.39 (s, 3 H); ^13^C-NMR (150 MHz, DMSO-*d*_6_): δ 156.0, 153.3 (2 C), 151.4, 142.7, 142.7, 139.5, 130.9, 130.1 (2 C), 127.7 (2 C), 121.7, 105.6 (2 C), 60.6, 56.3 (2 C), 22.8, 21.4; ESI-MS: m/z = 397.3 [M + H]^+^, 419.3 [M + Na]^+^.

### 3-(3,4,5-Trimethoxyphenyl)-6-(4-(trifluoromethyl)phenyl)-7H-[1,2,4]triazolo[3,4-b][1,3,4]thiadiazine (5c)

Yellow solid; yield: 77%; M.p.: 105–106 ^o^C; ^1^H-NMR (400 MHz, CDCl_3_): δ 8.06 (d, *J* = 8.2 Hz, 2 H), 7.76 (d, *J* = 8.2 Hz, 2 H), 7.37 (s, 2 H), 4.09 (s, 2 H), 3.89 (s, 3 H), 3.85 (s, 6 H); ^13^C-NMR (100 MHz, CDCl_3_): δ 153.2 (2 C), 152.5, 152.3, 141.6, 140.0, 136.9, 133.6 (d, *J* = 32.6 Hz), 127.6 (2 C), 126.2 (2 C), 124.4, 122.6, 120.9, 105.5 (2 C), 61.0, 56.2 (2 C), 23.1; ESI-MS: m/z = 451.1 [M + H]^+^, 473.1 [M + Na]^+^.

### 3-(3,4,5-Trimethoxyphenyl)-6-(4-methylthiophenyl)-7H-[1,2,4]triazolo[3,4-b][1,3,4]thiadiazine (5d)

Gray solid; yield: 79%; M.p.: 92–94 ^o^C; ^1^H-NMR (400 MHz, CDCl_3_): δ 7.84 (d, *J* = 8.6 Hz, 2 H), 7.45 (s, 2 H), 7.30 (d, *J* = 8.6 Hz, 2 H), 3.99 (s, 2 H), 3.90 (s, 3 H), 3.88(s, 6 H), 2.53 (s, 3 H); ^13^C-NMR (150 MHz, CDCl_3_): δ 153.1 (2 C), 153.1, 145.0, 139.8, 129.4, 127.2 (3 C), 125.6 (4 C), 105.4 (2 C), 60.9, 56.2 (2 C), 22.8, 14.8; ESI-MS: m/z = 429.1 [M + H]^+^, 451.1 [M + Na]^+^.

### 3-(3,4,5-Trimethoxyphenyl)-6-(3-fluoro-4-methoxyphenyl)-7H-[1,2,4]triazolo[3,4-b][1,3,4]thiadiazine (5e)

Pale-yellow solid; yield: 74%; M.p.: 115–118 ^o^C; ^1^H-NMR (400 MHz, DMSO-*d*_6_): δ 7.87–7.93 (m, 2 H), 7.39 (s, 3 H), 4.40 (s, 2 H), 3.94 (s, 3 H), 3.85 (s, 6 H), 3.75 (s, 3 H); ^13^C-NMR (100 MHz, DMSO-*d*_6_): δ 155.0, 153.4 (2 C), 151.4, 150.8, 139.6, 126.3, 125.4, 121.7, 115.1, 114.9, 114.5, 105.7 (2 C), 60.7, 56.9, 56.4 (2 C), 22.8; ESI-MS: m/z = 431.1 [M + H]^+^, 453.1 [M + Na]^+^.

### 3-(3,4,5-Trimethoxyphenyl)-6-(3-nitro-4-methoxyphenyl)-7H-[1,2,4]triazolo[3,4-b][1,3,4]thiadiazine (5f)

Maroon solid; yield: 68%; M.p.: 212–213 ^o^C; ^1^H-NMR (600 MHz, DMSO-*d*_6_): δ 8.56 (s, 1 H), 8.31 (d, *J* = 8.0 Hz, 1 H), 7.59 (d, *J* = 8.0 Hz, 1 H), 7.38 (s, 2 H), 4.44 (s, 2 H), 4.03 (s, 3 H), 3.85 (s, 6 H), 3.75 (s, 3 H); ^13^C-NMR (150 MHz, DMSO-*d*_6_): δ 154.8, 154.3, 153.3 (2 C), 151.4, 142.6, 139.8, 139.6, 133.7, 125.9, 124.4, 121.5, 115.5, 105.6 (2 C), 60.6, 57.7, 56.3 (2 C), 22.8; ESI-MS: m/z = 458.3 [M + H]^+^, 480.2 [M + Na]^+^.

### 3-(3,4,5-Trimethoxyphenyl)-6-(3-amino-4-methoxyphenyl)-7H-[1,2,4]triazolo[3,4-b][1,3,4]thiadiazine (5g)

Yellow solid; yield: 65%; M.p.: 112–115 ^o^C; ^1^H-NMR (400 MHz, DMSO-*d*_6_): δ 7.40–7.41 (m, 3 H), 7.26 (dd, *J* = 8.4 Hz, *J* = 2.2 Hz, 1 H), 6.95 (d, *J* = 8.4 Hz, 1 H), 5.03 (s, 2 H), 4.33 (s, 2 H), 3.85 (s, 3 H), 3.84 (s, 6 H), 3.75 (s, 3 H); ^13^C-NMR (100 MHz, DMSO-*d*_6_): δ 156.2, 153.3 (2 C), 151.3, 150.3, 143.0, 139.4, 138.8, 125.9, 121.8, 117.8, 111.0, 110.4, 105.5 (2 C), 60.6, 56.3 (2 C), 56.0, 22.7; ESI-MS: m/z = 428.3 [M + H]^+^, 450.3 [M + Na]^+^.

### 3-(3,4,5-Trimethoxyphenyl)-6-(3-benzyloxy-4-methoxyphenyl)-7H-[1,2,4]triazolo[3,4-b][1,3,4]thiadiazine (5h)

Brown solid; yield: 64%; M.p.: 93–94 ^o^C; ^1^H-NMR (400 MHz, CDCl_3_): δ 7.58 (s, 1 H), 7.48 (d, *J* = 8.0 Hz, 1 H), 7.39–7.43 (m, 4 H), 7.30–7.33 (m, 3 H), 6.97 (d, *J* = 8.2 Hz, 1 H), 5.14 (s, 2 H), 3.96 (s, 3 H), 3.91–3.93 (m, 5 H), 3.87 (s, 6 H); ^13^C-NMR (100 MHz, CDCl_3_): δ 153.5, 153.2(3 C), 148.7, 139.9, 136.2, 128.7 (2 C), 128.3 (2 C), 127.5 (2 C), 125.9, 121.8 (2 C), 121.5, 112.5, 111.3, 105.7 (2 C), 71.6, 61.0, 56.4 (2 C), 56.2, 23.0; ESI-MS: m/z = 519.2 [M + H]^+^, 541.1 [M + Na]^+^.

### 3-(3,4,5-Trimethoxyphenyl)-6-(3-hydroxy-4-methoxyphenyl)-7H-[1,2,4]triazolo[3,4-b][1,3,4]thiadiazine (5i)

Yellow solid; yield: 60%; M.p.: 95–97 ^o^C; ^1^H-NMR (400 MHz, DMSO-*d*_6_): δ 9.53 (s, 1 H), 7.56 (d, *J* = 2.2 Hz, 1 H), 7.50 (dd, *J* = 8.5 Hz, *J* = 2.2 Hz, 1 H), 7.38 (s, 2 H), 7.09 (d, *J* = 8.5 Hz, 1 H), 4.36 (s, 2 H), 3.86 (s, 3 H), 3.85 (s, 6 H), 3.76 (s, 3 H); ^13^C-NMR (100 MHz, DMSO-*d*_6_): δ 155.8, 153.4, 151.9, 151.5, 147.5, 143.0, 139.5, 126.0, 121.8, 120.8, 113.6, 112.1, 105.6, 60.7, 56.4 (2 C), 56.2, 22.7; ESI-MS: m/z = 429.1 [M + H]^+^, 451.1 [M + Na]^+^.

### 3-(3,4-Methylenedioxyphenyl)-6-(3-fluoro-4-methoxyphenyl)-7H-[1,2,4]triazolo[3,4-b][1,3,4]thiadiazine (6a)

Pale-yellow solid; yield: 76%; M.p.: 93–95 ^o^C; ^1^H-NMR (400 MHz, DMSO-*d*_6_): δ 7.85 (s, 1 H), 7.83 (s, 1 H), 7.54 (dd, *J* = 8.2 Hz, *J* = 1.6 Hz, 1 H), 7.50 (d, *J* = 1.6 Hz, 1 H), 7.35–7.39 (m, 1 H), 7.12 (d, *J* = 8.2 Hz, 1 H), 6.13 (s, 2 H), 4.37 (s, 2 H), 3.94 (s, 3 H); ^13^C-NMR (100 MHz, DMSO-*d*_6_): δ 155.1, 151.7, 150.7, 149.4, 148.0, 142.6, 126.3, 125.5, 123.1, 120.1, 115.3, 115.1, 114.5, 109.1, 108.2, 102.2, 56.8, 22.9; ESI-MS: m/z = 385.1 [M + H]^+^, 407.1 [M + Na]^+^.

### 3-(3,4-Methylenedioxyphenyl)-6-(3-nitro-4-methoxyphenyl)-7H-[1,2,4]triazolo[3,4-b][1,3,4]thiadiazine (6b)

Pale-yellow solid; yield: 80%; M.p.: 90–92 ^o^C; ^1^H-NMR (600 MHz, DMSO-*d*_6_): δ 8.49 (s, 1 H), 8.27 (d, *J* = 7.6 Hz, 1 H), 7.58 (d, *J* = 7.6 Hz, 1 H), 7.54 (d, *J* = 7.2 Hz, 1 H), 7.50 (s, 1 H), 7.11 (d, *J* = 7.2 Hz, 1 H), 6.13 (s, 2 H), 4.42 (s, 2 H), 4.03 (s, 3 H); ^13^C-NMR (150 MHz, DMSO-*d*_6_): δ 154.8, 154.3, 151.7, 149.4, 147.9, 142.5, 139.7, 133.5, 126.0, 124.8, 123.1, 119.9, 115.6, 109.0, 108.2, 102.1, 57.7, 22.9; ESI-MS: m/z = 412.1 [M + H]^+^, 434.1 [M + Na]^+^.

### 3-(3,4-Methylenedioxyphenyl)-6-(3-amino-4-methoxyphenyl)-7H-[1,2,4]triazolo[3,4-b][1,3,4]thiadiazine (6c)

Yellow solid; yield: 78%; M.p.: 88–90 ^o^C; ^1^H-NMR (400 MHz, DMSO-*d*_6_): δ 7.58 (dd, *J* = 8.2 Hz, *J* = 1.4 Hz, 1 H), 7.49 (d, *J* = 1.4 Hz, 1 H), 7.28 (d, *J* = 2.1 Hz, 1 H), 7.22 (dd, *J* = 8.4 Hz, *J* = 2.1 Hz, 1 H), 7.12 (d, *J* = 8.2 Hz, 1 H), 6.95 (d, *J* = 8.4 Hz, 1 H), 6.13 (s, 2 H), 5.06 (s, 2 H), 4.30 (s, 2 H), 3.85 (s, 3 H); ^13^C-NMR (100 MHz, DMSO-*d*_6_): δ 156.4, 151.5, 150.2, 149.2, 147.9, 142.9, 138.6, 126.1, 122.9, 120.3, 117.7, 111.4, 110.5, 109.0, 108.1, 102.0, 56.0, 23.0; ESI-MS: m/z = 382.0 [M + H]^+^.

### 3-(3,4-Methylenedioxyphenyl)-6-(3-benzyloxy-4-methoxyphenyl)-7H-[1,2,4]triazolo[3,4-b][1,3,4]thiadiazine (6d)

Pale-yellow solid; yield: 81%; M.p.: 86–88 ^o^C; ^1^H-NMR (400 MHz, CDCl_3_): δ 7.64–7.65 (m, 2 H), 7.59 (d, *J* = 1.6 Hz, 1 H), 7.39–7.45 (m, 3 H), 7.30–7.37 (m, 3 H), 6.96 (d, *J* = 8.4 Hz, 1 H), 6.88 (d, *J* = 8.4 Hz, 1 H), 6.02 (s, 2 H), 5.19 (s, 2 H), 3.96 (s, 3 H), 3.90 (s, 2 H); ^13^C-NMR (100 MHz, CDCl_3_): δ 153.3, 153.1, 149.3, 148.6, 147.8, 136.4, 128.7 (3 C), 128.2, 127.5 (2 C), 127.4, 125.8, 123.0, 121.7, 120.1, 111.8, 111.1, 108.5 (2 C), 101.5, 71.1, 56.2, 22.9; ESI-MS: m/z = 473.0 [M + H]^+^, 945.1 [2 M + H]^+^.

### 3-(3,4-Methylenedioxyphenyl)-6-(3-hydroxy-4-methoxyphenyl)-7H-[1,2,4]triazolo[3,4-b][1,3,4]thiadiazine (6e)

Yellow solid; yield: 68%; M.p.: 85–87 ^o^C; ^1^H-NMR (400 MHz, DMSO-*d*_6_): δ 9.55 (s, 1 H), 7.56 (dd, *J* = 8.2 Hz, *J* = 1.6 Hz, 1 H), 7.50 (d, *J* = 1.6 Hz, 1 H), 7.45–7.47 (m, 2 H), 7.08–7.13 (m, 2 H), 6.14 (s, 2 H), 4.33 (s, 2 H), 3.86 (s, 3 H); ^13^C-NMR (100 MHz, DMSO-*d*_6_): δ 156.0, 151.8, 151.6, 149.3, 148.0, 147.3, 142.9, 126.1, 123.0, 120.8, 120.3, 113.9, 112.2, 109.1, 108.2, 102.1, 56.2, 22.9; ESI-MS: m/z = 383.1 [M + H]^+^, 405.1 [M + Na]^+^.

### 3-(3,4-Dimethoxyphenyl)-6-(3-nitro-4-methoxyphenyl)-7H-[1,2,4]triazolo[3,4-b][1,3,4]thiadiazine (6f)

Yellow solid; yield: 78%; M.p.: 190–192 ^o^C; ^1^H-NMR (600 MHz, DMSO-*d*_6_): δ 8.53 (d, *J* = 2.3 Hz, 1 H), 8.30 (dd, *J* = 8.9 Hz, *J* = 2.3 Hz, 1 H), 7.64 (d, *J* = 1.8 Hz, 1 H), 7.60 (dd, *J* = 8.4 Hz, *J* = 1.8 Hz, 1 H), 7.58 (d, *J* = 8.9 Hz, 1 H), 7.14 (d, *J* = 8.4 Hz, 1 H), 4.43 (s, 2 H), 4.03 (s, 3 H), 3.84 (s, 3 H), 3.83 (s, 3 H); ^13^C-NMR (150 MHz, DMSO-*d*_6_): δ 154.7, 154.1, 151.7, 150.9, 148.9, 142.2, 139.8, 133.6, 126.0, 124.5, 121.4, 118.7, 115.5, 112.0, 111.3, 56.0, 55.9, 55.3, 22.8; ESI-MS: m/z = 428.1 [M + H]^+^, 450.1 [M + Na]^+^.

### 3-(3,4-Dimethoxyphenyl)-6-(3-amino-4-methoxyphenyl)-7H-[1,2,4]triazolo[3,4-b][1,3,4]thiadiazine (6g)

Pale-yellow solid; yield: 68%; M.p.: 213–215 ^o^C; ^1^H-NMR (600 MHz, DMSO-*d*_6_): δ 7.66 (d, *J* = 8.9 Hz, 1 H), 7.65 (s, 1 H), 7.36 (s, 1 H), 7.24 (dd, *J* = 8.5 Hz, *J* = 1.6 Hz, 1 H), 7.16 (d, *J* = 8.9 Hz, 1 H), 6.95 (d, *J* = 8.5 Hz, 1 H), 5.07 (s, 2 H), 4.32 (s, 2 H), 3.86 (s, 3 H), 3.84 (s, 3 H), 3.83 (s, 3 H); ^13^C-NMR (150 MHz, DMSO-*d*_6_): δ 156.2, 151.5, 150.7, 150.2, 148.9, 142.6, 138.7, 126.1, 121.2, 119.0, 117.7, 112.1, 111.2 (2 C), 110.4, 55.9, 55.8, 55.3, 22.8; ESI-MS: m/z = 398.4 [M + H]^+^, 420.3 [M + Na]^+^.

### 3-(3-Methoxyphenyl)-6-(3-nitro-4-methoxyphenyl)-7H-[1,2,4]triazolo[3,4-b][1,3,4]thiadiazine (6h)

White solid; yield: 83%; M.p.: 207–208 ^o^C; ^1^H-NMR (600 MHz, DMSO-*d*_6_): δ 8.51 (d, *J* = 2.1 Hz, 1 H), 8.28 (dd, *J* = 8.9 Hz, *J* = 2.1 Hz, 1 H), 7.57–7.60 (m, 3 H), 7.46–7.49 (m, 1 H), 7.12 (dd, *J* = 8.4 Hz, *J* = 2.1 Hz, 1 H), 4.45 (s, 2 H), 4.03 (s, 3 H), 3.83 (s, 3 H); ^13^C-NMR (150 MHz, DMSO-*d*_6_): δ 159.6, 154.8, 154.4, 151.7, 142.9, 139.7, 133.6, 130.3, 127.4, 125.9, 124.7, 120.6, 116.7, 115.6, 113.2, 57.7, 55.6, 22.9; ESI-MS: m/z = 398.1 [M + H]^+^.

### 3-(3-Methoxyphenyl)-6-(3-amino-4-methoxyphenyl)-7H-[1,2,4]triazolo[3,4-b][1,3,4]thiadiazine (6i)

Red-brown solid; yield: 65%; M.p.: 138–140 ^o^C; ^1^H-NMR (600 MHz, DMSO-*d*_6_): δ 7.64 (d, *J* = 7.8 Hz, 1 H), 7.60 (s, 1 H), 7.48–7.51 (m, 1 H), 7.34 (d, *J* = 2.2 Hz, 1 H), 7.24 (dd, *J* = 8.4 Hz, *J* = 2.2 Hz, 1 H), 7.12 (dd, *J* = 8.2 Hz, *J* = 2.1 Hz, 1 H), 6.95 (d, *J* = 8.4 Hz, 1 H), 5.07 (s, 2 H), 4.34 (s, 2 H), 3.86 (s, 3 H), 3.83 (s, 3 H); ^13^C-NMR (150 MHz, DMSO-*d*_6_): δ 159.6, 156.5, 151.5, 150.2, 143.3, 138.7, 130.3, 127.7, 126.0, 120.5, 117.8, 116.5, 113.1, 111.3, 110.4, 55.9, 55.6, 22.9; ESI-MS: m/z = 368.1 [M + H]^+^.

### 3-(4-Methoxyphenyl)-6-(3-nitro-4-methoxyphenyl)-7H-[1,2,4]triazolo[3,4-b][1,3,4]thiadiazine (6j)

Yellow solid; yield: 85%; M.p.: 97–99 ^o^C; ^1^H-NMR (600 MHz, DMSO-*d*_6_): δ 8.50 (s, 1 H), 8.28 (d, *J* = 8.9 Hz, 1 H), 7.95 (d, *J* = 8.4 Hz, 2 H), 7.56 (d, *J* = 8.9 Hz, 1 H), 7.12 (d, *J* = 8.4 Hz, 2 H), 4.43 (s, 2 H), 4.03 (s, 3 H), 3.84 (s, 3 H); ^13^C-NMR (150 MHz, DMSO-*d*_6_): δ 161.1, 154.7, 154.2, 151.8, 142.2, 139.7, 133.5, 129.9 (2 C), 126.0, 124.7, 118.6, 115.5, 114.6 (2 C), 57.6, 55.7, 22.9; ESI-MS: m/z = 398.1 [M + H]^+^, 420.1 [M + Na]^+^.

### 3-(4-Methoxyphenyl)-6-(3-amino-4-methoxyphenyl)-7H-[1,2,4]triazolo[3,4-b][1,3,4]thiadiazine (6k)

Pale-yellow solid; yield: 71%; M.p.: 214–215 ^o^C; ^1^H-NMR (600 MHz, DMSO-*d*_6_): δ 8.00 (d, *J* = 8.9 Hz, 2 H), 7.32 (d, *J* = 2.3 Hz, 1 H), 7.23 (dd, *J* = 8.5 Hz, *J* = 2.3 Hz, 1 H), 7.14 (d, *J* = 8.9 Hz, 2 H), 6.95 (d, *J* = 8.5 Hz, 1 H), 5.09 (s, 2 H), 4.32(s, 2 H), 3.86 (s, 3 H), 3.85 (s, 3 H); ^13^C-NMR (150 MHz, DMSO-*d*_6_): δ 161.0, 156.3, 151.6, 150.2, 142.5, 138.7, 129.7 (2 C), 126.1, 119.0, 117.8, 114.6 (2 C), 111.3, 110.4, 55.9, 55.7, 22.9; ESI-MS: m/z = 368.3 [M + H]^+^, 390.3 [M + Na]^+^.

### MTT assay

The antiproliferative activities of all of the target compounds and CA-4 were determined by a standard MTT assay following a previously reported method^33^. The SGC-7901, A549 and HT-1080 cell lines were purchased from the American Type Culture Collection (ATCC, Manassas, VA, USA). The *in vitro* antiproliferative activities of CA-4 and all of the target compounds were determined by a MTT (Sigma) assay. Briefly, approximately 3 × 104 cells were seeded in a 96-well plate. After 24 h of incubation at 37 °C, cells were exposed to compounds of differing concentrations for 24 h. After treatment, cells were washed with 1X PBS followed by addition of 100 µL of 0.05% MTT reagent to each well, followed by incubation for 4 h at 37 °C. After incubation, the supernatant from each well was carefully removed and the formazan crystals were dissolved in 100 µL of DMSO. The colour density was measured spectrophotometrically at 490 nm using a microplate reader (SpectraMax Plus384, Molecular Devices Corp., USA). The data were calculated and plotted as percent viability compared to control.

### Tubulin polymerization assay

Tubulin polymerization assay^33^ was conducted with reagents as described in the kit manufacturer (Cytoskeleton, Cat.#BK011P) in a 96-well plate. In brief, tubulin was re-suspended in ice-cold G-PEM buffer (80 mM PIPES, 2 mM MgCl_2_, 0.5 mM EGTA, 1 mM GTP, 20% (v/v) glycerol) and added to wells on a 96-well plate containing the designated concentration of drugs or vehicle. Samples were mixed well, and tubulin assembly was monitored (emission wavelength: 450 ± 20 nm; excitation wavelength: 360 ± 20 nm) at 1 min intervals for 90 min at 37 °C in a SpectraMax 340PC spectrophotometer (Biotek Synergy HT, Winooskin, VT, USA). IC_50_ values were calculated from data at the 20 min time point using GraphPad Prism software. Experiments were repeated three times.

### Immunofluorenscence assay

Immunostaining assay^35^ was carried out to detect microtubule associated tubulin protein after exposure to **6i** and CA-4. The SGC-7901 cells were seeded at a density of 1 × 104 per well on a 24-well plate and grown for 24 h. Cells were treated with CA-4 or **6i** for 12 h. Cells in the control group were treated with culture medium. The control and treated cells were fixed with 4% formaldehyde in PBS for 30 min at -20 °C, then washed twice with PBS and permeabilized with 0.1% (v/v) Triton X-100 in PBS for 5 min. Then, the cells were blocked with 3% bovine serum albumin (BSA) in PBS for 30 min. The primary a-tubulin antibody was diluted (1:100) with 2% BSA in PBS and incubated overnight at 4 °C. The cells were washed with PBS to remove unbound primary antibody and then cells were incubated with FITC-conjugated antimouse secondary antibody, diluted (1:100) with 2% BSA in PBS, for 2 h at 37 °C. The cells were washed with PBS to remove unbound secondary antibody, nucleus was stained with 4,6-diamino-2-phenolindol dihydrochloride (DAPI) and then, immunofluorescence was detected using a fluorescence microscope (Olympus, Tokyo, Japan).

### Cell cycle analysis

Cell cycle analysis studies were performed by following a previously reported method^35^. SGC-7901 cells (8 × 10^4^ cells) were incubated with various concentrations of CA-4, **6i** or 0.05% DMSO for the indicated times. The cells were collected by centrifugation, washed with PBS and fixed in ice-cold 70% ethanol. The fixed cells were harvested by centrifugation and resuspended in 500 µl of PBS containing 1 mg/mL RNase. After 30 min of incubation at 37 °C, the cells were stained with 50 mg/mL propidium iodide (PI) at 4 °C in the dark for 30 min. The samples were then analyzed by FACScan flow cytometry (Becton-Dickinson, Franklin Lakes, NJ, USA). The experiments were repeated at least three times.

### Competitive tubulin-binding assay

For the colchicine competitive binding assay, tubulin was co-incubated with indicated concentrations of MPSP-001 and paclitaxel at 37 °C for 1 h. Then colchicine was added to a final concentration of 5 μmol L^−1^. Fluorescence was determined using a Hitachi F-2500 spectrofluorometer (Tokyo, Japan) at the excitation wavelength of 365 nm and the emission wavelength of 435 nm. Blank values (buffer alone) as the background were subtracted from all samples. Then the inhibition rate (IR) was calculated as follows: IR = F/F_0_ where F_0_ is the fluorescence of 5 μmol L^−1^ colchicine-tubulin complex, and F is the fluorescence of a given concentration of CA-4 or **6i** or taxol (1.6 μmol L^−1^, 5 μmol L^−1^, 15 μmol L^−1^) in competition with 5 μmol L^−1^ colchicine-tubulin complex. Paclitaxel, not binding in the colchicine site of tubulin, was added as a negative control. The experiments were repeated at least three times.

### Molecular docking studies

Molecular docking studies were performed by following a previously reported method^33^. The molecular modeling studies were performed using Accelrys Discovery Studio 3.0. The crystal structure of tubulin complexed with DAMA-colchicine (PDB: 1SA0) was retrieved from the RCSB Protein Data Bank (http://www.rcsb.org/pdb). In the docking process, the protein protocol was prepared via several operations, including the standardization of atom names, insertion of missing atoms in residues and removal of alternate conformations, insertion of missing loop regions based on SEQRES data, optimization of short and medium sized loop regions with the Looper Algorithm, minimization of remaining loop regions, calculation of pK, and protonation of the structure. The receptor model was then typed with the CHARMm force field, and a binding sphere with a radius of 9.0 Å was defined with the original ligand (DAMA-colchicine) as the binding site. The **6i**, CA-4 and vinylogous CA-4 were drawn with Chemdraw and fully minimized using the CHARMm force field. Finally, **6i**, CA-4 and vinylogous CA-4 were docked into the binding site using the CDOCKER protocol with the default settings.

## Electronic supplementary material

Supplementary information
