# Supplementary material for: Design, synthesis and structure-activity relationship of 3,6-diaryl-7H-[1,2,4]triazolo[3,4-b][1,3,4]thiadiazines as novel tubulin inhibitors
Source: Sci Rep. 2017 Sep 20;7:11997. doi: 10.1038/s41598-017-10860-7 (PMC5607265; doi:10.1038/s41598-017-10860-7)

**Design, synthesis and structure-activity relationship of 3,6-diaryl-7*H*-****[1,2,4]triazolo[3,4-b][1,3,4]thiadiazines as** **novel tubulin inhibitors**

**Qile Xu^1*^, Kai Bao^1*^, Maolin Sun^1^,** **Jingwen Xu^2^, Yueting Wang^1^, Haiqiu Tian^1^, Daiying Zuo^2^, Qi Guan^1^, Yingliang Wu^2^ & Weige Zhang^1^**

^1^ Key Laboratory of Structure-Based Drug Design and Discovery, Ministry of Education, Shenyang Pharmaceutical University, 103 Wenhua Road, Shenhe District, Shenyang 110016, China.

^2^ Department of Pharmacology, Shenyang Pharmaceutical University, 103 Wenhua Road, Shenhe District, Shenyang 110016, China.

^*^ These authors contributed equally to this work.

Correspondence and requests for materials should be addressed to W. Z. (email: zhangweige@syphu.edu.cn) or Y. W. (email: [yingliang_1016@163.com](mailto:yingliang_1016@163.com)).

**List of content**

**1. Synthesis**

**(1). General procedure for the preparation of compounds 11**

**(2). General procedure for the preparation of compounds 13**

**2. Biology**

**(1). MTT assay**

**(2). Tubulin polymerization assay**

**(3). Immunofluorenscence assay**

**(4). Cell cycle analysis**

**(5). Competitive tubulin-binding assay.**

**(6). Molecular docking** **studies**

**3. Characterization for all intermediates and target compounds.**

**(1). ^1^H-NMR for all intermediates**

**(2). ^1^H-NMR and ^13^C-NMR spectra of all target compounds.**

**1. Synthesis**

**(1). General procedure for the preparation of compounds 11**

To a solution of substituted benzoic acid **7** in methanol, concentrated sulphuric acid was added slowly at room temperature and heated by a microwave irradiation at 250 W, 70 °C, for 40 min. After the reaction completed, the mixture was poured into cold water. Precipitated solid was filtered, washed with water and dried. Then, the corresponding ester **8** was refluxed with 80% hydrazine hydrate in methanol to get hydrazide **9** under microwave irradiation at 250 W, 70 °C, for 120 min. The hydrazide **9** was stirred with potassium hydroxide in absolute methanol and carbon disulfide was slowly added. The mixture was stirred 24h at room temperature and the solid was precipitated, dried to afford compound **10**. The solid was refluxed with 80% hydrazine hydrate in water under microwave irradiation at 250 W, 100 °C, for 60 min. Then the reaction mixture was acidified with concentrated hydrochloric acid and the white precipitate was filtered, washed with cold water and recrystallized from aqueous methanol to obtain the intermediate **11**.

4-amino-5-(2,3,4-trimethoxyphenyl)-4*H*-1,2,4-triazole-3-thiol. White solid; ^1^H-NMR (600 MHz, DMSO-d_6_): δ 13.81 (s, 1H), 7.18 (d, *J* = 8.7 Hz, 1H), 6.92 (d, *J* = 8.7 Hz, 1H), 5.48 (s, 2H), 3.86 (s, 3H), 3.79 (s, 3H), 3.78 (s, 3H).

4-amino-5-(3,4-dimethoxyphenyl)-4*H*-1,2,4-triazole-3-thiol. White solid; ^1^H-NMR (600 MHz, DMSO-d_6_): δ 13.51 (s, 1H), 7.32 (dd, *J* = 8.3 Hz, *J* = 1.9 Hz, 1H), 7.29 (d, *J* = 1.9 Hz, 1H), 7.05 (d, *J* = 8.3 Hz, 1H), 5.75 (s, 2H), 3.81 (s, 3H), 3.80 (s, 3H).

4-amino-5-(3,4,5-trimethoxyphenyl)-4*H*-1,2,4-triazole-3-thiol. White solid; ^1^H-NMR (600 MHz, DMSO-d_6_): δ 13.93 (s, 1H), 7.34 (s, 2H), 5.84 (s, 2H), 3.82 (s, 6H), 3.73 (s, 3H).

4-amino-5-(3-methoxyphenyl)-4*H*-1,2,4-triazole-3-thiol. White solid; ^1^H-NMR (600 MHz, DMSO-d_6_): δ 13.95 (s, 1H), 7.60 (d, *J* = 2.4 Hz, 1H), 7.59 (d, *J* = 7.8 Hz, 1H), 7.44 (m, 1H), 7.11 (dd, *J* = 8.2 Hz, *J* = 2.4 Hz, 1H), 5.81 (s, 2H), 3.80 (s, 3H).

4-amino-5-(4-methoxyphenyl)-4*H*-1,2,4-triazole-3-thiol. White solid; ^1^H-NMR (600 MHz, DMSO-d_6_): δ 13.84 (s, 1H), 7.94 (d, *J* = 8.9 Hz, 2H), 7.59 (d, *J* = 8.9 Hz, 2H), 5.71 (s, 2H), 3.79 (s, 3H).

4-amino-5-(3,4-Methylenedioxyphenyl)-4*H*-1,2,4-triazole-3-thiol. White solid; ^1^H-NMR (600 MHz, DMSO-d_6_): δ 14.65 (s, 1H), 7.43 (dd, *J* = 8.1 Hz, *J* = 1.7 Hz, 1H), 7.35 (d, *J* = 1.7 Hz, 1H), 7.11 (d, *J* = 8.1 Hz, 1H), 6.16 (s, 2H).

**(2). General procedure for the preparation of compounds 13**

A solution of appropriately substituted acetophenone **12** (10 mmol) in 40 mL of chloroform was added in one portion to a vigorously stirred, refluxing suspension of cupric bromide (21 mmol) in 30 mL of ethyl acetate. The reaction was practically complete after refluxing for 1.5 h, as indicated by the conversion of cupric bromide (black) into Copper bromide (white), lack of hydrogen bromide evolution. The solids were removed by filtration, washing with ethyl acetate. The residue from evaporation was distributed between ethyl acetate and saturated NaHCO_3_. The organic layers were washed with saturated sodium chloride (200 mL), dried (Na_2_SO_4_), and evaporated in vacuo. The crude residue was passed through a quick column, eluting with ethyl acetate-cyclohexane and used as such for the next step.

2-bromo-1-(4-fluorophenyl)ethanone. White solid; ^1^H-NMR (600 MHz, CDCl_3_): δ 8.02 (m, 2H), 7.16 (m, 2H), 4.41 (s, 2H).

2-bromo-1-(4-chlorophenyl)ethanone. White solid; ^1^H-NMR (600 MHz, CDCl_3_): δ 7.93 (d, *J* = 8.6 Hz, 2H), 7.47 (d, *J* = 8.6 Hz, 2H), 4.40 (s, 2H).

2-bromo-1-(4-bromophenyl)ethanone. White solid; ^1^H-NMR (600 MHz, CDCl_3_): δ 7.85 (d, *J* = 8.5 Hz, 2H), 7.64 (d, *J* = 8.5 Hz, 2H), 4.40 (s, 2H).

2-bromo-1-(*p*-tolyl)ethanone. White solid; ^1^H-NMR (600 MHz, CDCl_3_): δ 7.88 (d, *J* = 8.2 Hz, 2H), 7.29 (d, *J* = 8.2 Hz, 2H), 4.43 (s, 2H), 2.43 (s, 3H).

2-bromo-1-(4-(trifluoromethyl)phenyl)ethanone. White solid; ^1^H-NMR (600 MHz, CDCl_3_): δ 8.10 (d, *J* = 8.2 Hz, 2H), 7.77 (d, *J* = 8.2 Hz, 2H), 4.45 (s, 2H).

2-bromo-1-(4-methoxyphenyl)ethanone. Yellow solid; ^1^H-NMR (600 MHz, CDCl_3_): δ 7.96 (d, *J* = 8.8 Hz, 2H), 6.95 (d, *J* = 8.8 Hz, 2H), 4.39 (s, 2H), 3.88 (s, 3H).

2-bromo-1-(4-(methylthio)phenyl)ethanone. Yellow solid; ^1^H-NMR (600 MHz, CDCl_3_): δ 7.89 (d, *J* = 8.6 Hz, 2H), 7.28 (d, *J* = 8.6 Hz, 2H), 4.40 (s, 2H), 2.53 (s, 3H).

2-bromo-1-(3-fluoro-4-methoxyphenyl)ethanone. White solid; ^1^H-NMR (600 MHz, CDCl_3_): δ 7.77 (d, *J* = 8.6 Hz, 1H), 7.72 (dd, *J* = 11.6 Hz, *J* = 2.1 Hz, 1H), 7.01 (m, 1H), 4.37 (s, 2H), 3.96 (s, 3H).

2-bromo-1-(4-methoxy-3-nitrophenyl)ethanone. White solid; ^1^H-NMR (600 MHz, CDCl_3_): δ 8.46 (d, *J* = 2.2 Hz, 1H), 8.20 (dd, *J* = 8.9 Hz, *J* = 2.2 Hz, 1H), 7.19 (d, *J* = 8.9 Hz, 1H), 4.39 (s, 2H), 4.06 (s, 3H).

2-bromo-1-(3-(benzyloxy)-4-methoxyphenyl)ethanone. White solid; ^1^H-NMR (600 MHz, CDCl_3_): δ 7.61 (dd, *J* = 8.4 Hz, *J* = 1.9Hz, 1H), 7.58 (d, *J* = 1.9 Hz, 1H), 7.46 (d, *J* = 7.5 Hz, 2H), 7.38 (m, 2H), 7.32 (m, 1H), 6.92 (d, *J* = 8.4 Hz, 1H), 5.19 (s, 2H), 4.36 (s, 2H), 3.95 (s, 3H).

2-bromo-1-(3,4-difluorophenyl)ethanone. White solid; ^1^H-NMR (600 MHz, CDCl_3_): δ 7.83 (m, 1H), 7.77 (m, 1H), 7.28 (m, 1H), 4.38 (s, 2H).

**2. Biology**

**(1). MTT assay**

The SGC-7901, A549 and HT-1080 cell lines were purchased from the American Type Culture Collection (ATCC, Manassas, VA, USA). The in vitro antiproliferative activities of CA-4 and all of the target compounds were determined by an MTT (Sigma) assay. Briefly, approximately 3 × 10^4^ cells were seeded in a 96-well plate. After 24 h of incubation at 37 °C, cells were exposed to compounds of differing concentrations for 24 h. After treatment, cells were washed with 1X PBS followed by addition of 100 µL of 0.05% MTT reagent to each well, followed by incubation for 4 h at 37 °C. After incubation, the supernatant from each well was carefully removed and the formazan crystals were dissolved in 100 µL of DMSO. The colour density was measured spectrophotometrically at 490 nm using a microplate reader (SpectraMax Plus384, Molecular Devices Corp., USA). The data were calculated and plotted as percent viability compared to control.

Table S1. Antiproliferative activities of **6i**, vinylogous CA-4 (*Z,E*) and CA-4.

|  | IC_50_^a^ (μM) | | | |
| --- | --- | --- | --- | --- |
|  | SGC-7901 | A549 | HT-1080 | HCT116 ^b^ |
| **6i** | 0.011 | 0.015 | 0.014 | - |
| vinylogous CA-4 (*Z,E*) | - | - | - | 0.163 |
| CA-4 | 0.011 | 0.009 | 0.013 | 0.016 |

^a^ IC_50_: 50% inhibitory concentration. ^b^ Values from Ref (Org. Biomol. Chem. 2005, 3, 2657-2660).

**(2). Tubulin polymerization assay**

Tubulin polymerization assay was conducted with reagents as described in the kit manufacturer (Cytoskeleton, Cat.#BK011P) in a 96-well plate. In brief, tubulin was re-suspended in ice-cold G-PEM buffer (80 mM PIPES, 2 mM MgCl_2_, 0.5 mM EGTA, 1 mM GTP, 20% (v/v) glycerol) and added to wells on a 96-well plate containing the designated concentration of drugs or vehicle. Samples were mixed well, and tubulin assembly was monitored (emission wavelength: 450±20 nm; excitation wavelength: 360±20 nm) at 1 min intervals for 90 min at 37 °C in a SpectraMax 340PC spectrophotometer (Biotek Synergy HT, Winooskin, VT, USA). IC_50_ values were calculated from data at the 20 min timepoint using GraphPad Prism software. Experiments were repeated three times.

**(3). Immunofluorenscence assay**

Immunostaining was carried out to detect microtubule associated tubulin protein after exposure to **6i** and CA-4. The SGC-7901 cells were seeded at a density of 1 × 10^4^ per well on a 24-well plate and grown for 24 h. Cells were treated with CA-4 or **6i** for 12 h. Cells in the control group were treated with culture medium. The control and treated cells were fixed with 4% formaldehyde in PBS for 30 min at -20 °C, then washed twice with PBS and permeabilized with 0.1% (v/v) Triton X-100 in PBS for 5 min. Then, the cells were blocked with 3% bovine serum albumin (BSA) in PBS for 30 min. The primary a-tubulin antibody was diluted (1:100) with 2% BSA in PBS and incubated overnight at 4 °C. The cells were washed with PBS to remove unbound primary antibody and then cells were incubated with FITC-conjugated antimouse secondary antibody, diluted (1:100) with 2% BSA in PBS, for 2 h at 37 °C. The cells were washed with PBS to remove unbound secondary antibody, nucleus was stained with 4,6-diamino-2-phenolindol dihydrochloride (DAPI) and then, immunofluorescence was detected using a fluorescence microscope (Olympus, Tokyo, Japan).

**(4). Cell cycle analysis**

SGC-7901 cells (8 × 10^4^ cells) were incubated with various concentrations of CA-4 , **6i** or 0.05% DMSO for the indicated times. The cells were collected by centrifugation, washed with PBS and fixed in ice-cold 70% ethanol. The fixed cells were harvested by centrifugation and resuspended in 500 µl of PBS containing 1 mg/mL RNase. After 30 min of incubation at 37°C, the cells were stained with 50 mg/mL propidium iodide (PI) at 4°C in the dark for 30 min. The samples were then analyzed by FACScan flow cytometry (Becton-Dickinson, Franklin Lakes, NJ, USA). The experiments were repeated at least three times.


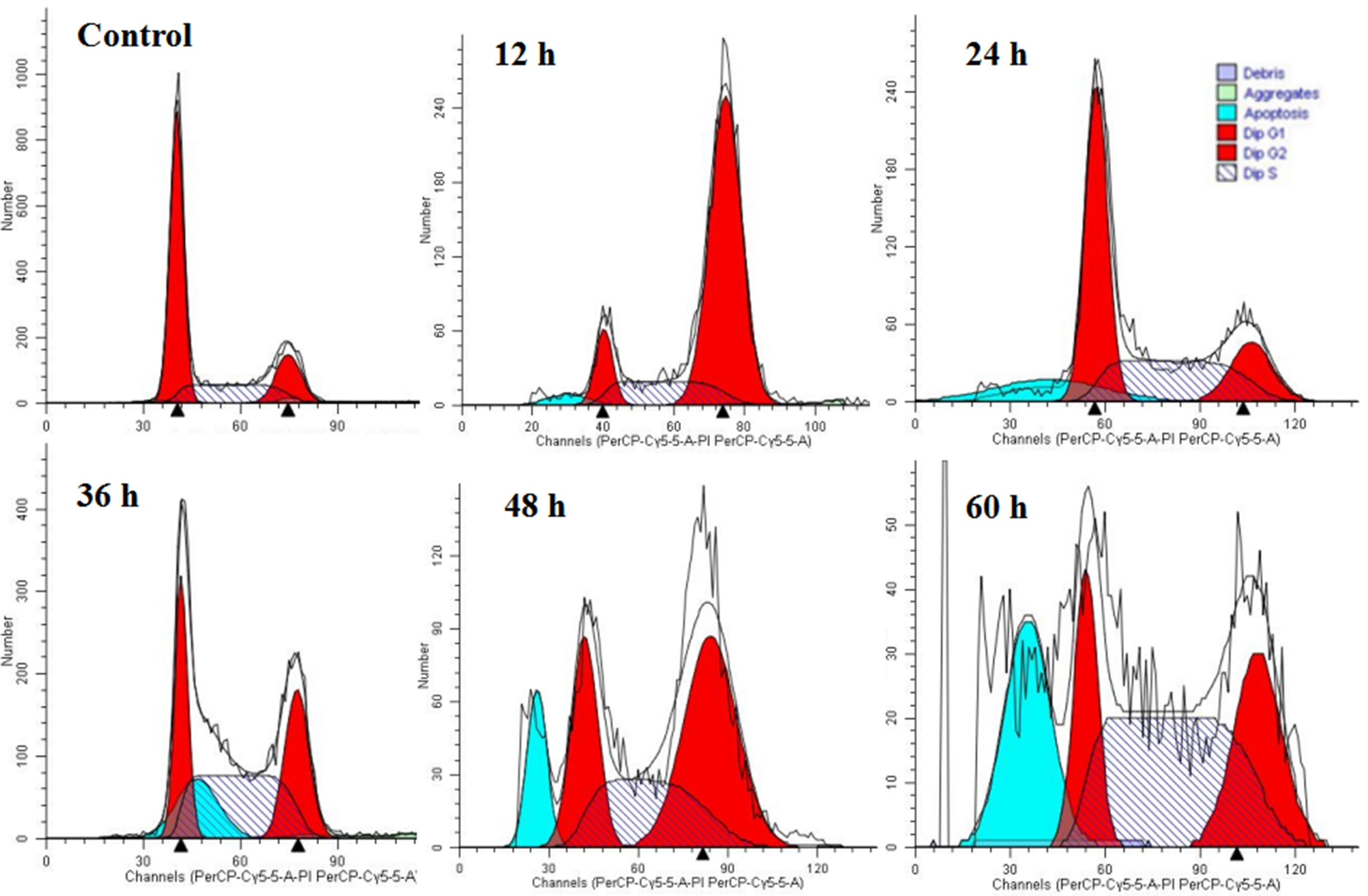


**Figure S1** Compound **6i** induced G2/M phase arrest in a time-dependent manner. SGC-7901 cells were treated with **6i** (0.022 µM) for time 12, 24, 36, 48 and 60 h, then stained with PI and subjected to flow cytometric analysis.

Cell cycle studies performed in SGC-7901 cells showed that **6i** (0.022 µM) treatment induced cell arrest in G_2_/M phase from 0 to 12 h and preceding apoptosis characterized as sub-G_1_ phase increase from 24 to 60 h. As given in Figure 6, as time went on, compound **6i** was found to significantly increase the percentage of cells in the sub-G_1_ and G_2_/M phases, with an accompanying decrease in cells in the G_0_/G_1_ and S phases. These results suggested that **6i** induced SGC-7901 cells arrest in G_2_/M phase preceding apoptosis and **6i** caused subsequent apoptosis after G_2_/M cell-cycle arrest in SGC-7901 cells.

**(5). Competitive tubulin-binding assay.** For the colchicine competitive binding assay, tubulin was co-incubated with indicated concentrations of MPSP-001 and paclitaxel at 37 °C for 1 h. Then colchicine was added to a final concentration of 5 μmol L^−1^. Fluorescence was determined using a Hitachi F-2500 spectrofluorometer (Tokyo, Japan) at the excitation wavelength of 365 nm and the emission wavelength of 435 nm. Blank values (buffer alone) as the background were subtracted from all samples. Then the inhibition rate (IR) was calculated as follows: IR = F/F_0_ where F_0_ is the fluorescence of 5 μmol L^−1^ colchicine-tubulin complex, and F is the fluorescence of a given concentration of CA-4 or **6i** or taxol (1.6 μmol L^−1^, 5 μmol L^−1^, 15 μmol L^−1^) in competition with 5 μmol L^−1^ colchicine-tubulin complex. Paclitaxel, not binding in the colchicine site of tubulin, was added as a negative control. The experiments were repeated at least three times.

**(6). Molecular docking** **studies**

The molecular modeling studies were performed using Accelrys Discovery Studio 3.0. The crystal structure of tubulin complexed with DAMA-colchicine (PDB: 1SA0) was retrieved from the RCSB Protein Data Bank (http://www.rcsb.org/pdb). In the docking process, the protein protocol was prepared via several operations, including the standardization of atom names, insertion of missing atoms in residues and removal of alternate conformations, insertion of missing loop regions based on SEQRES data, optimization of short and medium sized loop regions with the Looper Algorithm, minimization of remaining loop regions, calculation of pK, and protonation of the structure. The receptor model was then typed with the CHARMm force field, and a binding sphere with a radius of 9.0 Å was defined with the original ligand (DAMA-colchicine) as the binding site. The **6i**, CA-4 and vinylogous CA-4 were drawn with Chemdraw and fully minimized using the CHARMm force field. Finally, **6i**, CA-4 and vinylogous CA-4 were docked into the binding site using the CDOCKER protocol with the default settings.

**3. Characterization for all intermediates and target compounds.**

**(1). ^1^H-NMR for all intermediates**

4-amino-5-(2,3,4-trimethoxyphenyl)-4*H*-1,2,4-triazole-3-thiol.


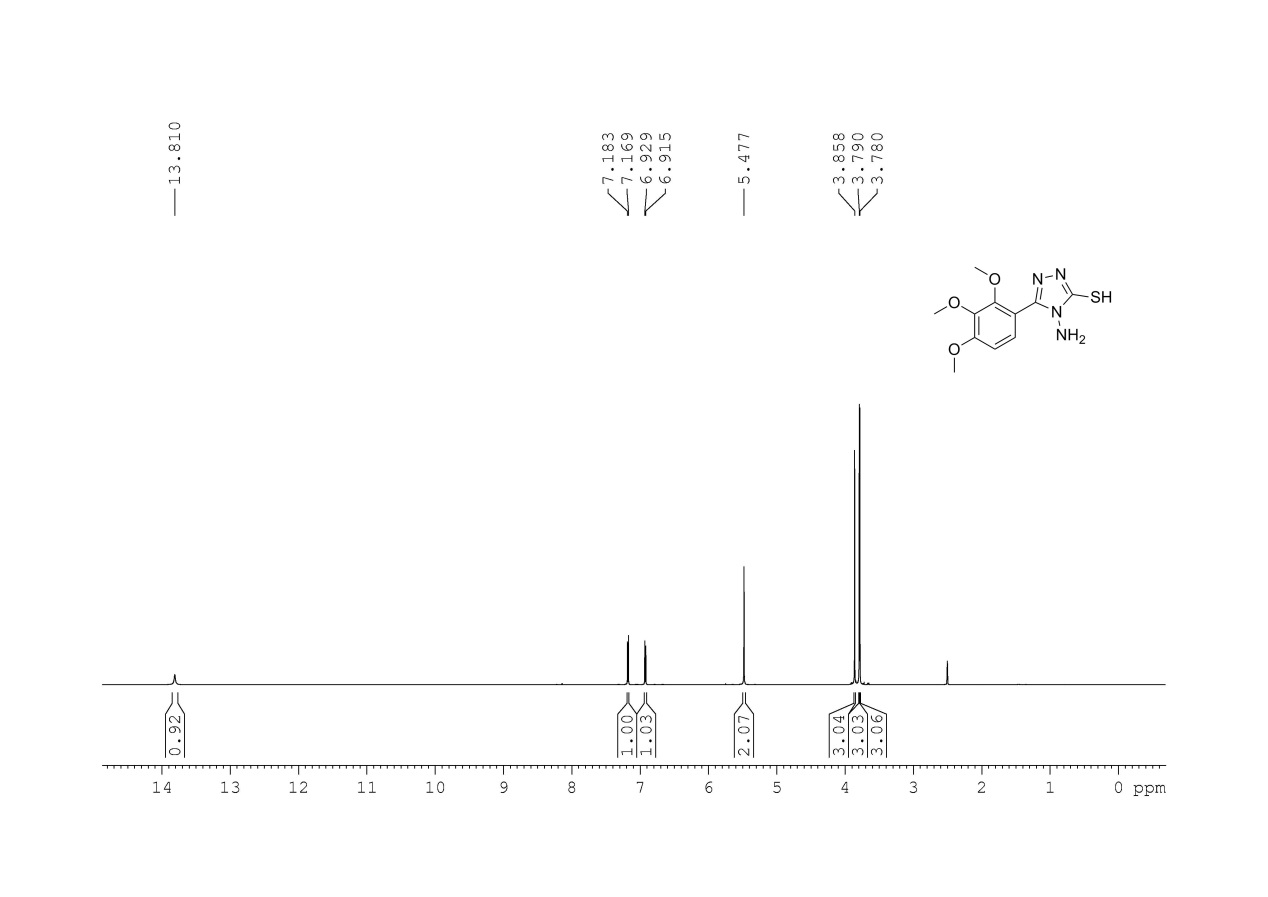


4-amino-5-(3,4-dimethoxyphenyl)-4*H*-1,2,4-triazole-3-thiol.
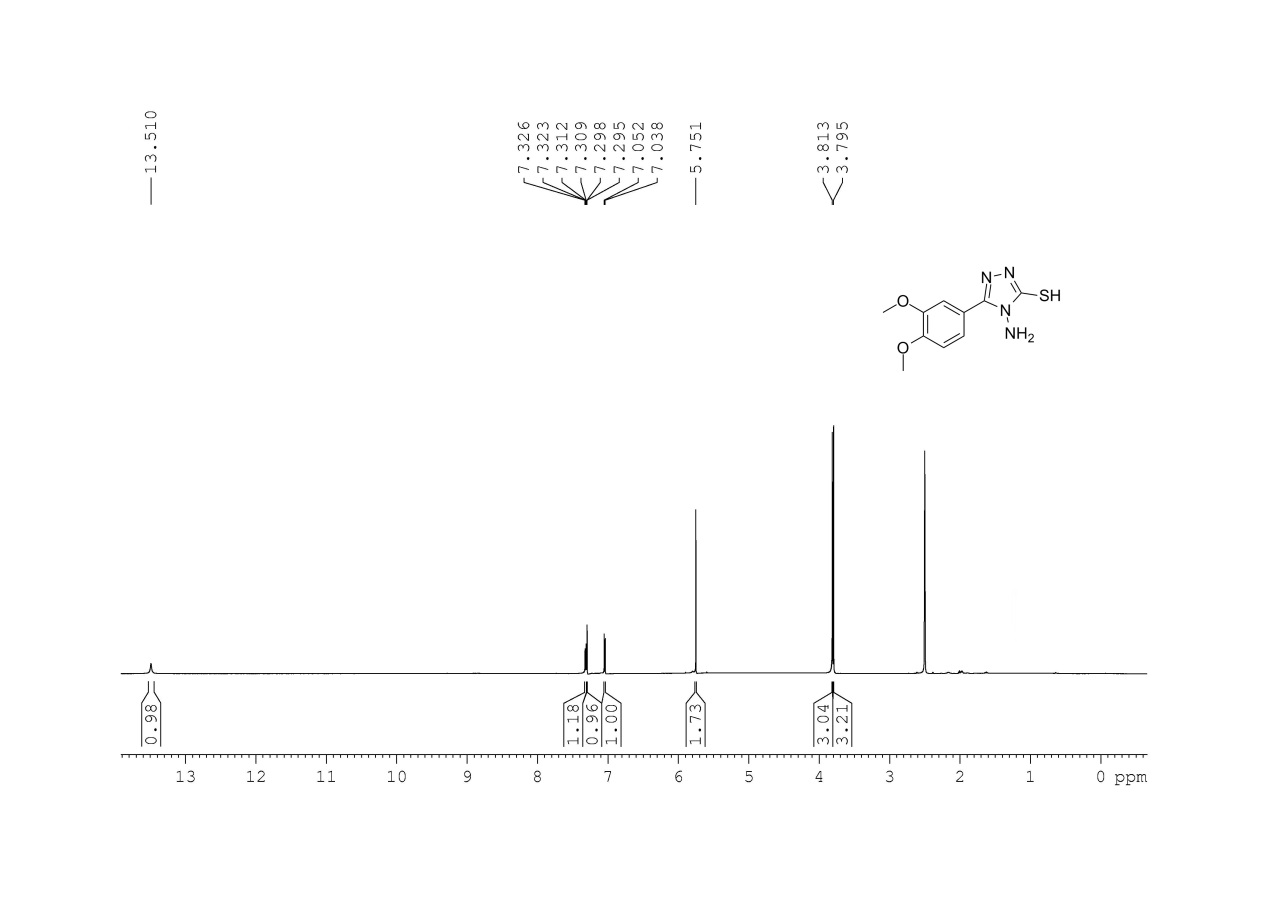


4-amino-5-(3,4,5-trimethoxyphenyl)-4*H*-1,2,4-triazole-3-thiol.
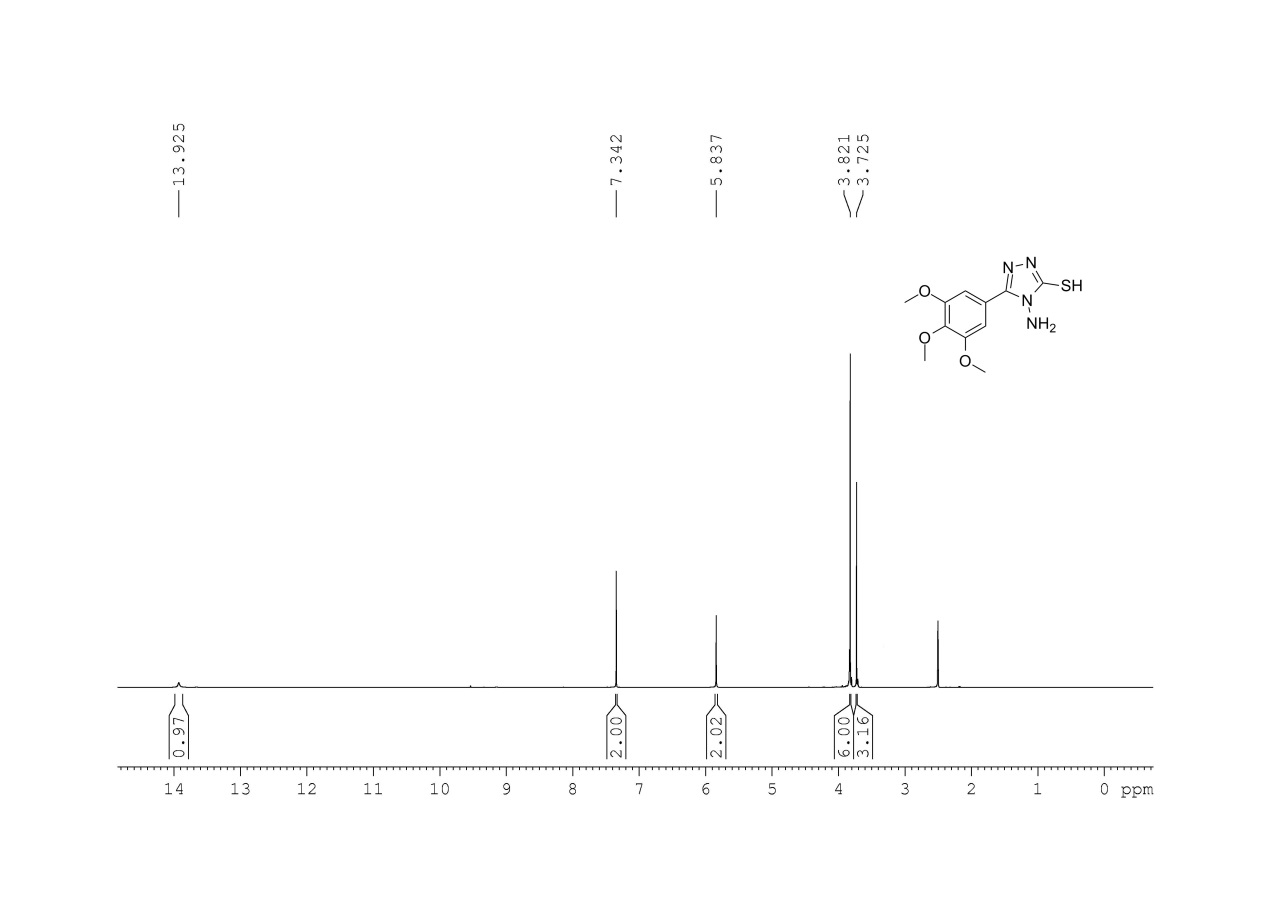


4-amino-5-(3-methoxyphenyl)-4*H*-1,2,4-triazole-3-thiol.
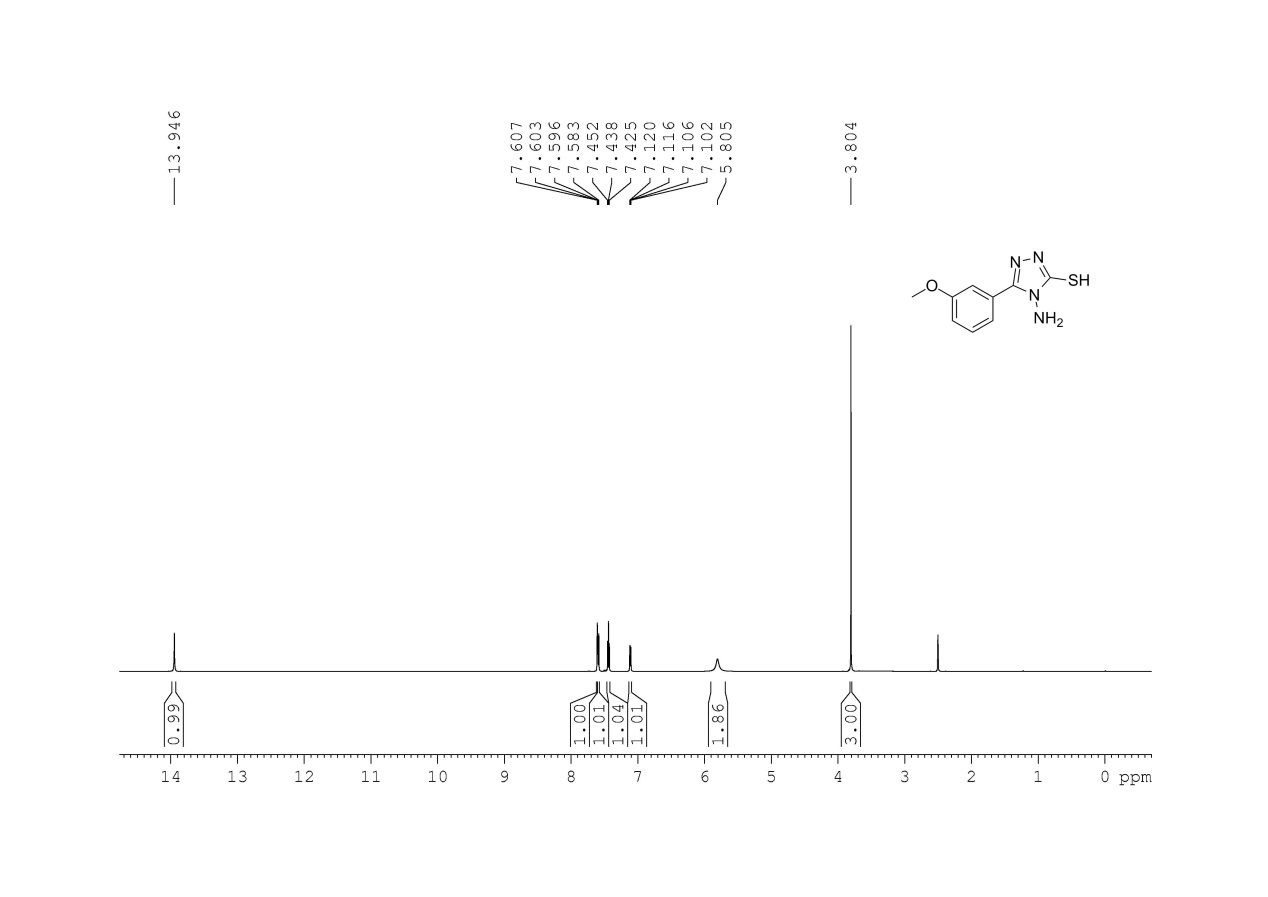


4-amino-5-(4-methoxyphenyl)-4*H*-1,2,4-triazole-3-thiol.
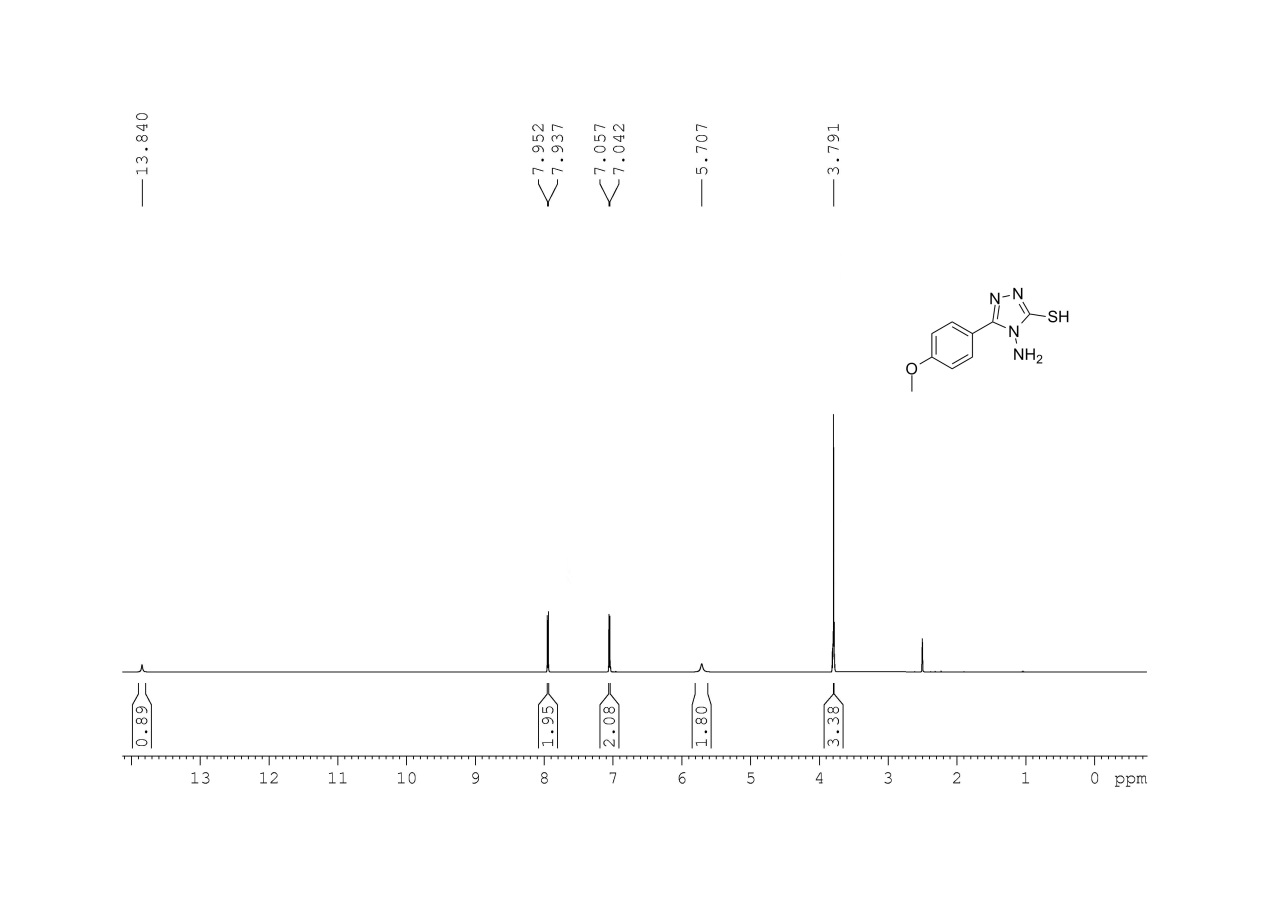


4-amino-5-(3,4-Methylenedioxyphenyl)-4*H*-1,2,4-triazole-3-thiol.
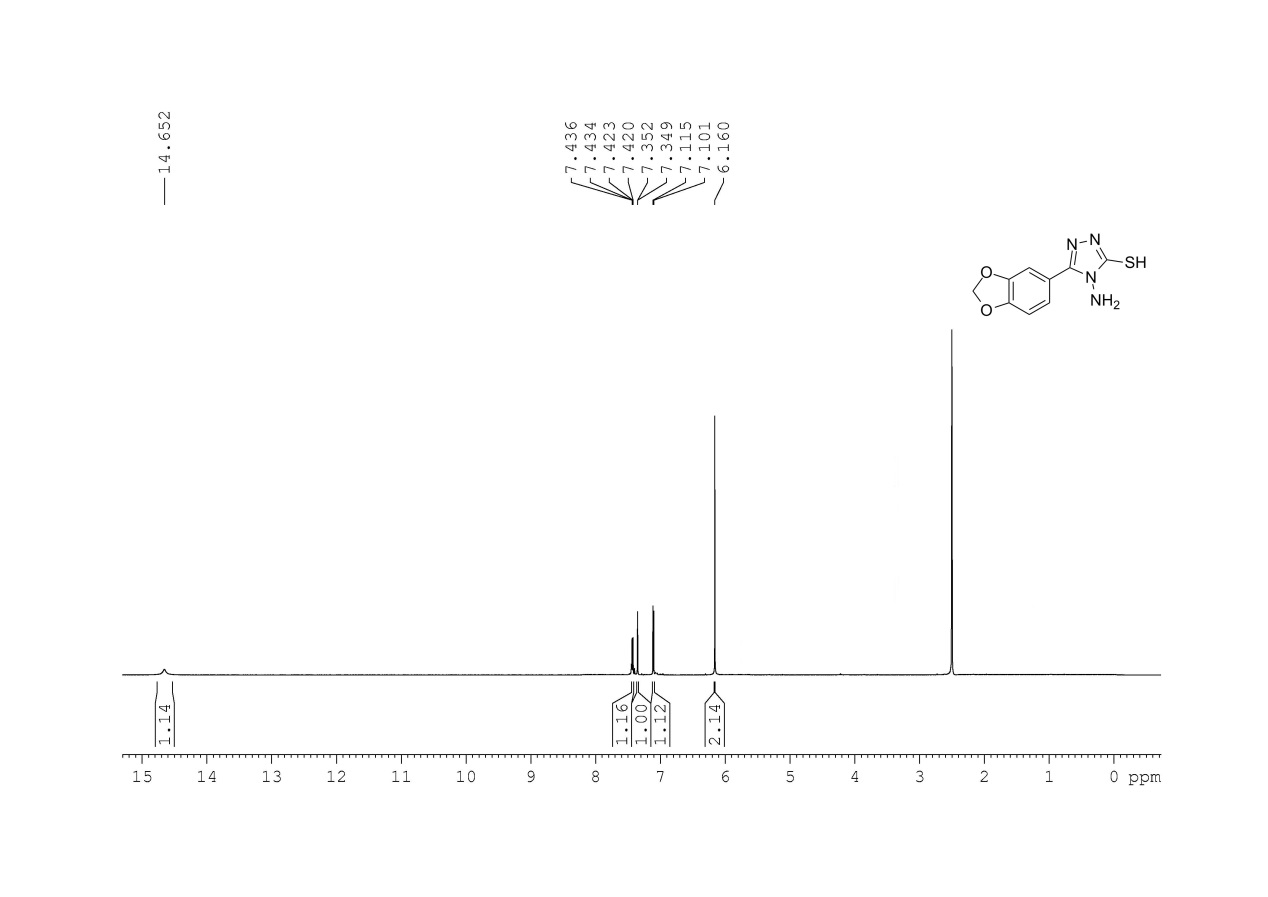


2-bromo-1-(4-fluorophenyl)ethanone.
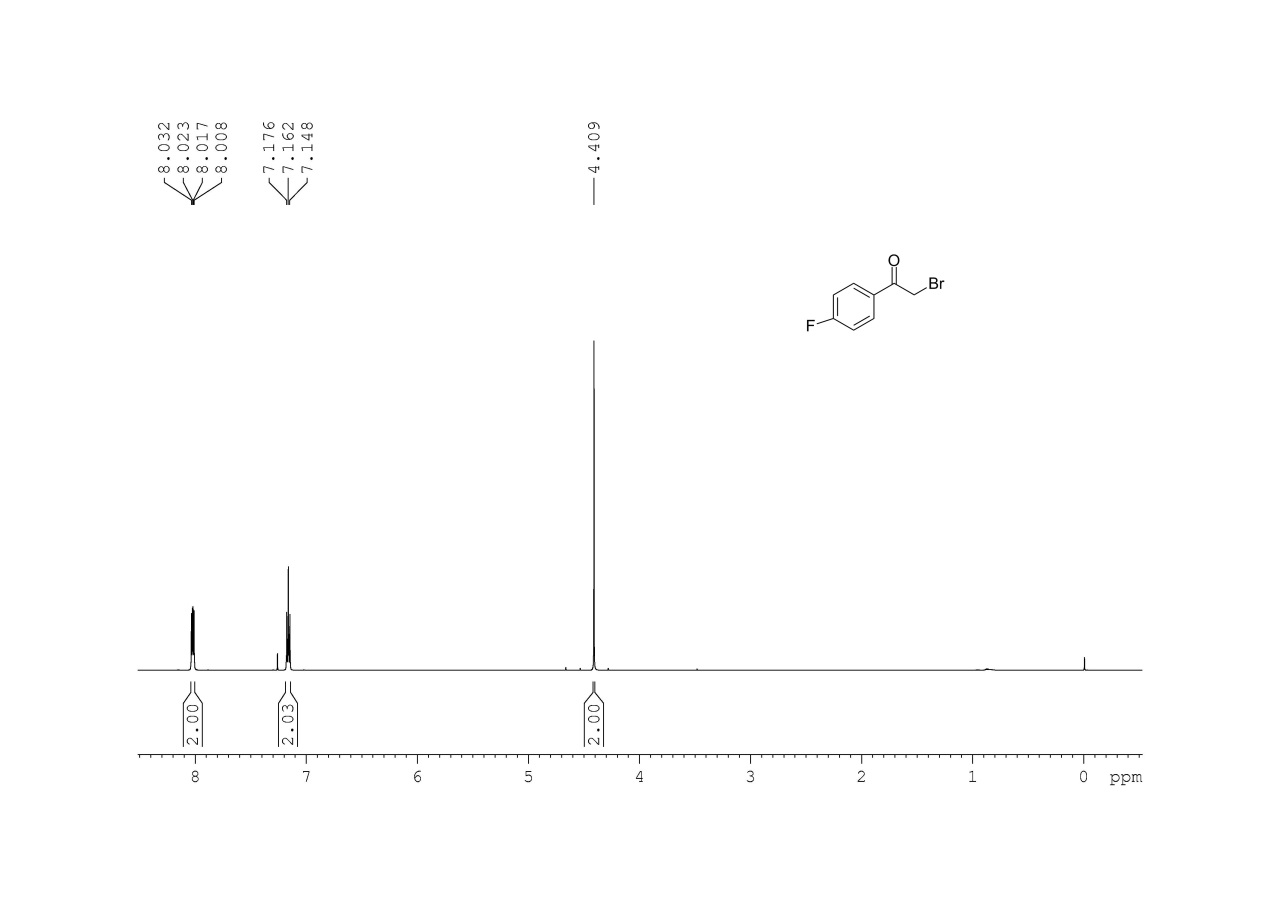


2-bromo-1-(4-chlorophenyl)ethanone.
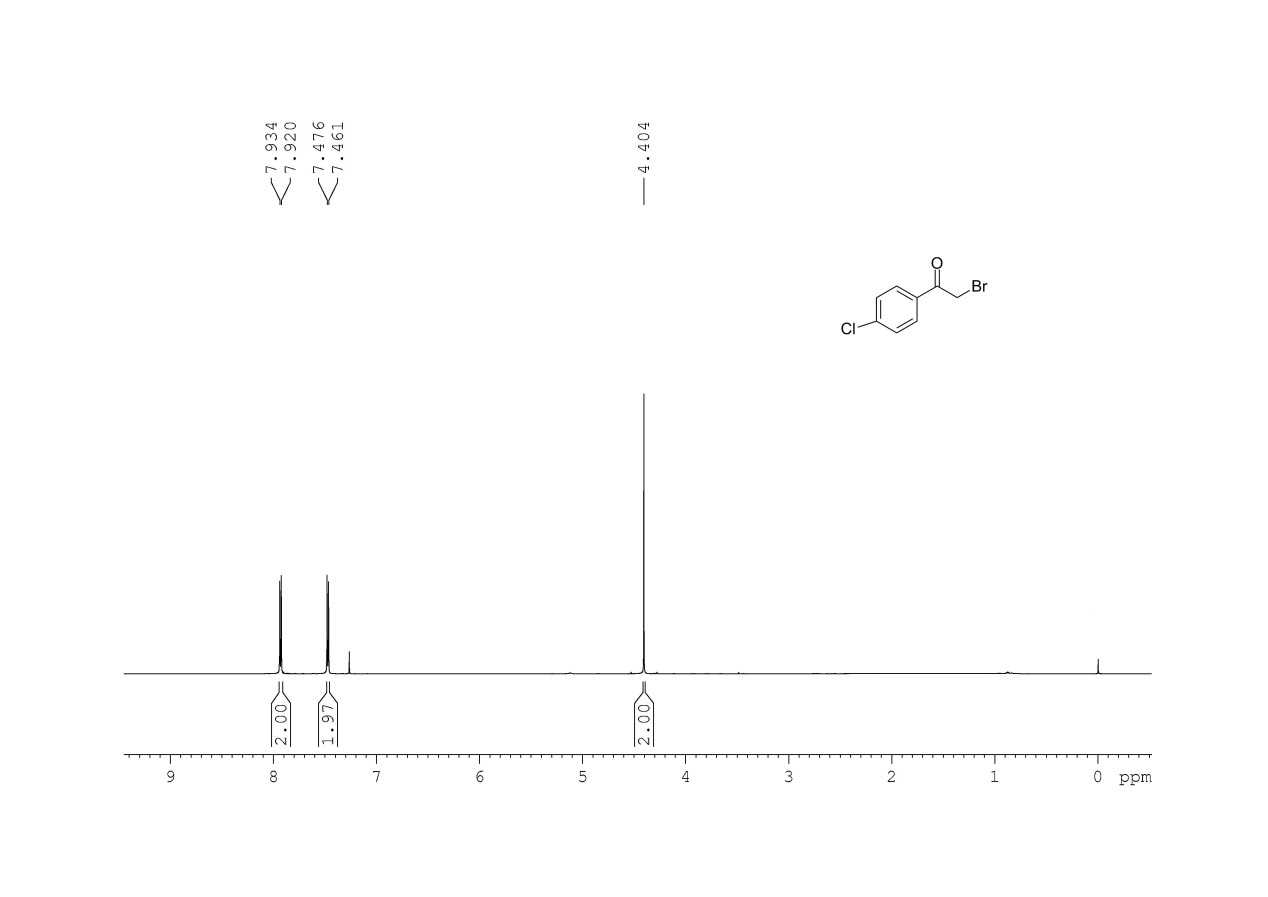


2-bromo-1-(4-bromophenyl)ethanone.
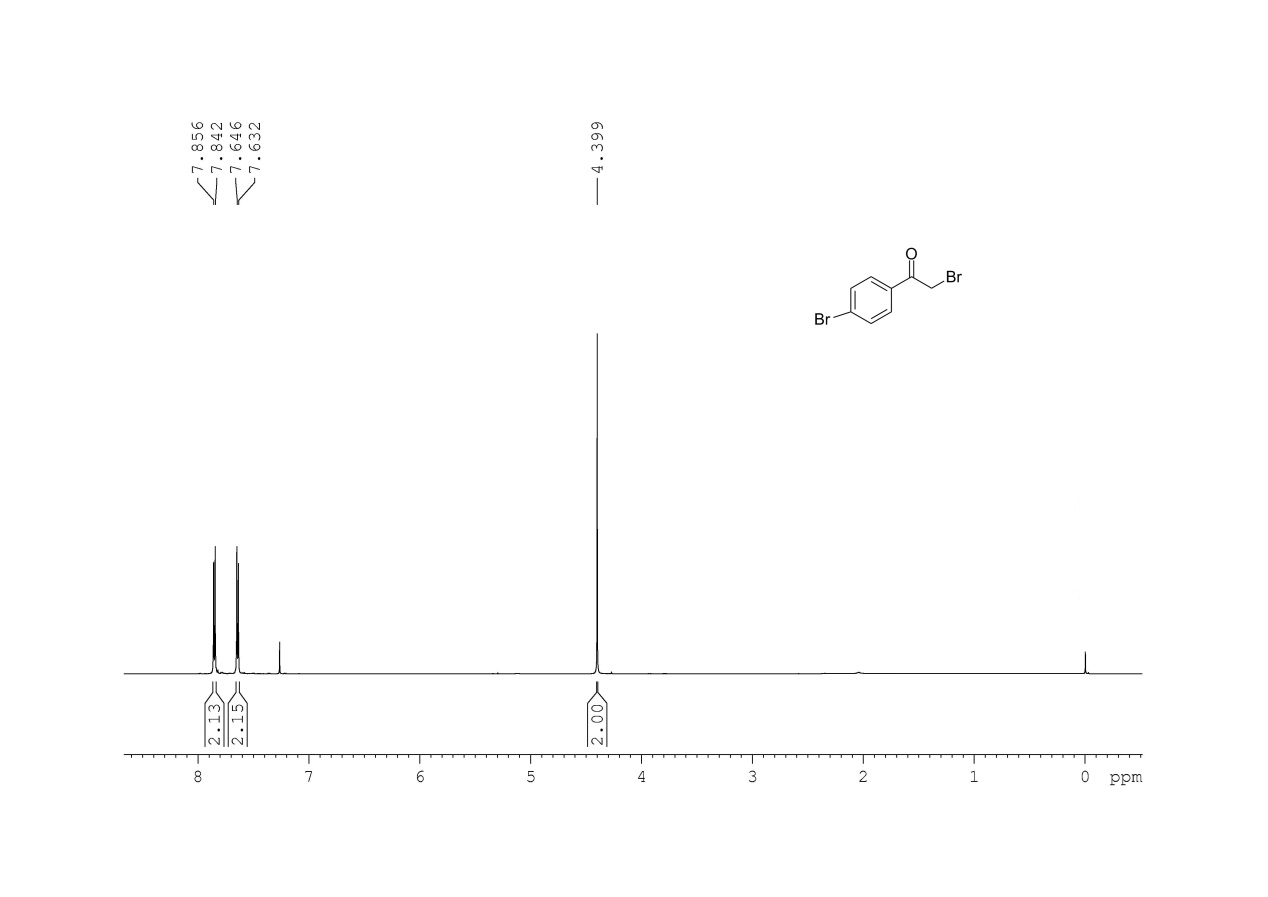


2-bromo-1-(*p*-tolyl)ethanone.
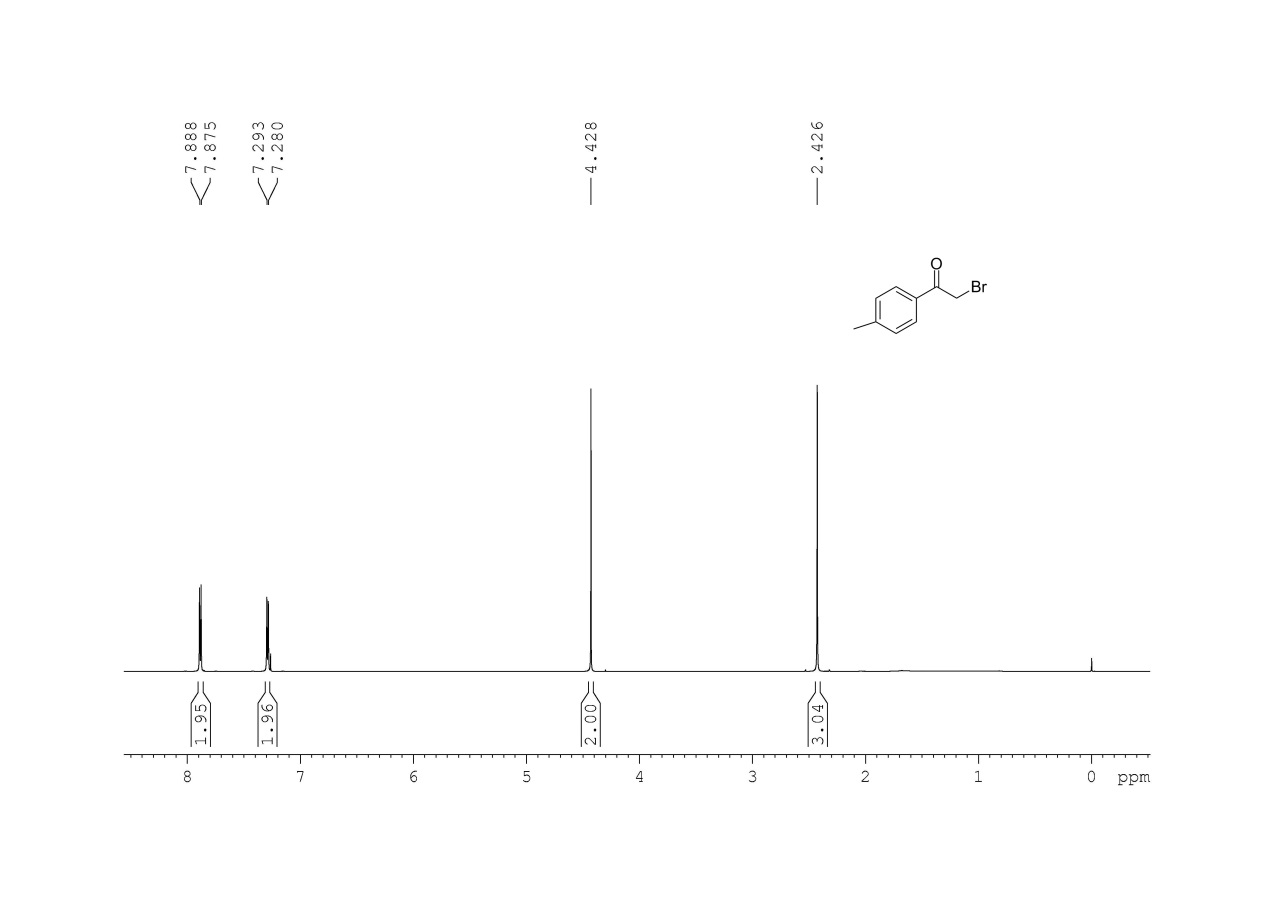


2-bromo-1-(4-(trifluoromethyl)phenyl)ethanone.
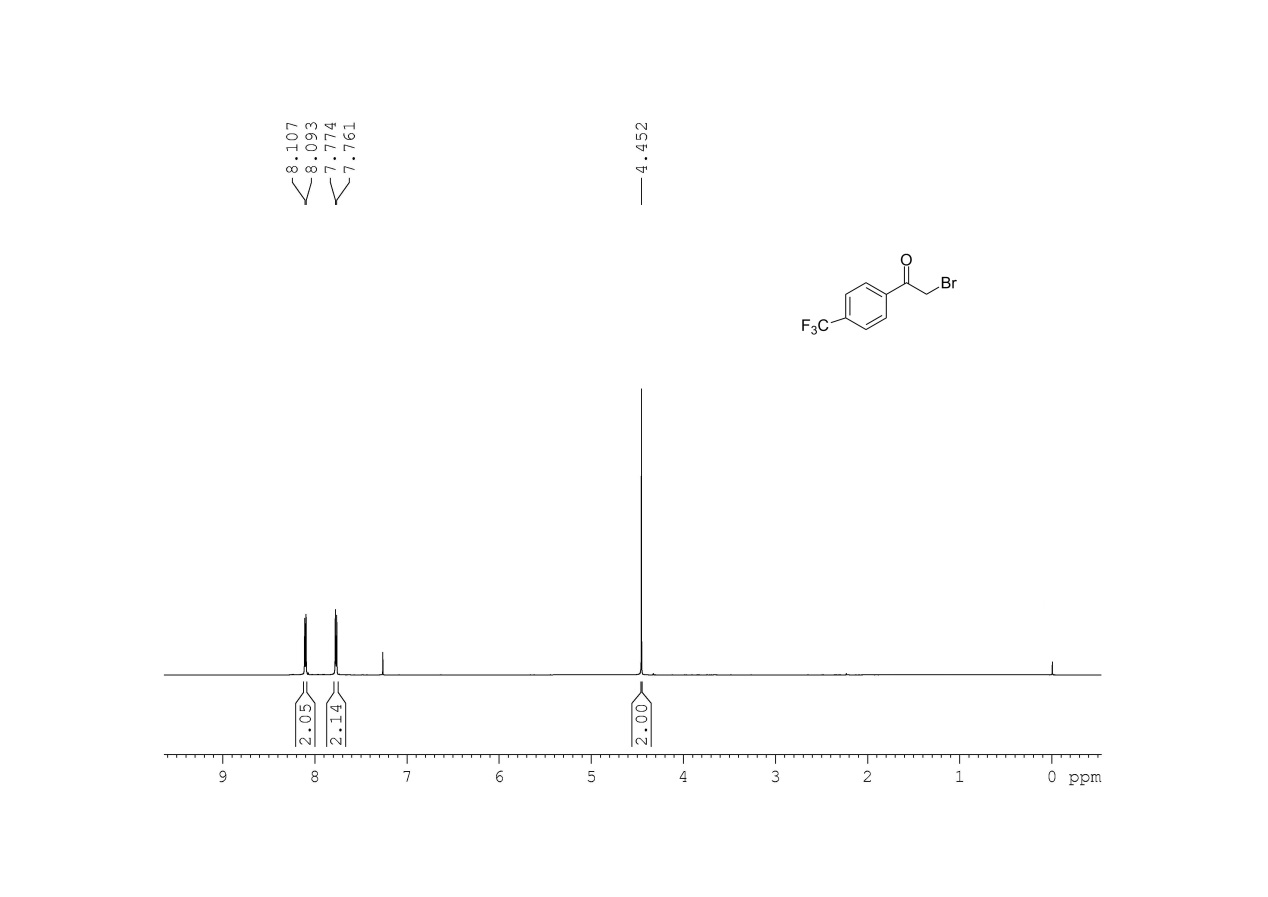


2-bromo-1-(4-methoxyphenyl)ethanone.
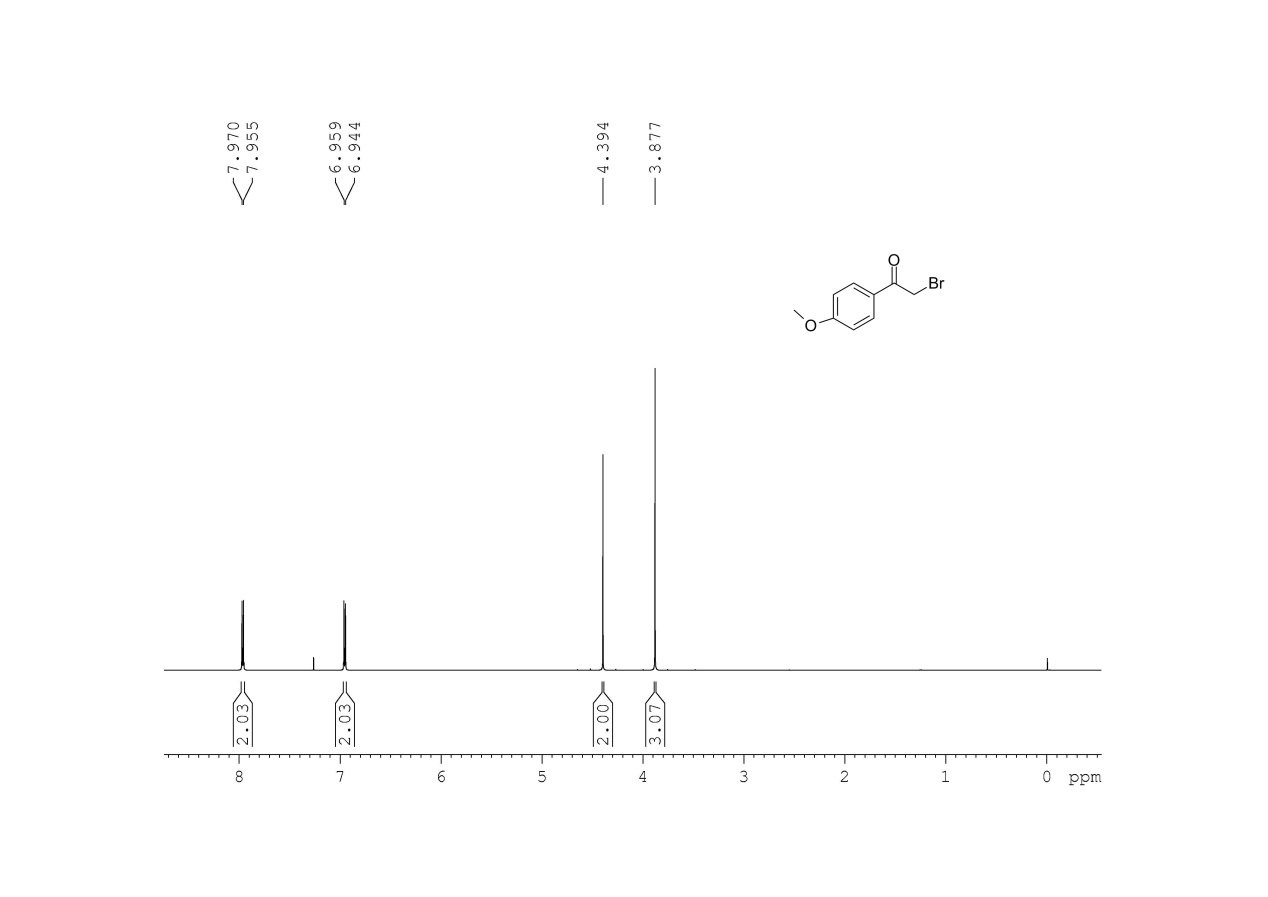


2-bromo-1-(4-(methylthio)phenyl)ethanone.
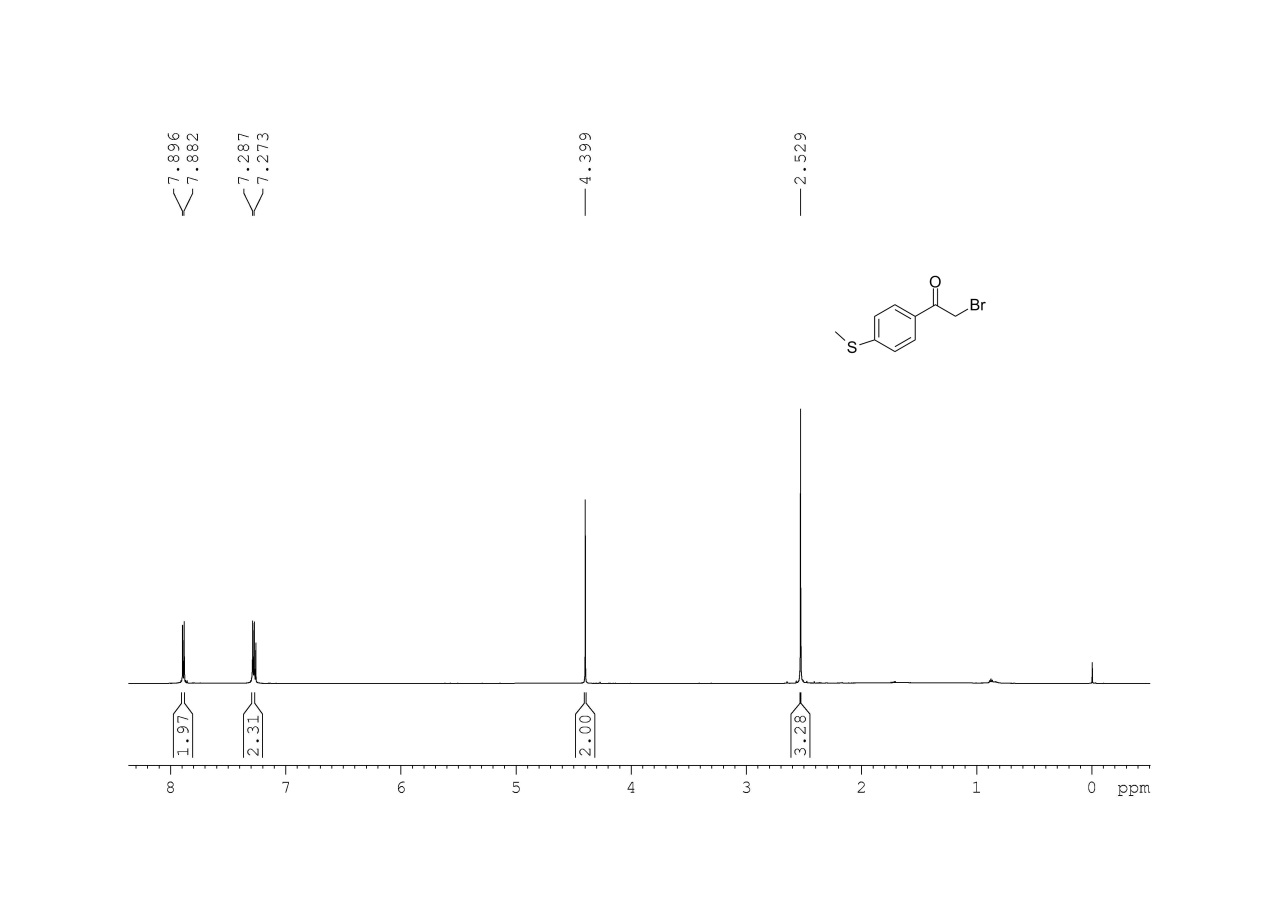


2-bromo-1-(3-fluoro-4-methoxyphenyl)ethanone.
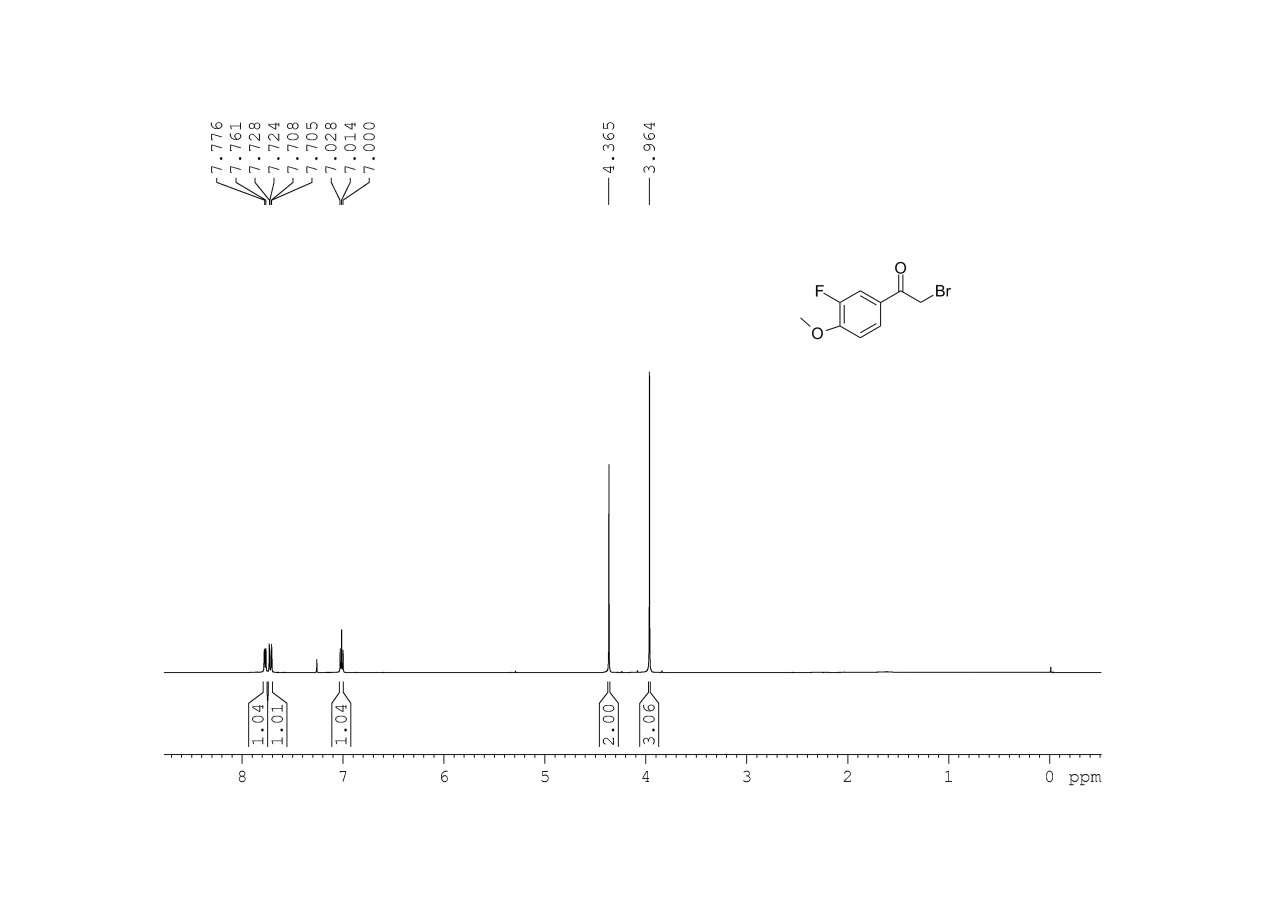


2-bromo-1-(4-methoxy-3-nitrophenyl)ethanone.
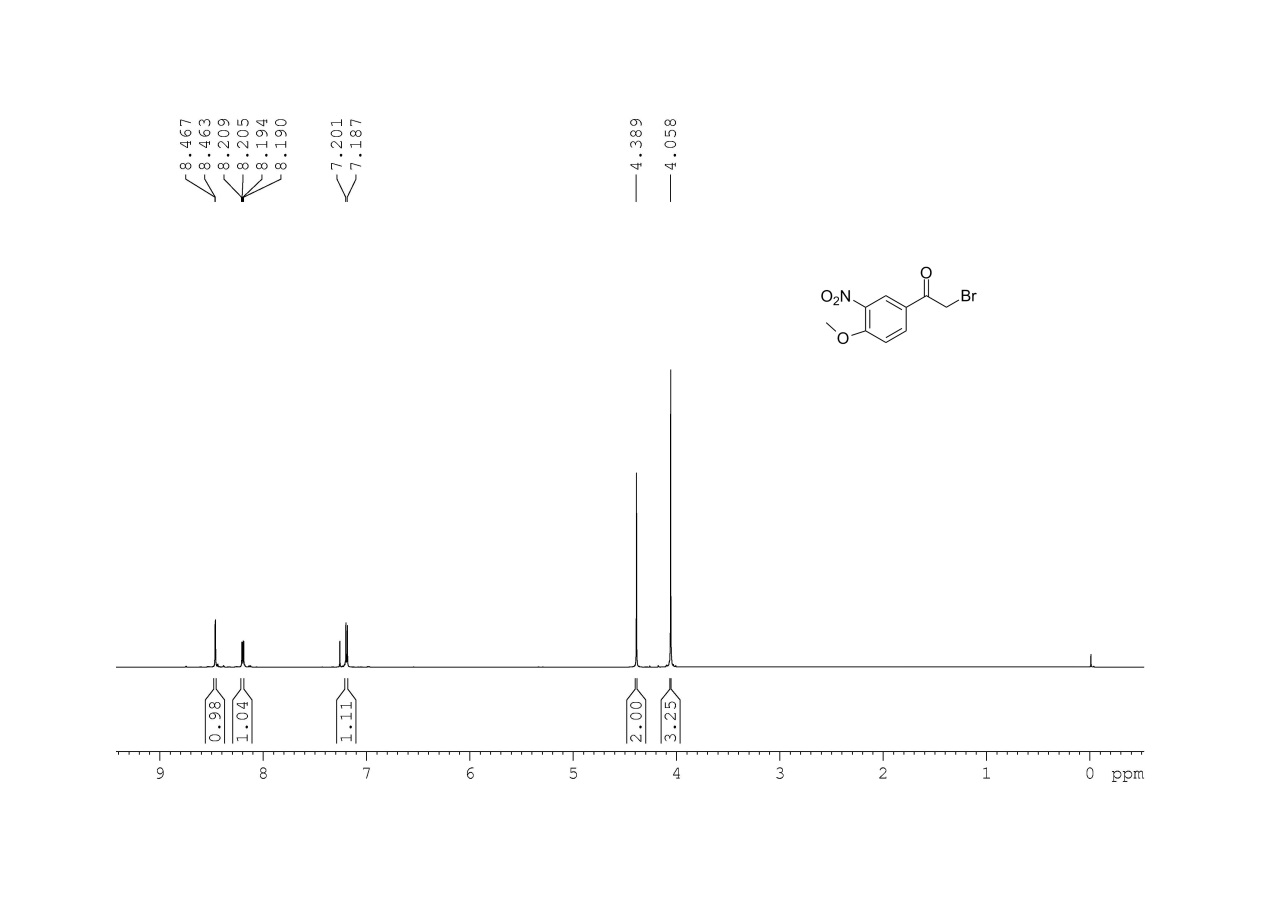


2-bromo-1-(3-(benzyloxy)-4-methoxyphenyl)ethanone.
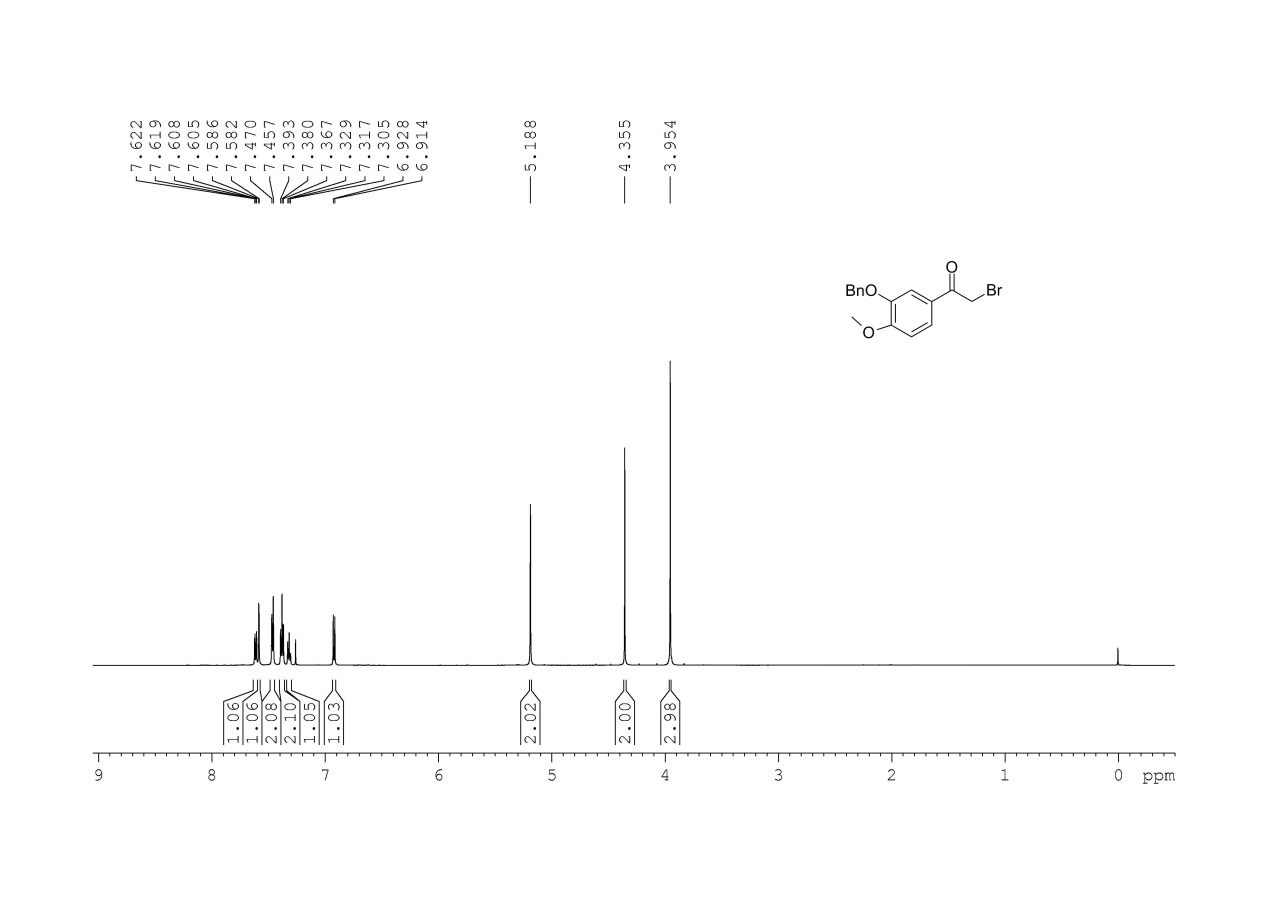


2-bromo-1-(3,4-difluorophenyl)ethanone.
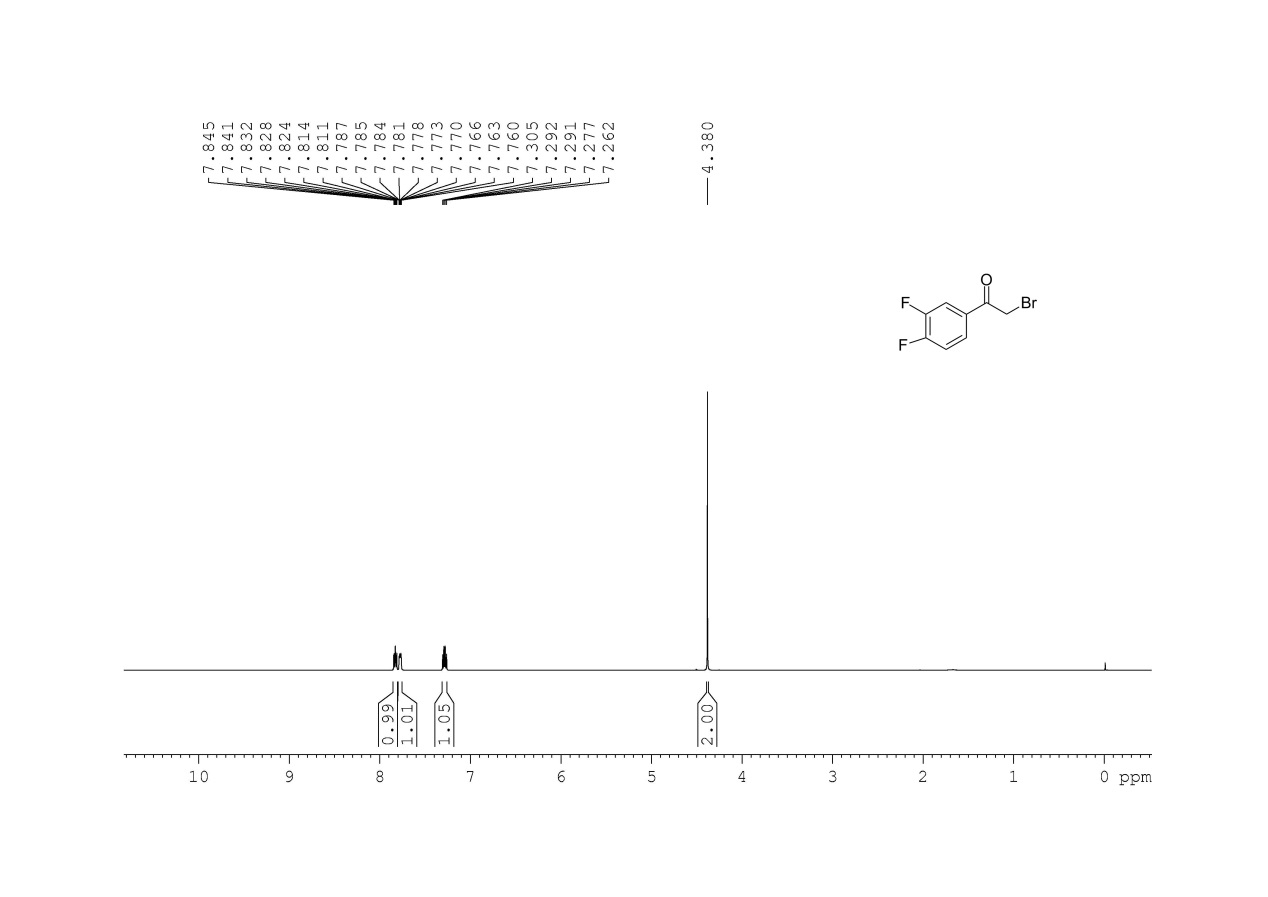


**(2). ^1^H-NMR and ^13^C-NMR spectra of all target compounds.**

3-(2,3,4-Trimethoxyphenyl)-6-(4-fluorophenyl)-*7H*-[1,2,4]triazolo[3,4-b][1,3,4]thiadiazine (**4a)**.


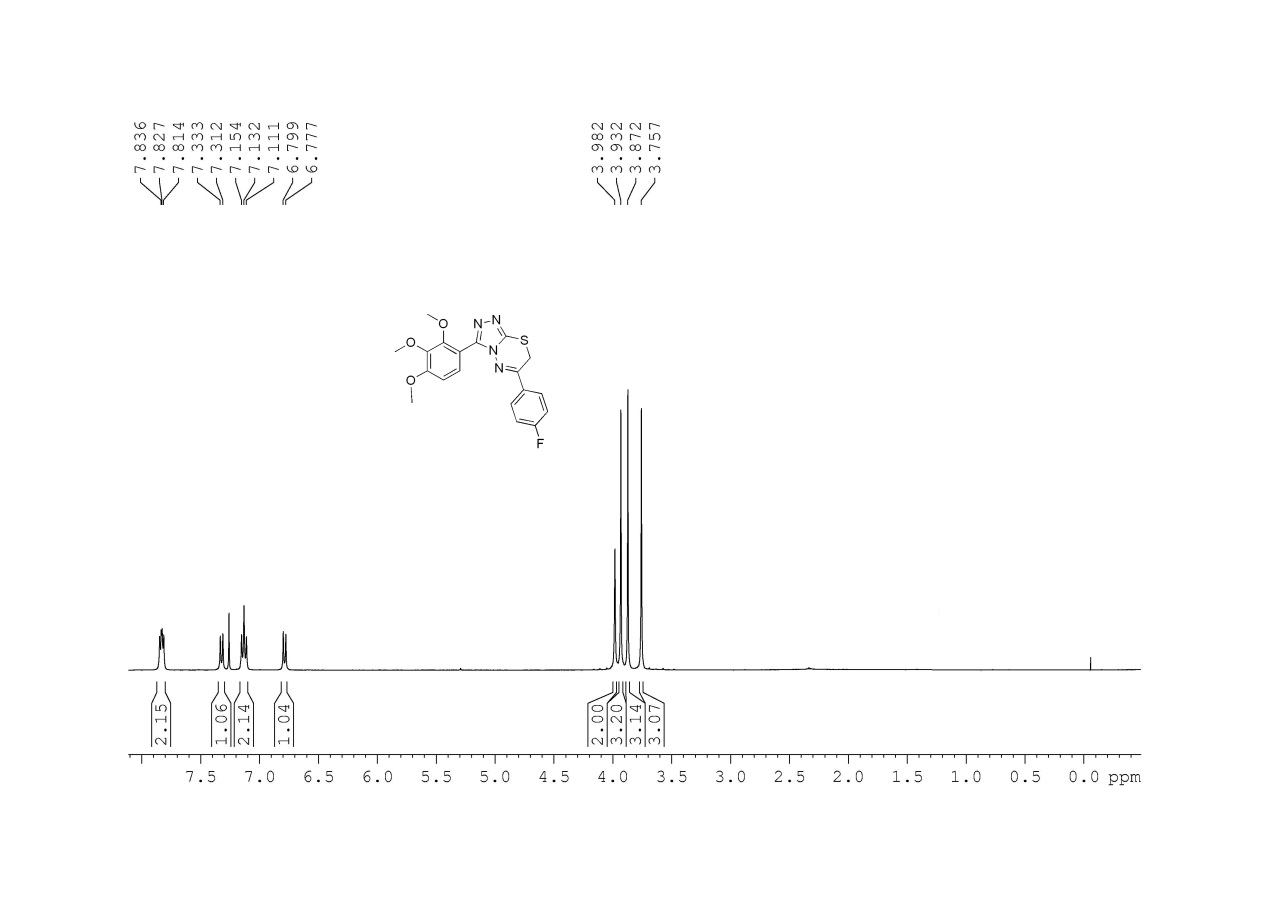

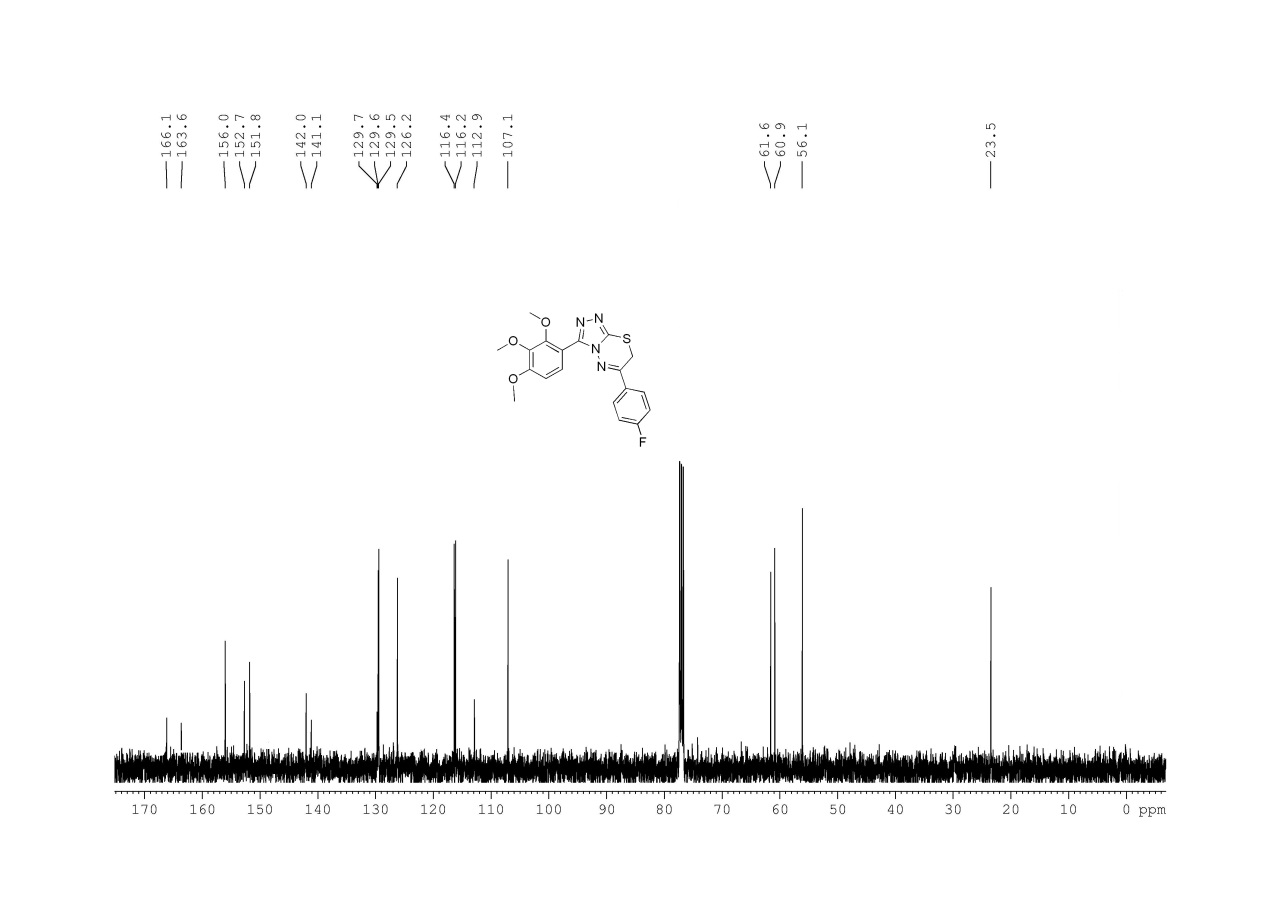


3-(2,3,4-Trimethoxyphenyl)-6-(4-chlorophenyl)-*7H*-[1,2,4]triazolo[3,4-b][1,3,4]thiadiazine (**4b**).


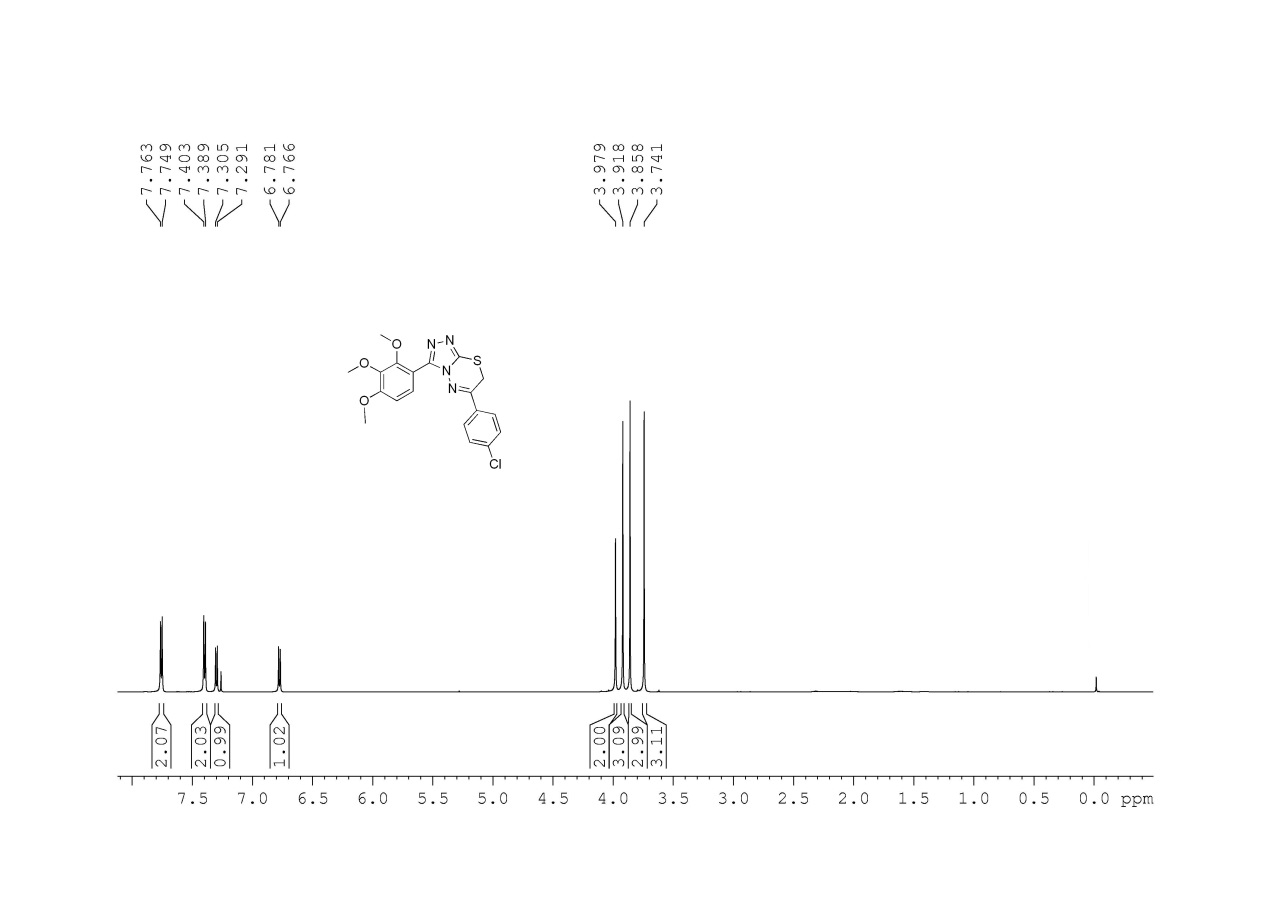

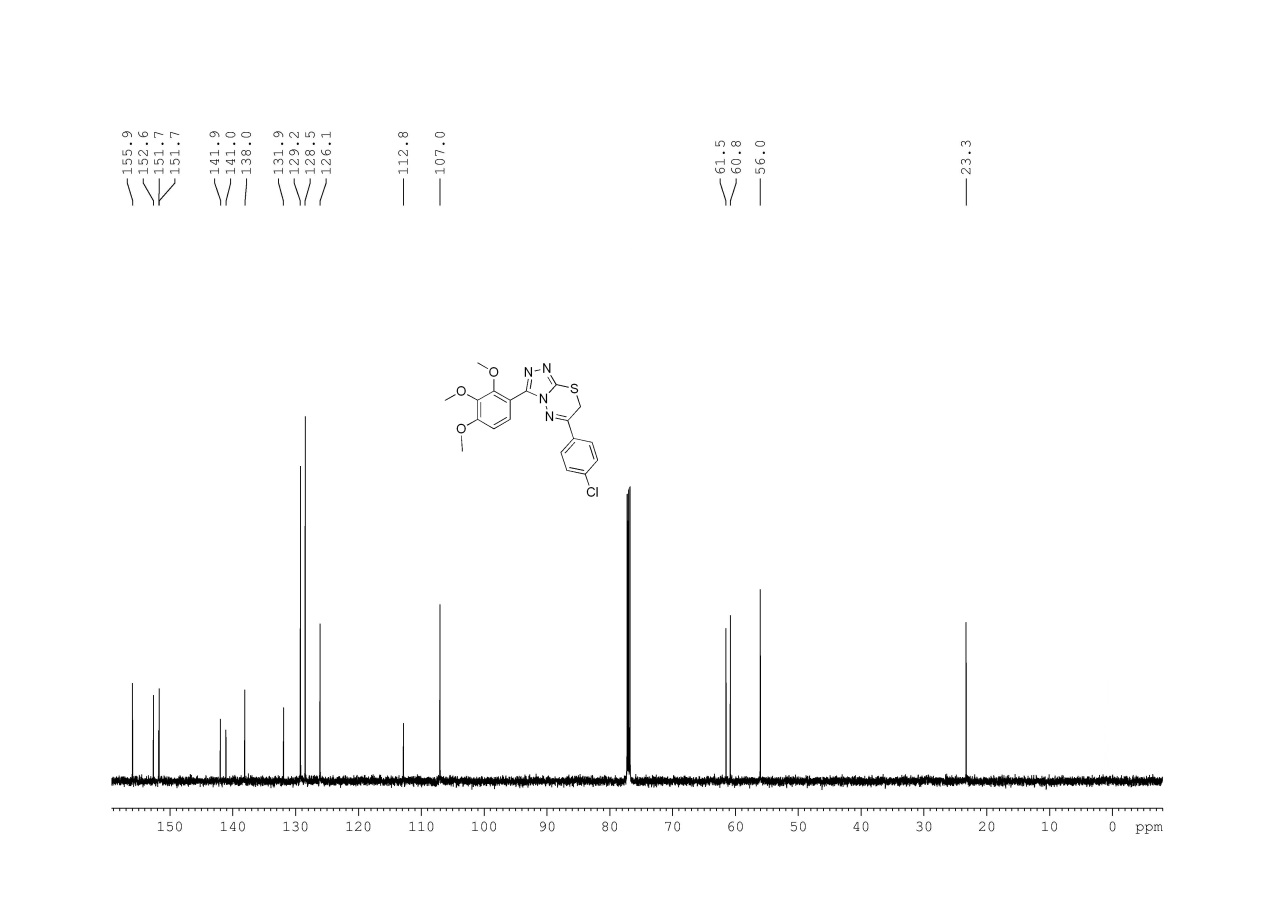


3-(2,3,4-Trimethoxyphenyl)-6-(4-bromophenyl)-*7H*-[1,2,4]triazolo[3,4-b][1,3,4]thiadiazine (**4c**).


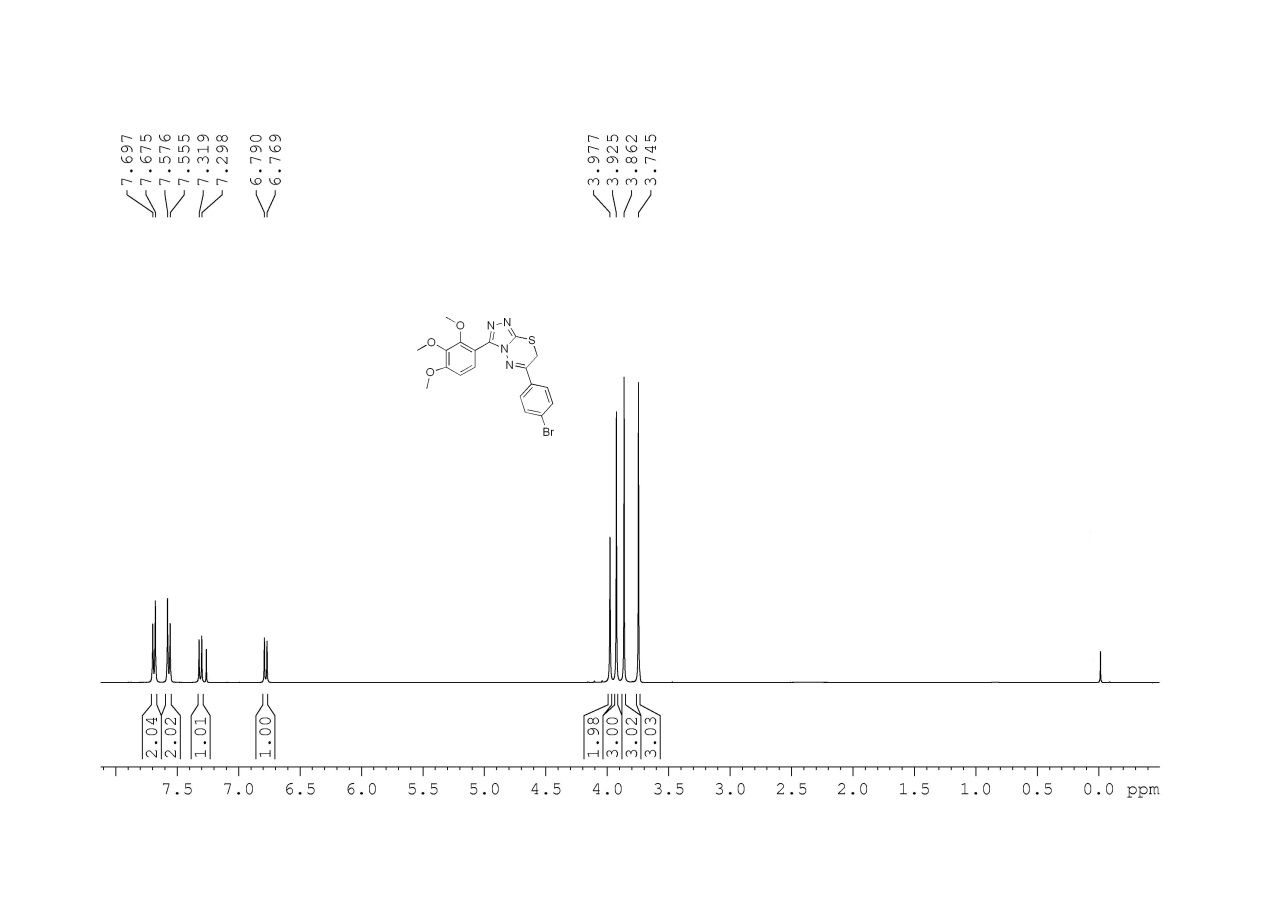

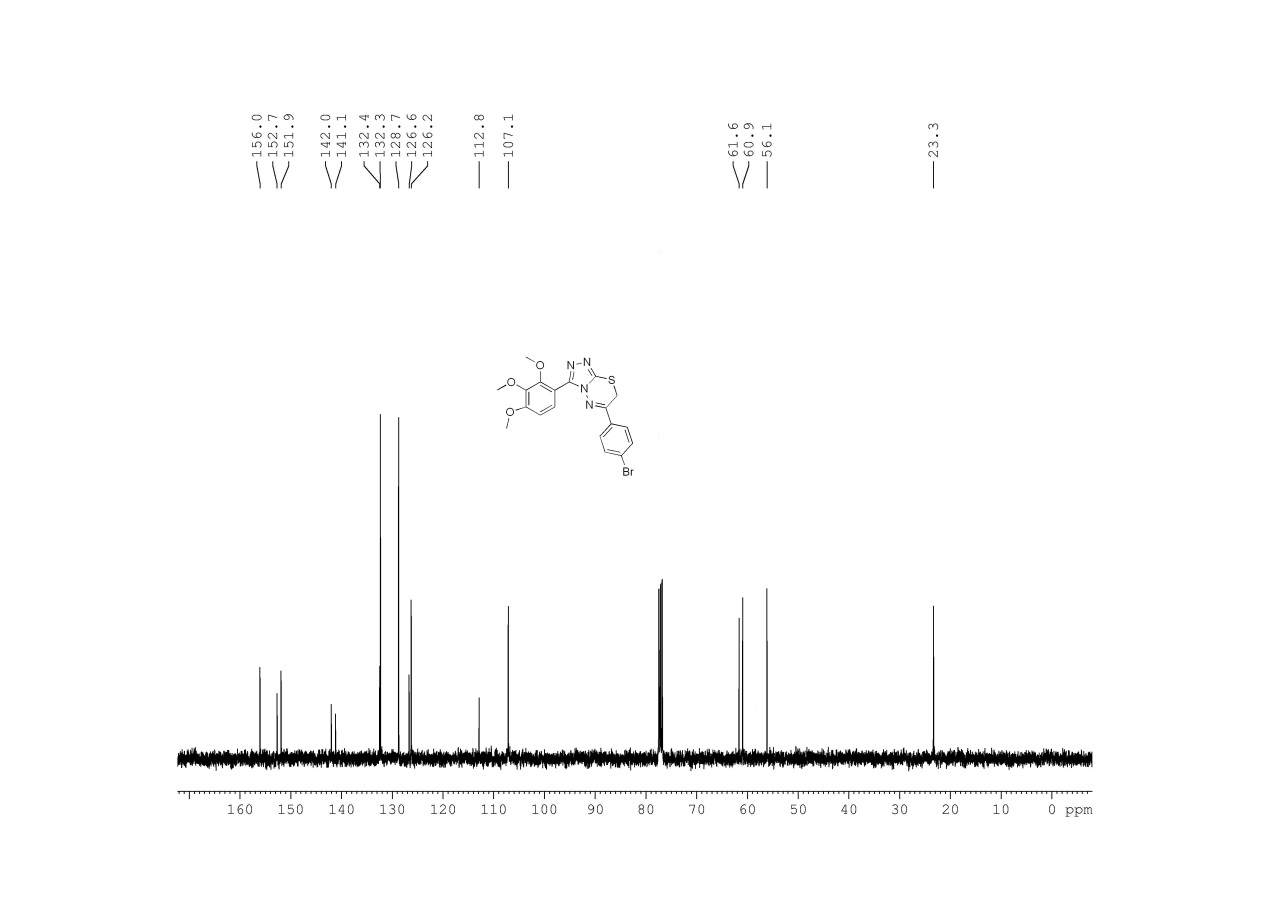


3-(2,3,4-Trimethoxyphenyl)-6-(4-methyphenyl)-*7H*-[1,2,4]triazolo[3,4-b][1,3,4]thiadiazine (**4d**).


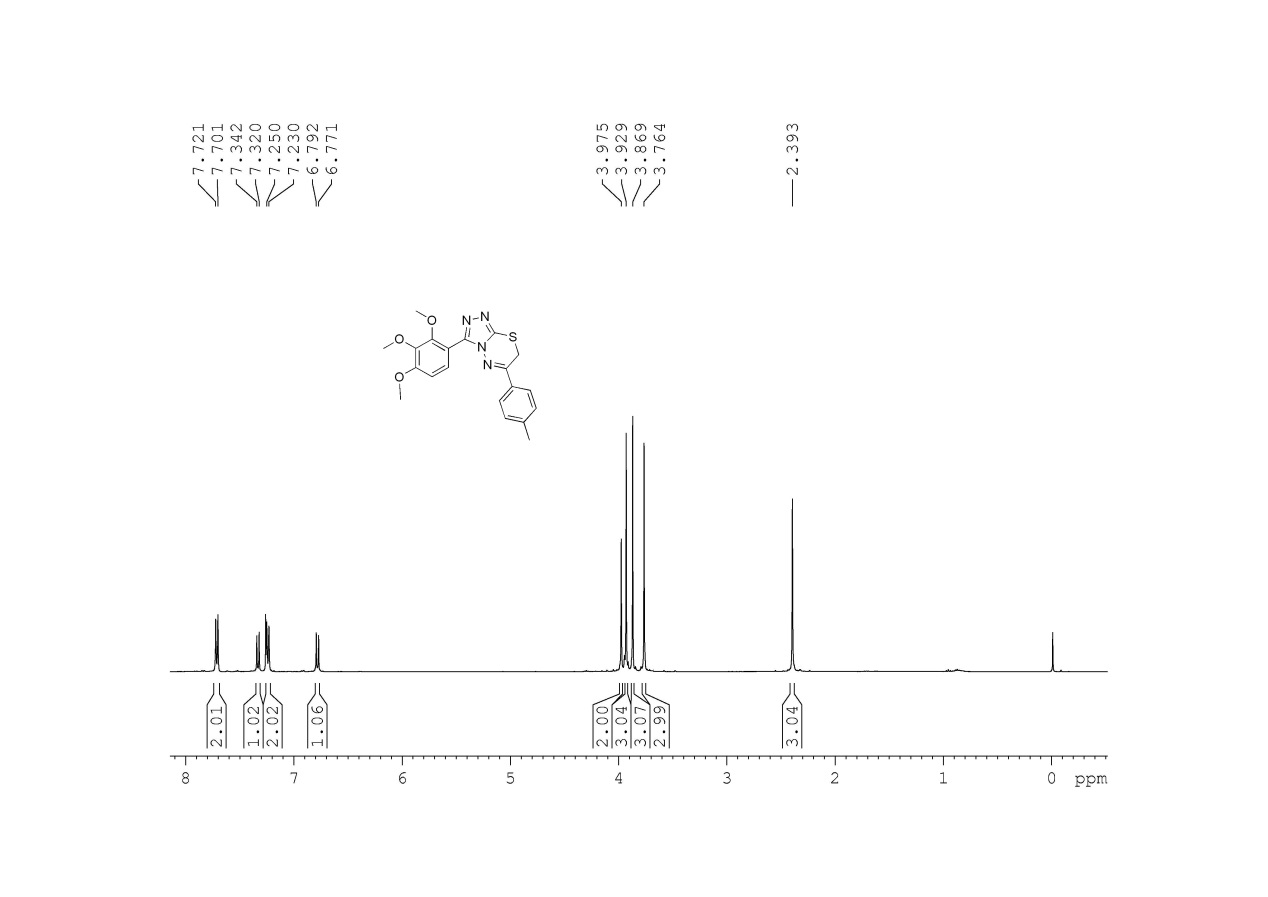

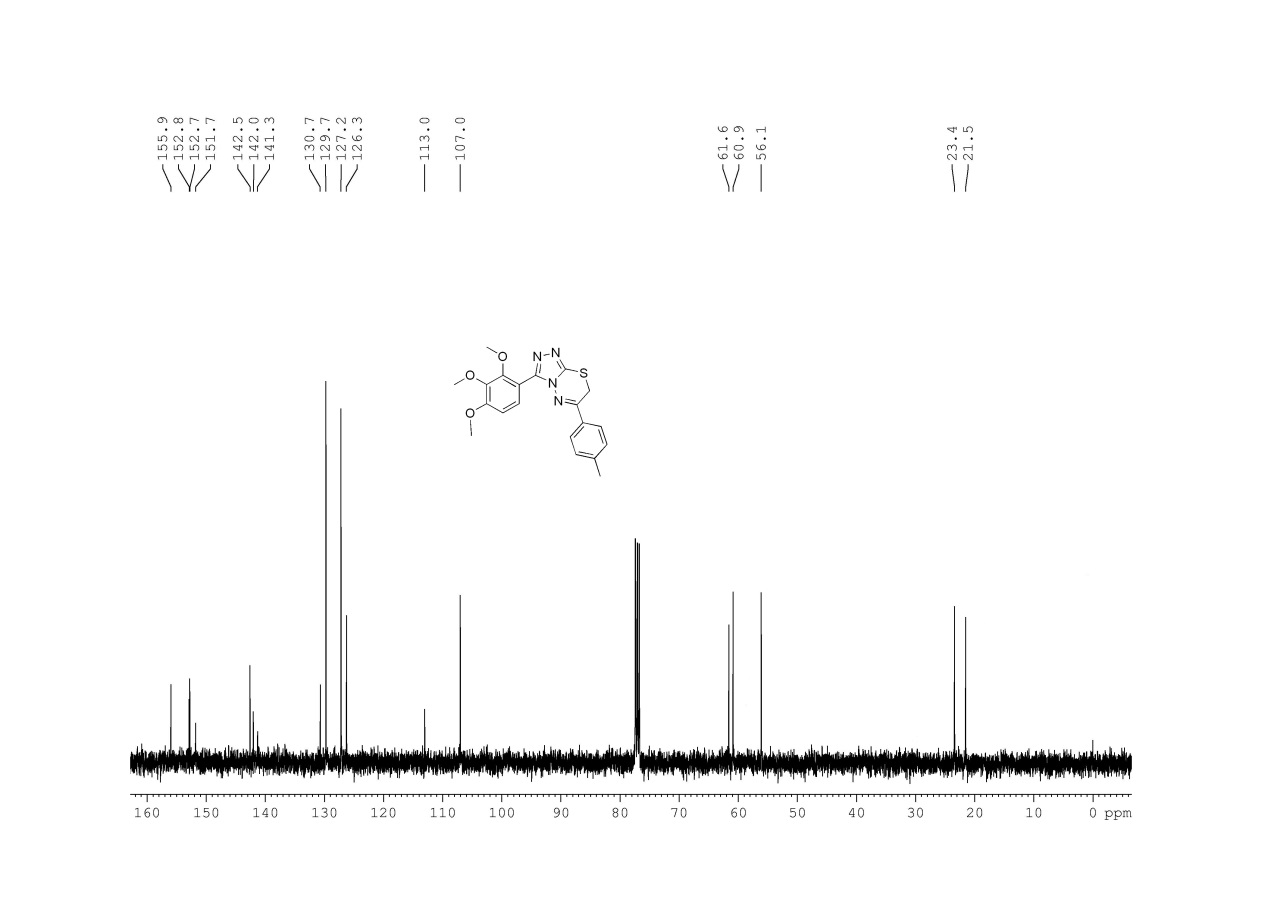


3-(2,3,4-Trimethoxyphenyl)-6-(4-(trifluoromethyl)phenyl)-*7H*-[1,2,4]triazolo[3,4-b][1,3,4]thiadiazine (**4e**).


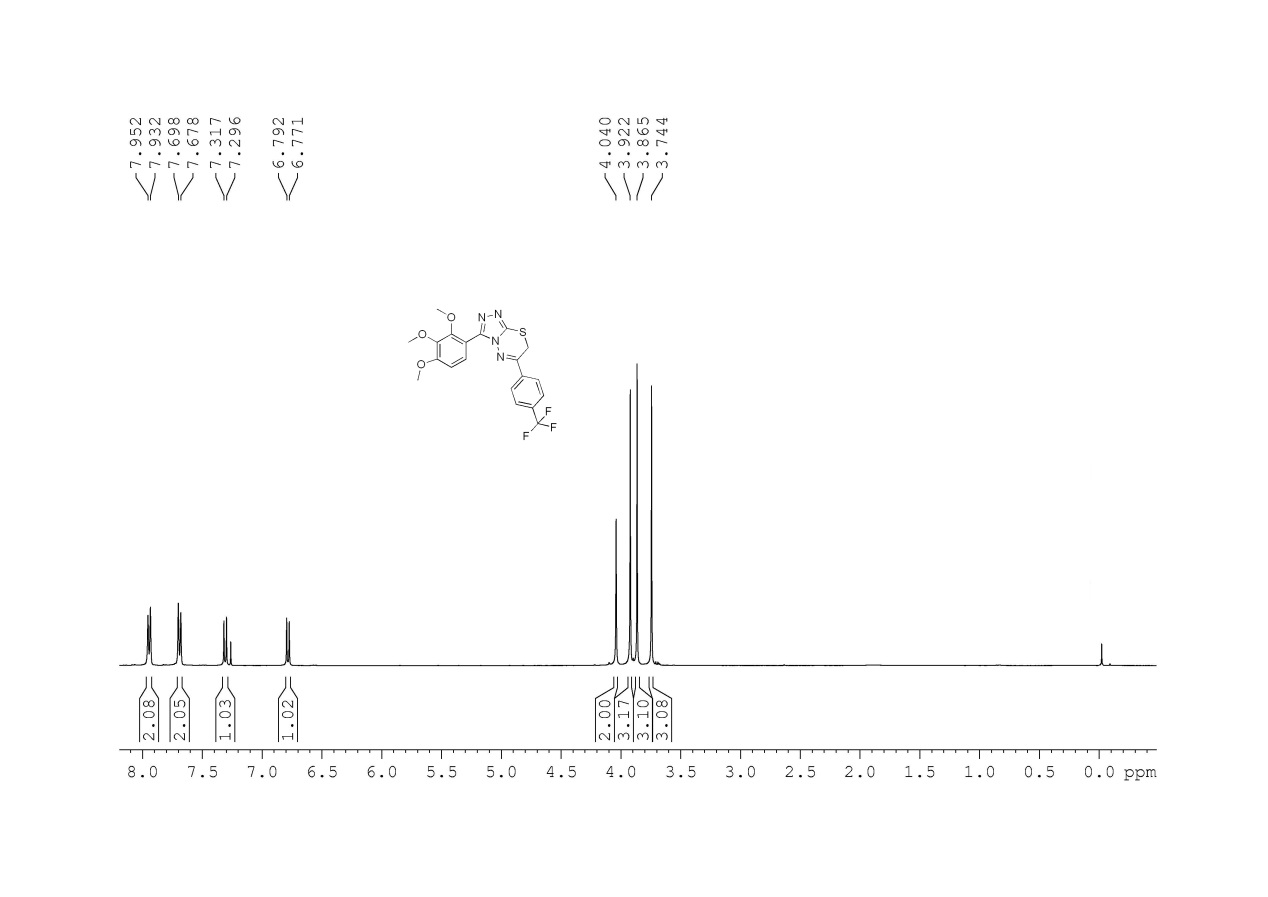

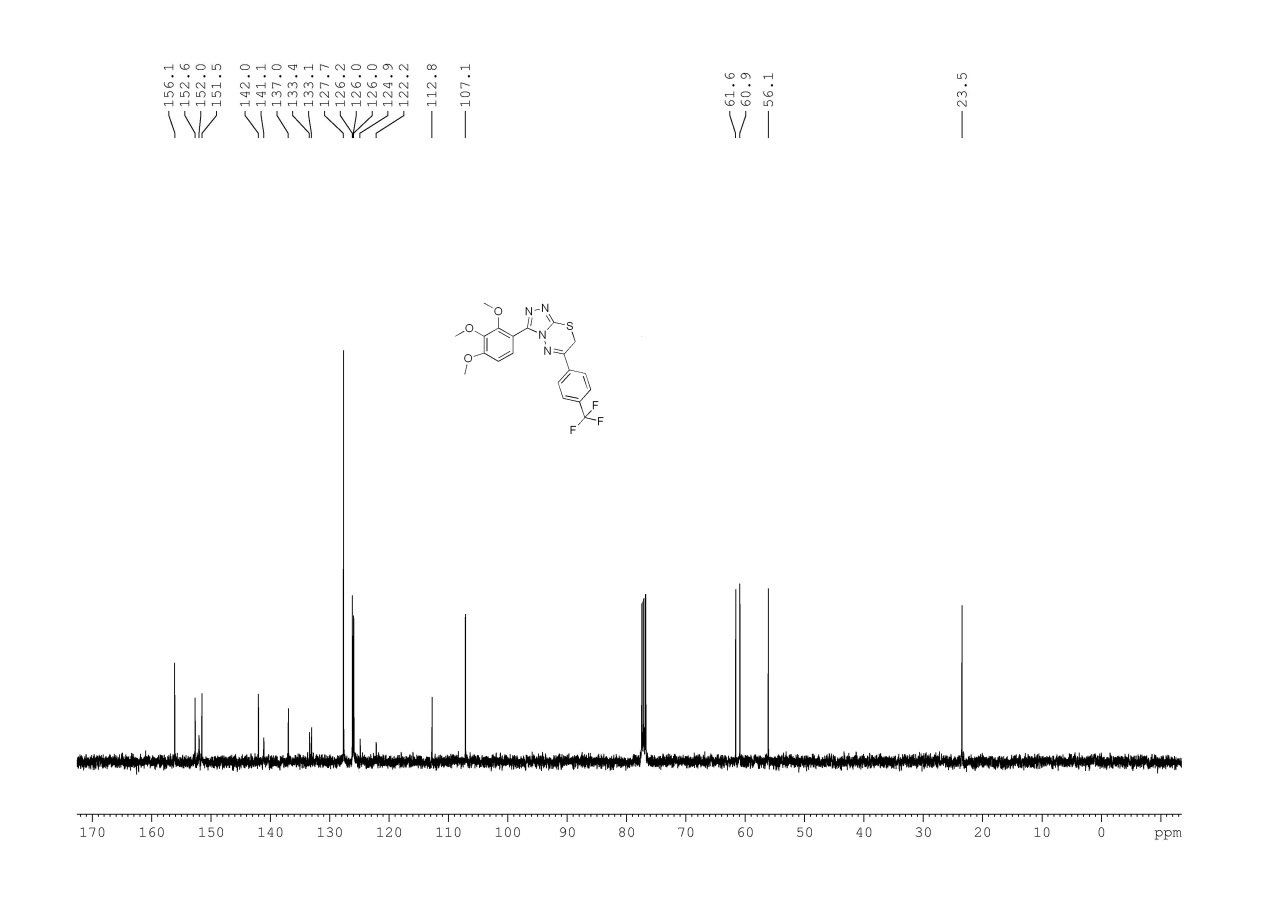


3-(2,3,4-Trimethoxyphenyl)-6-(4-methoxyphenyl)-*7H*-[1,2,4]triazolo[3,4-b][1,3,4]thiadiazine (**4f**).


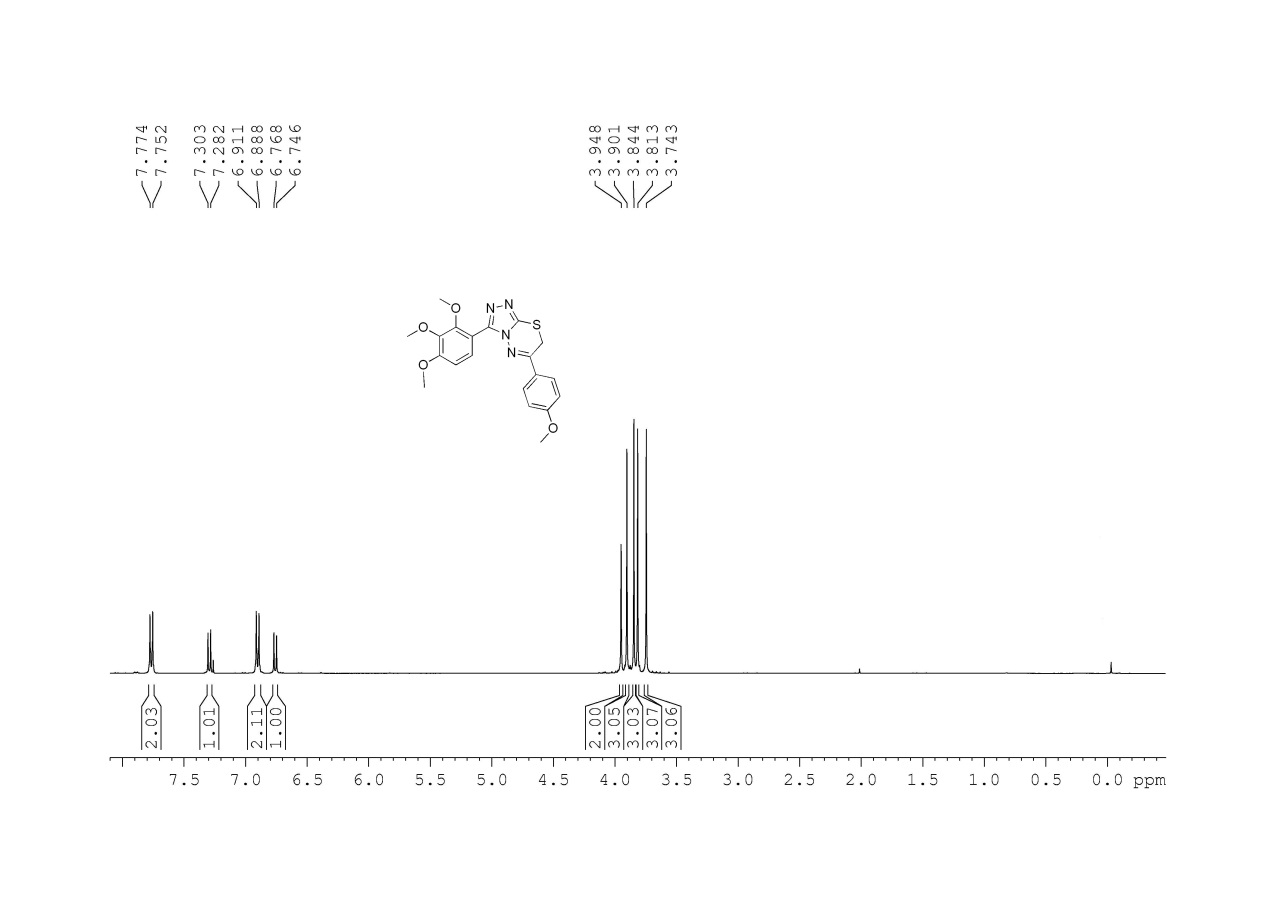


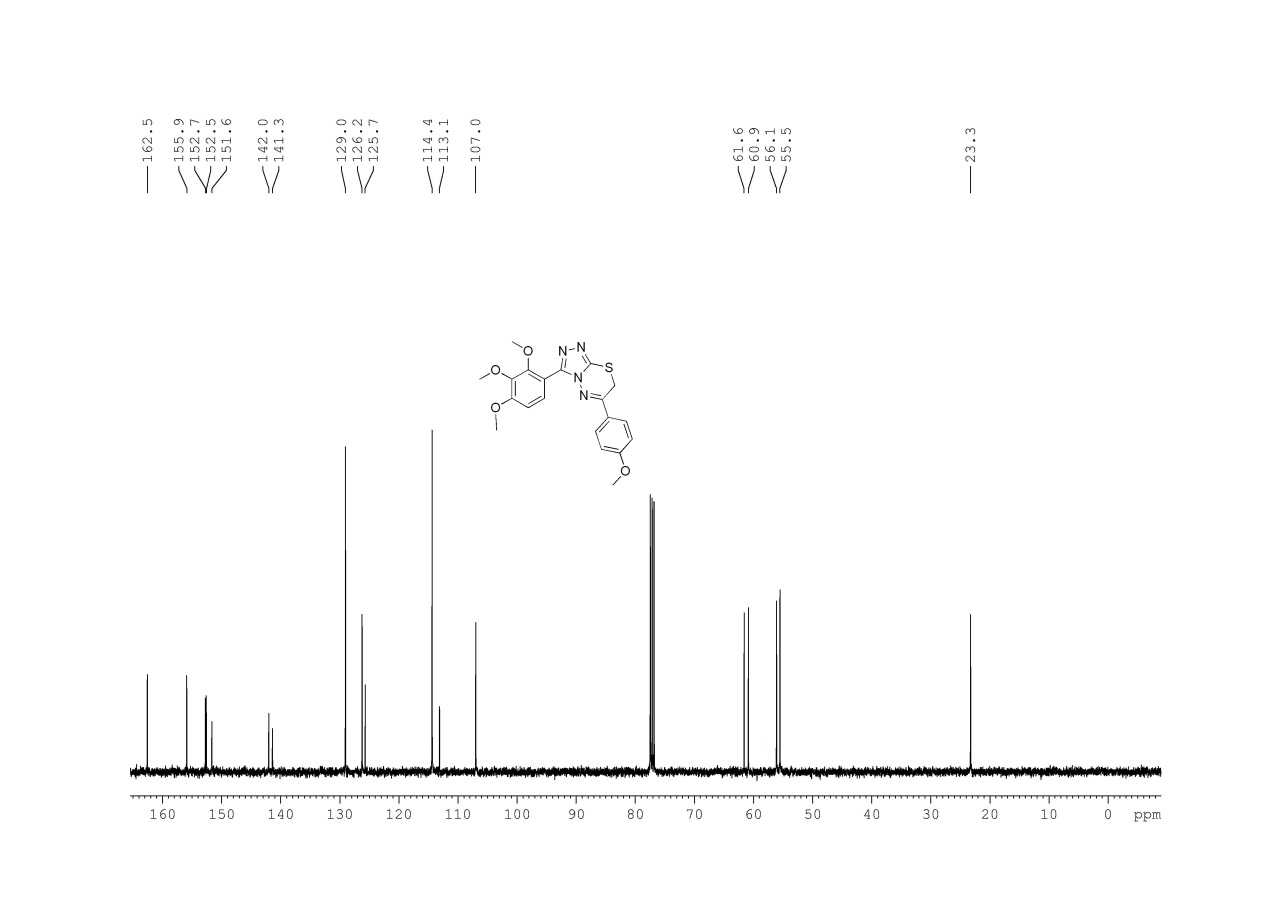


3-(2,3,4-Trimethoxyphenyl)-6-(4-methylthiophenyl)-*7H*-[1,2,4]triazolo[3,4-b][1,3,4]thiadiazine (**4g**).


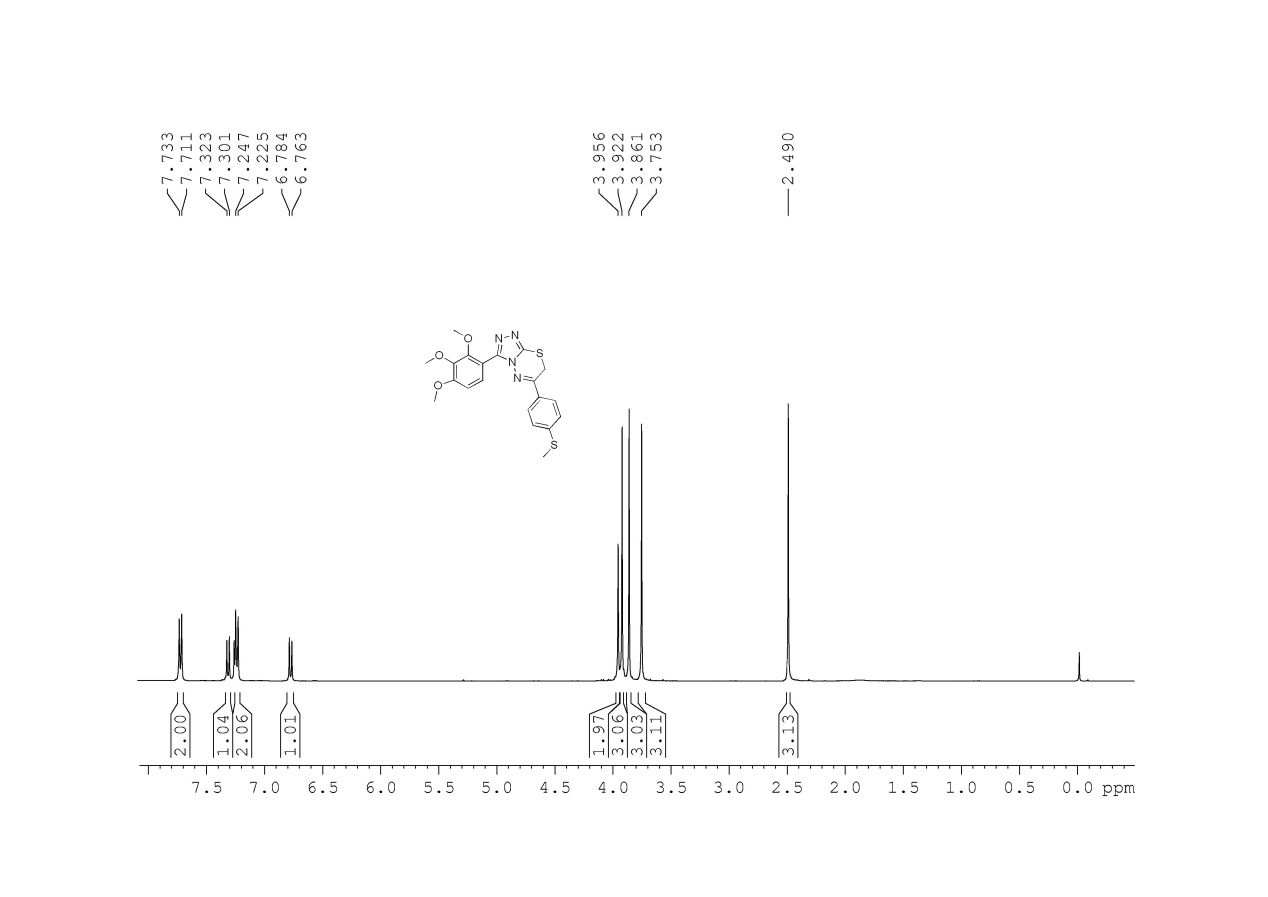

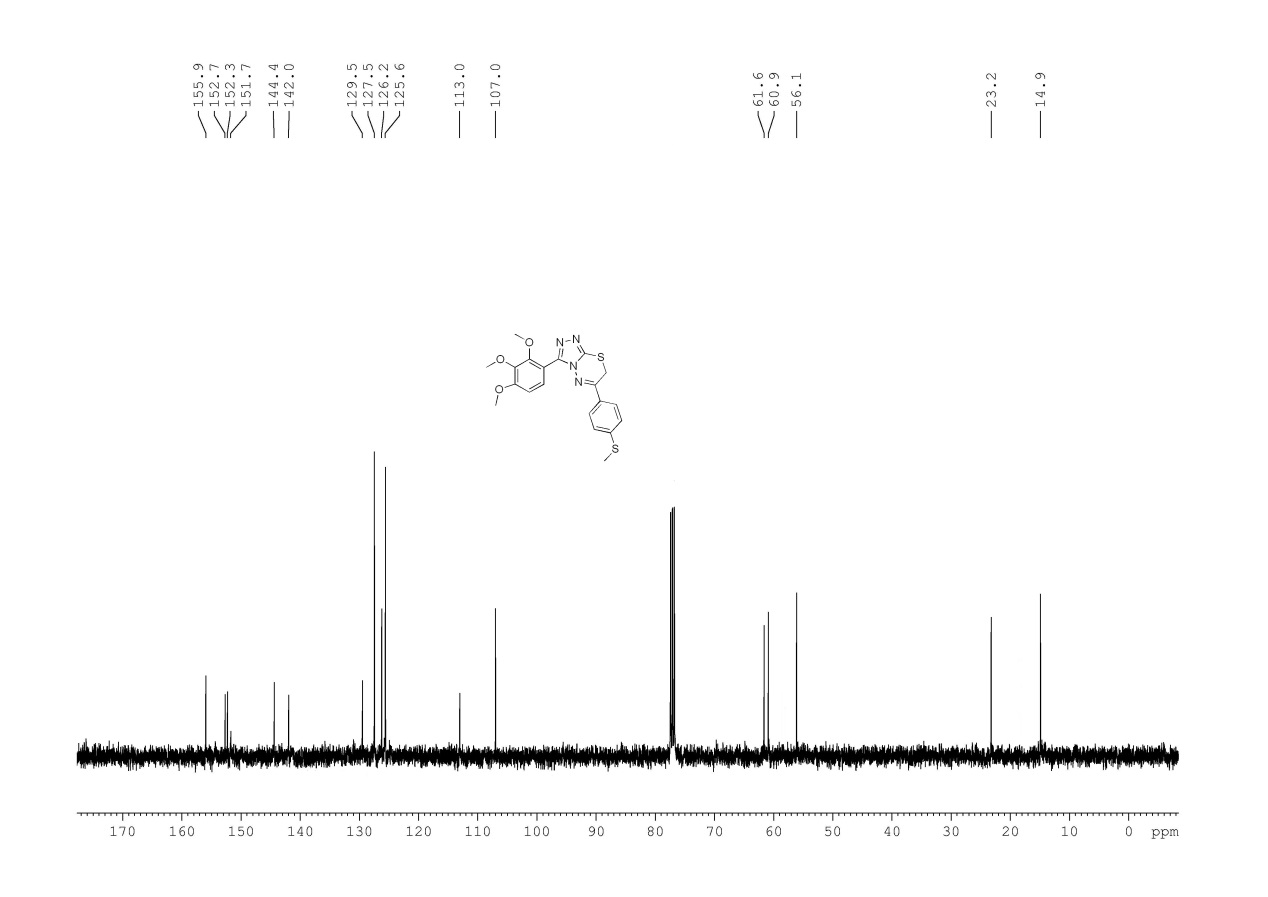


3-(2,3,4-Trimethoxyphenyl)-6-(3-fluoro-4-methoxyphenyl)-*7H*-[1,2,4]triazolo[3,4-b][1,3,4]thiadiazine (**4h**).


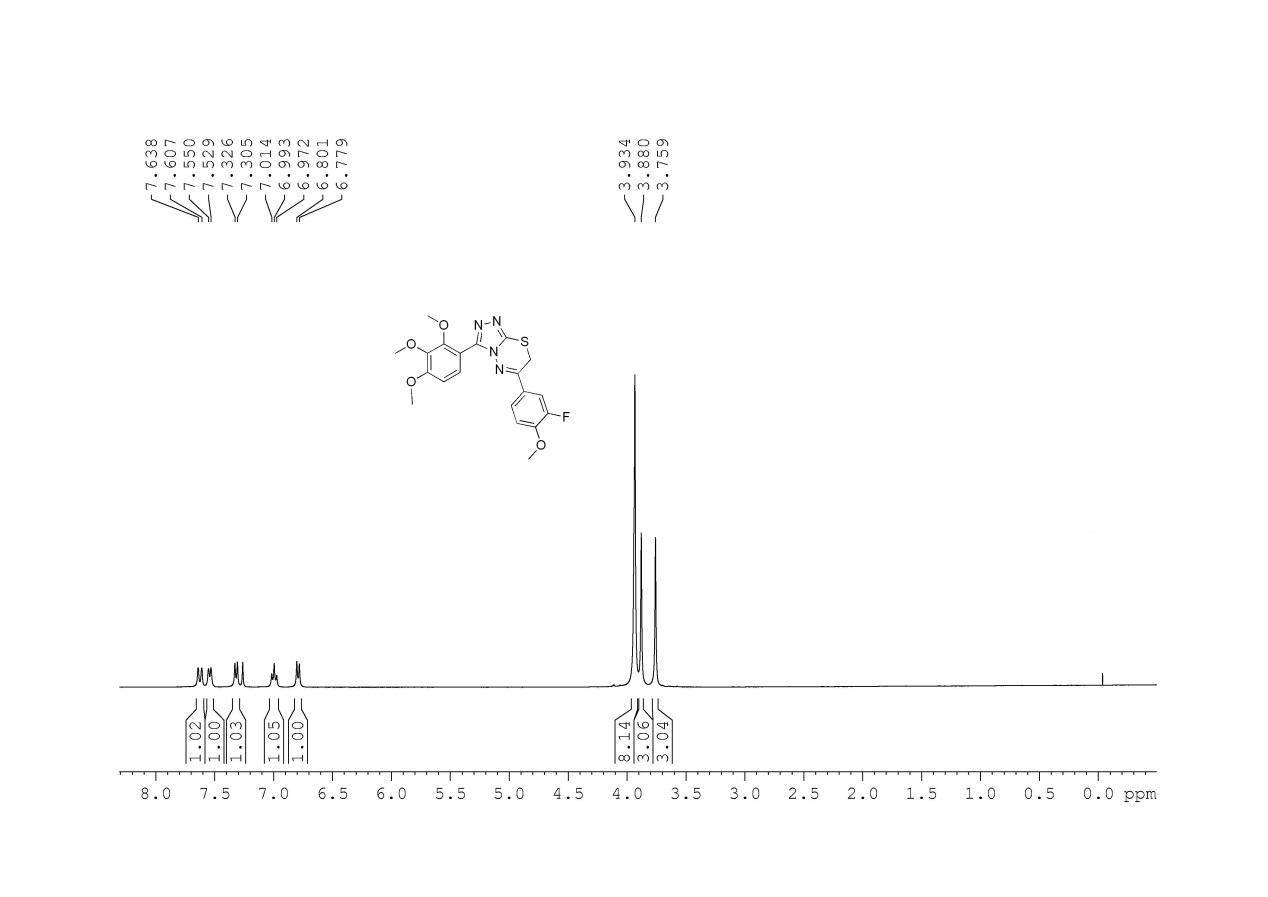

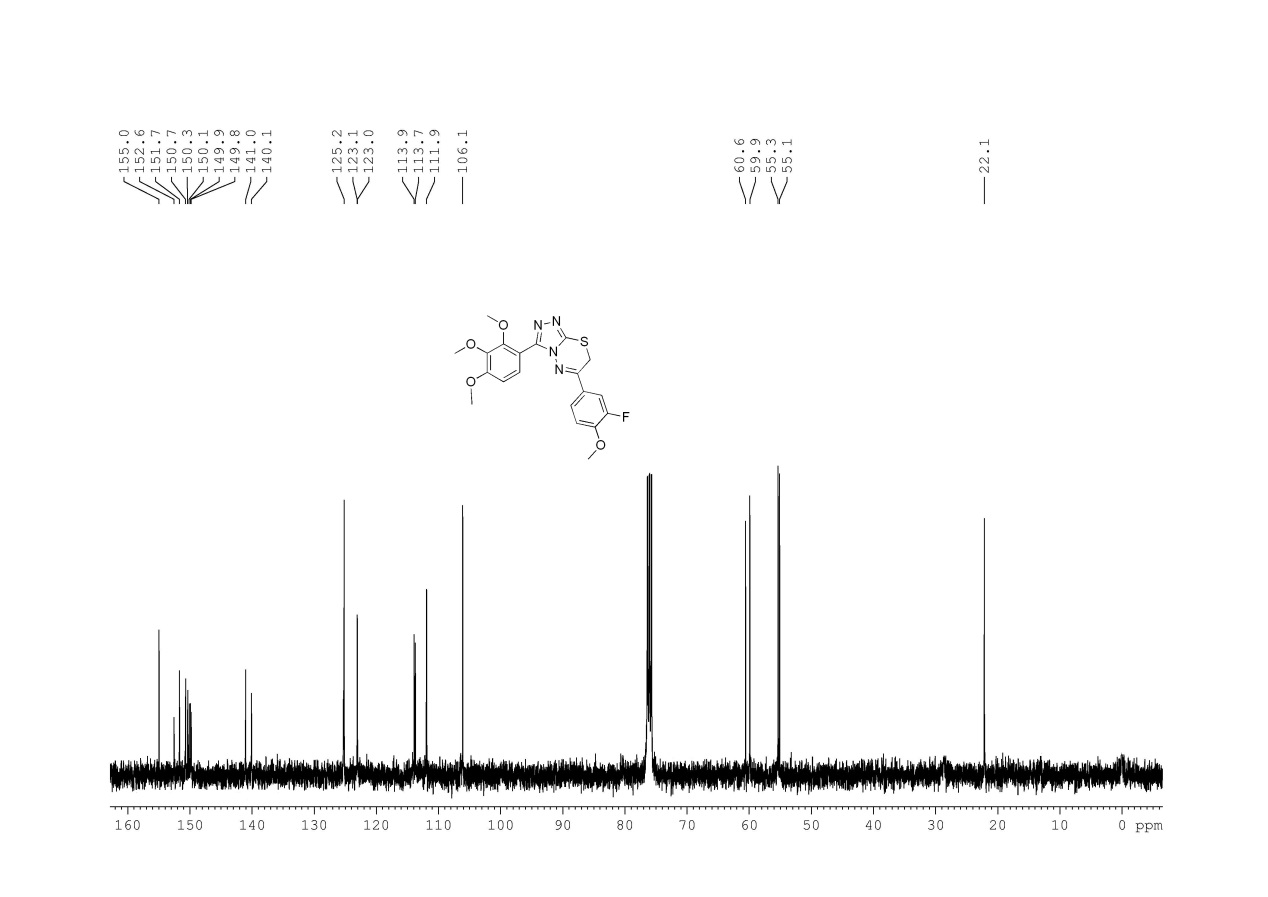


3-(2,3,4-Trimethoxyphenyl)-6-(3-nitro-4-methoxyphenyl)-*7H*-[1,2,4]triazolo[3,4-b][1,3,4]thiadiazine (**4i**).


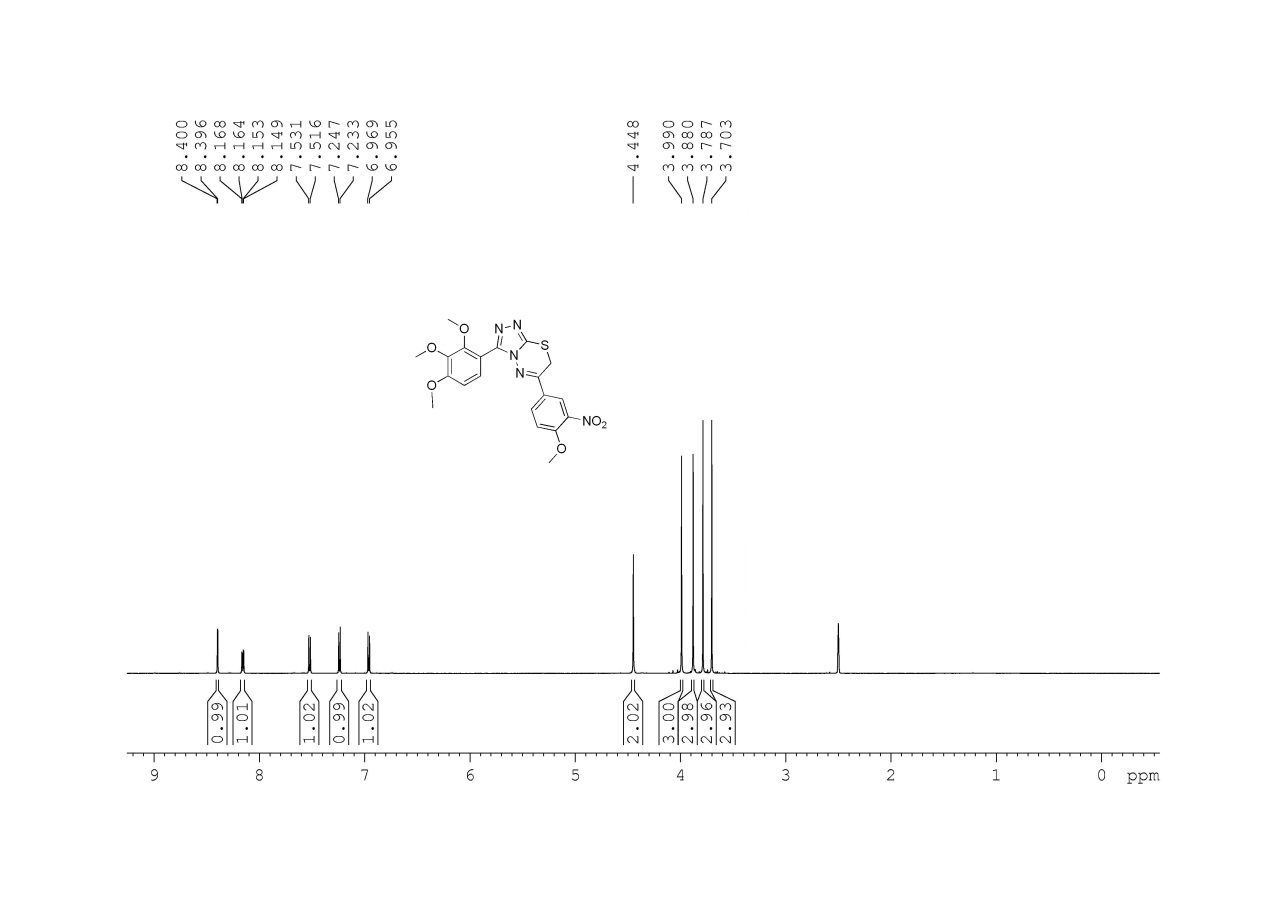

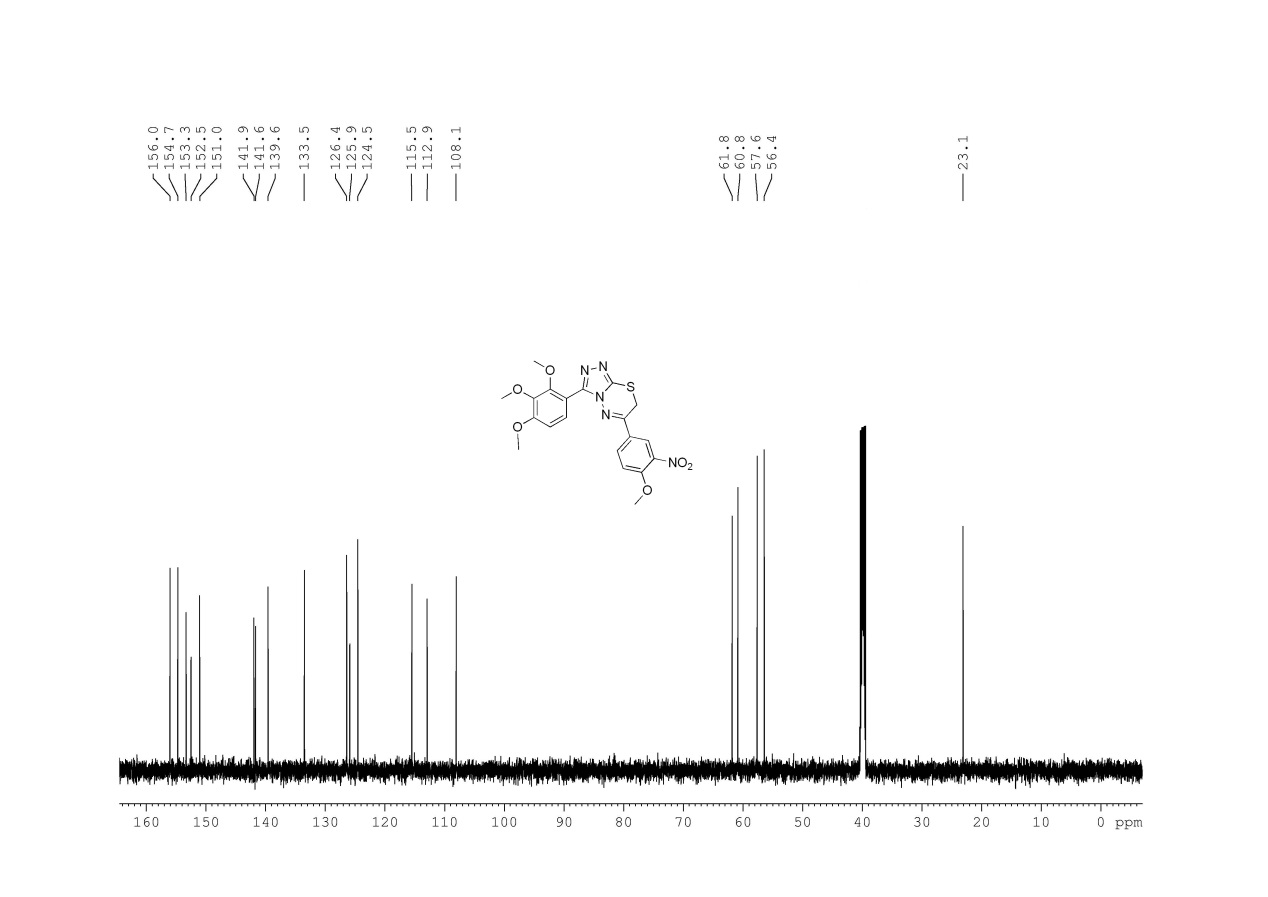


3-(2,3,4-Trimethoxyphenyl)-6-(3-amino-4-methoxyphenyl)-*7H*-[1,2,4]triazolo[3,4-b][1,3,4]thiadiazine (**4j**).


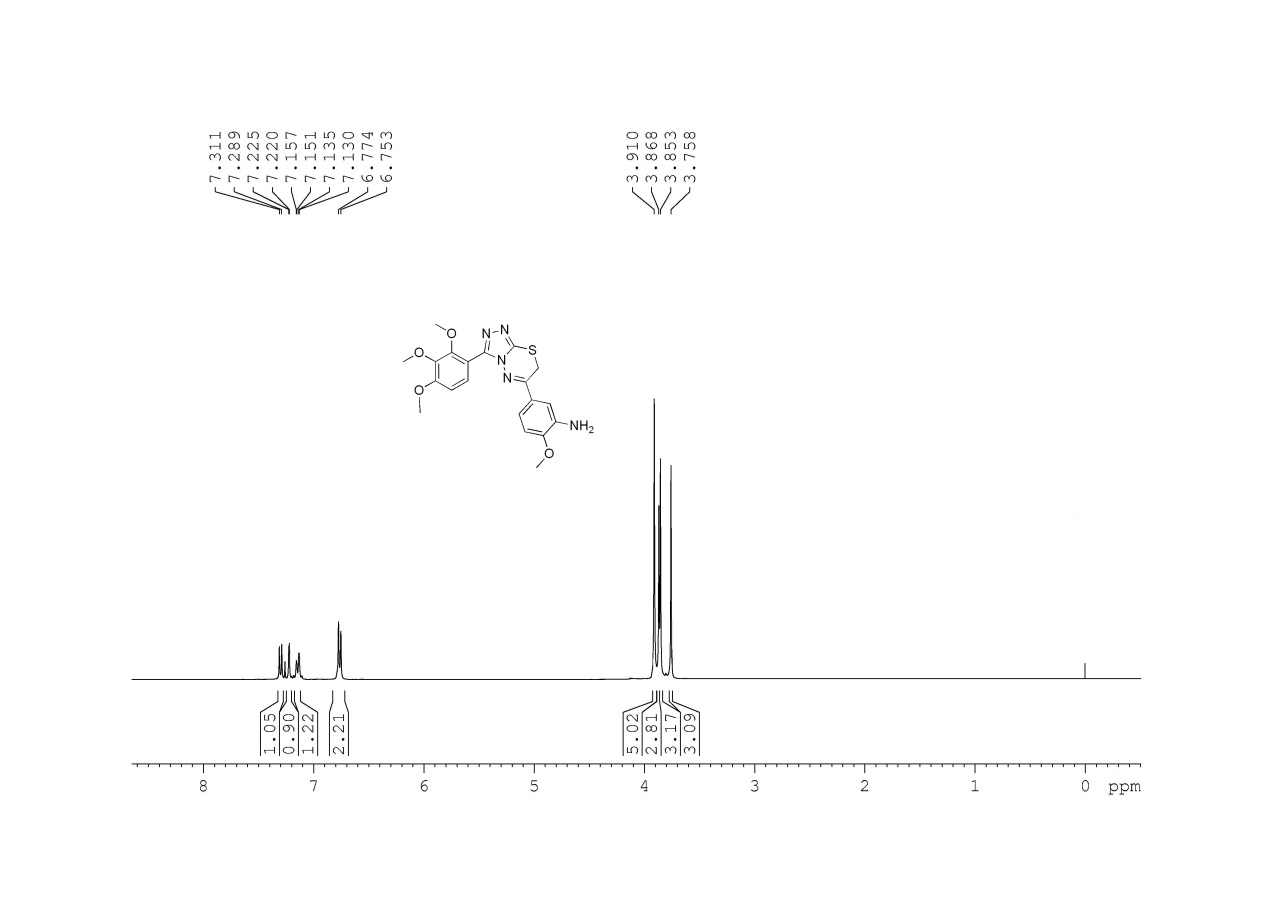

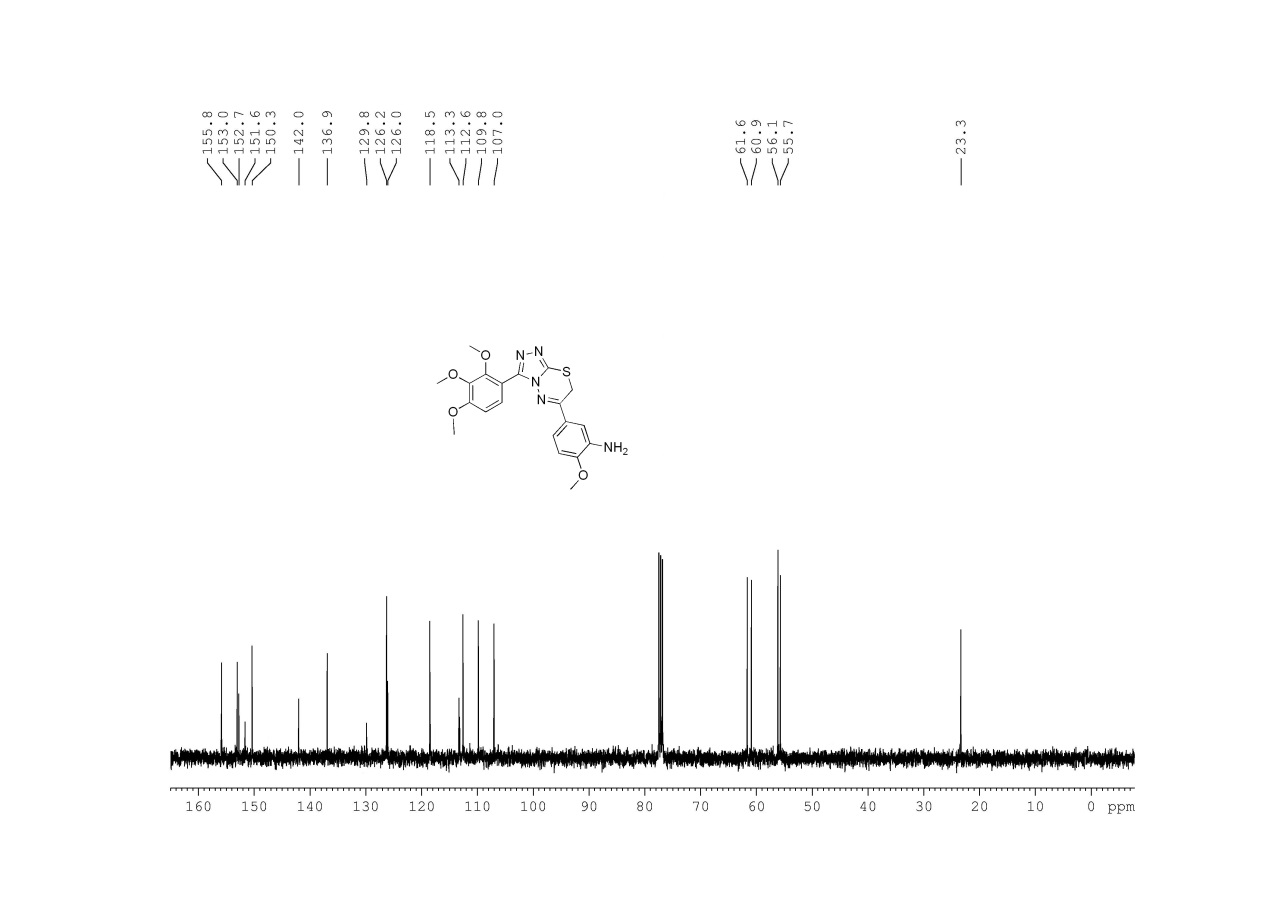


3-(2,3,4-Trimethoxyphenyl)-6-(3-benzyloxy-4-methoxyphenyl)-*7H*-[1,2,4]triazolo[3,4-b][1,3,4]thiadiazine (**4k**).


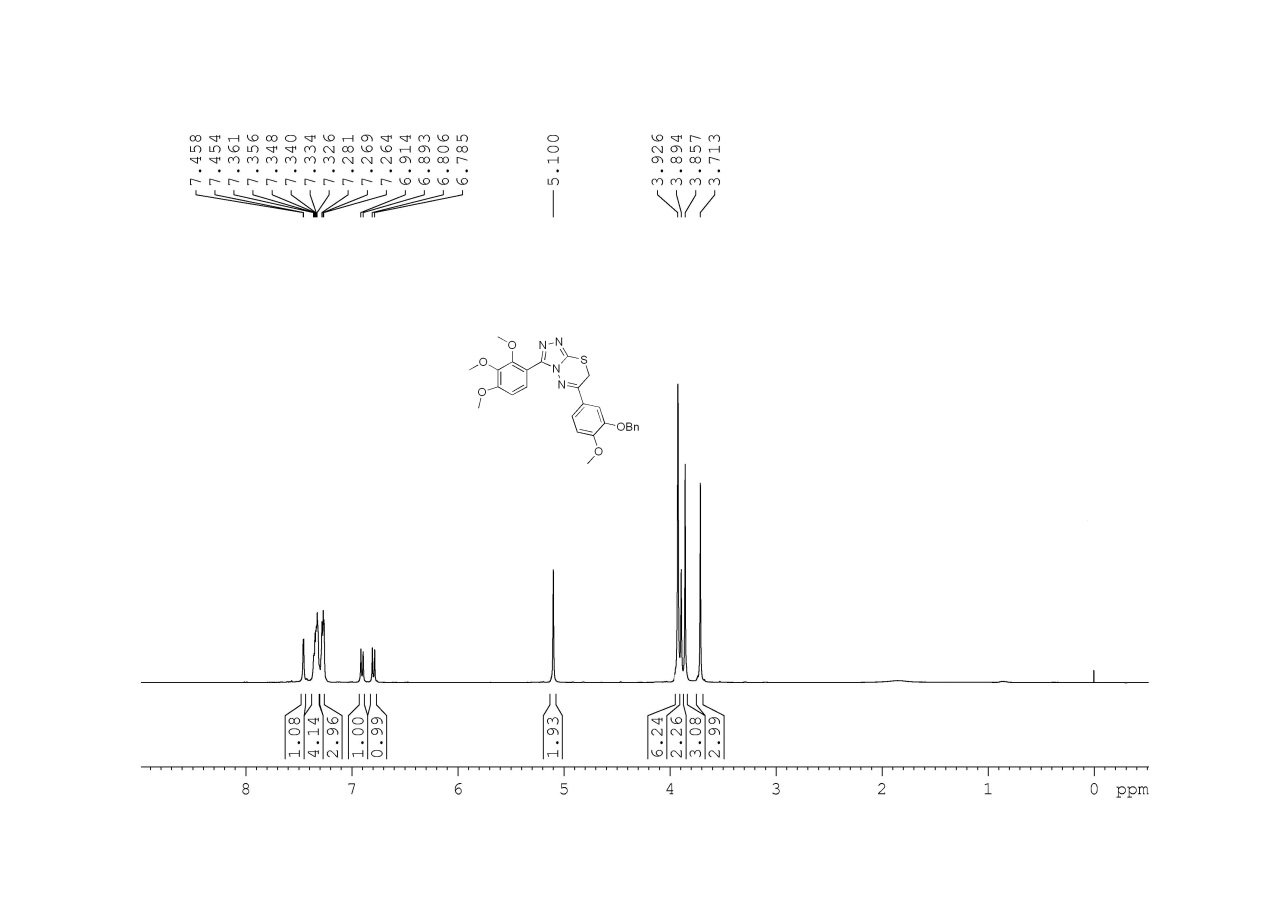

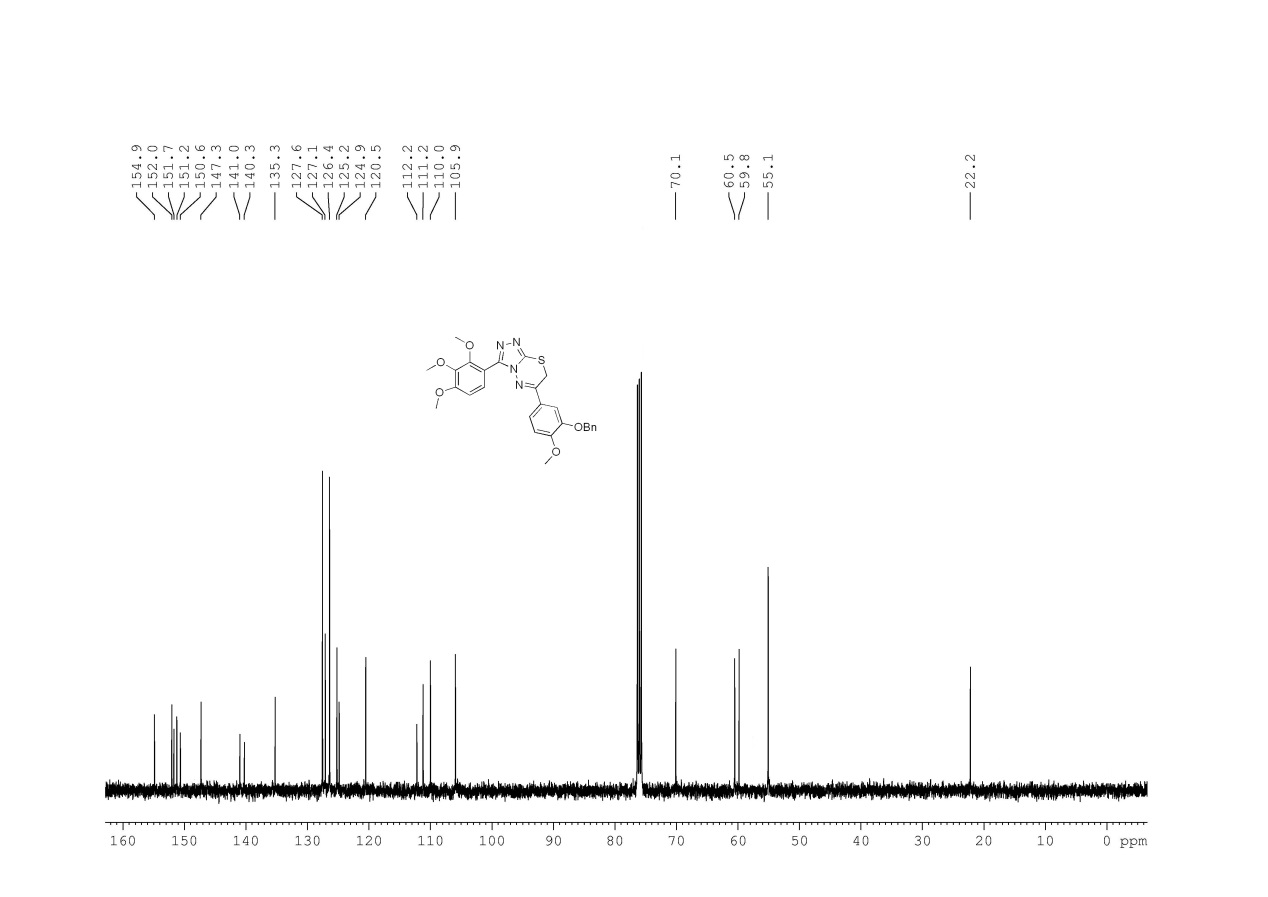


3-(2,3,4-Trimethoxyphenyl)-6-(3-hydroxy-4-methoxyphenyl)-*7H*-[1,2,4]triazolo[3,4-b][1,3,4]thiadiazine (**4l**).


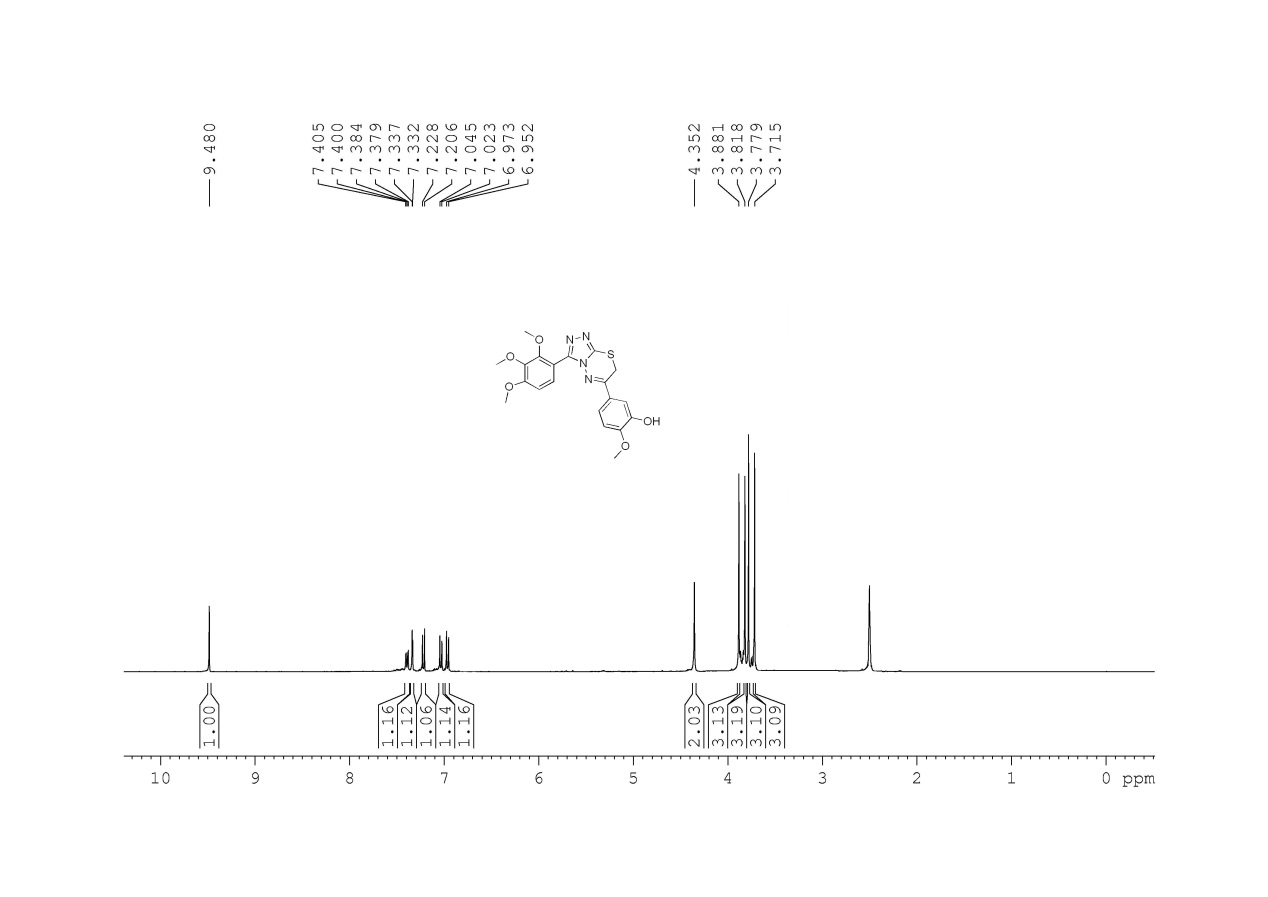

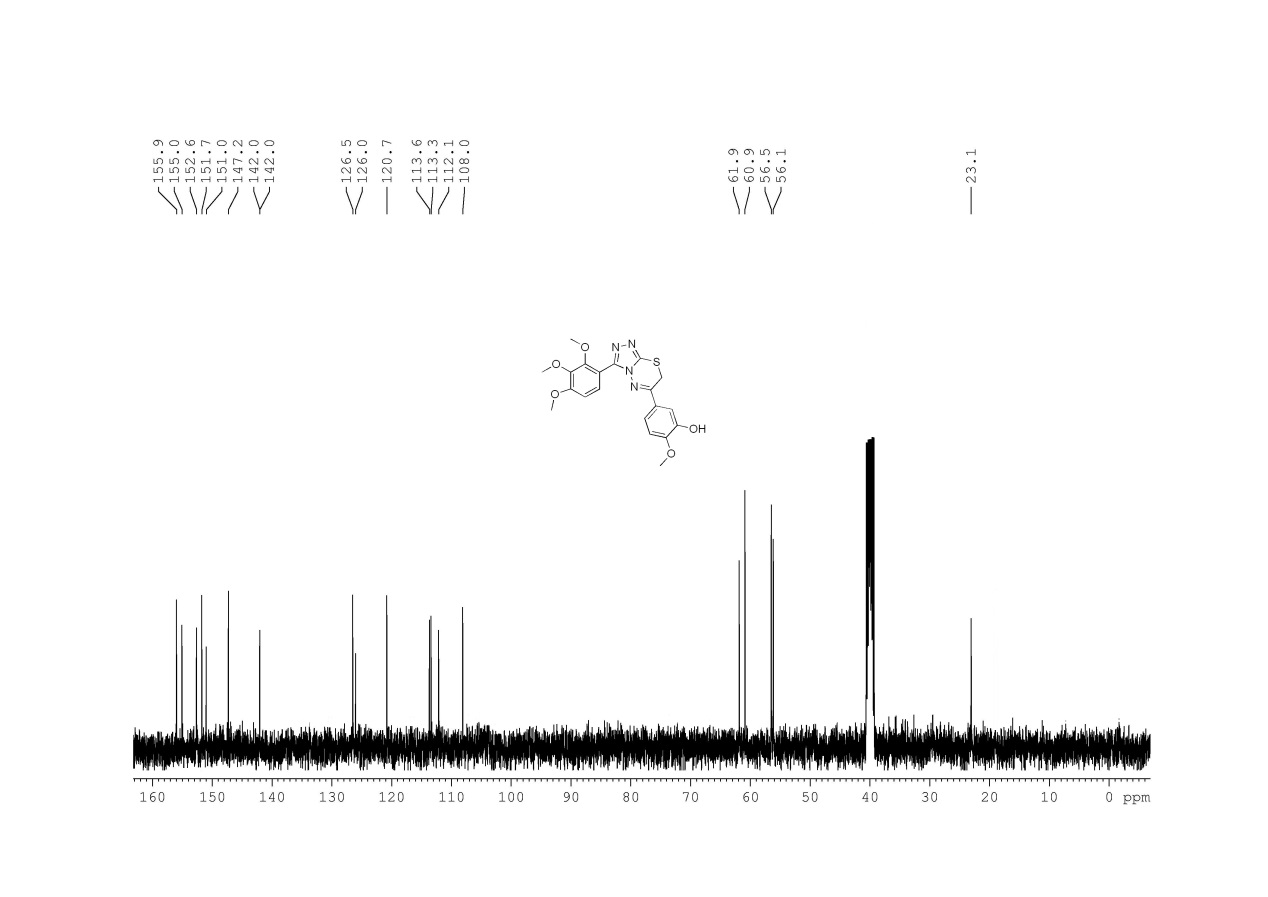


3-(2,3,4-Trimethoxyphenyl)-6-(3,4-difluorophenyl)-*7H*-[1,2,4]triazolo[3,4-b][1,3,4]thiadiazine (**4m**).


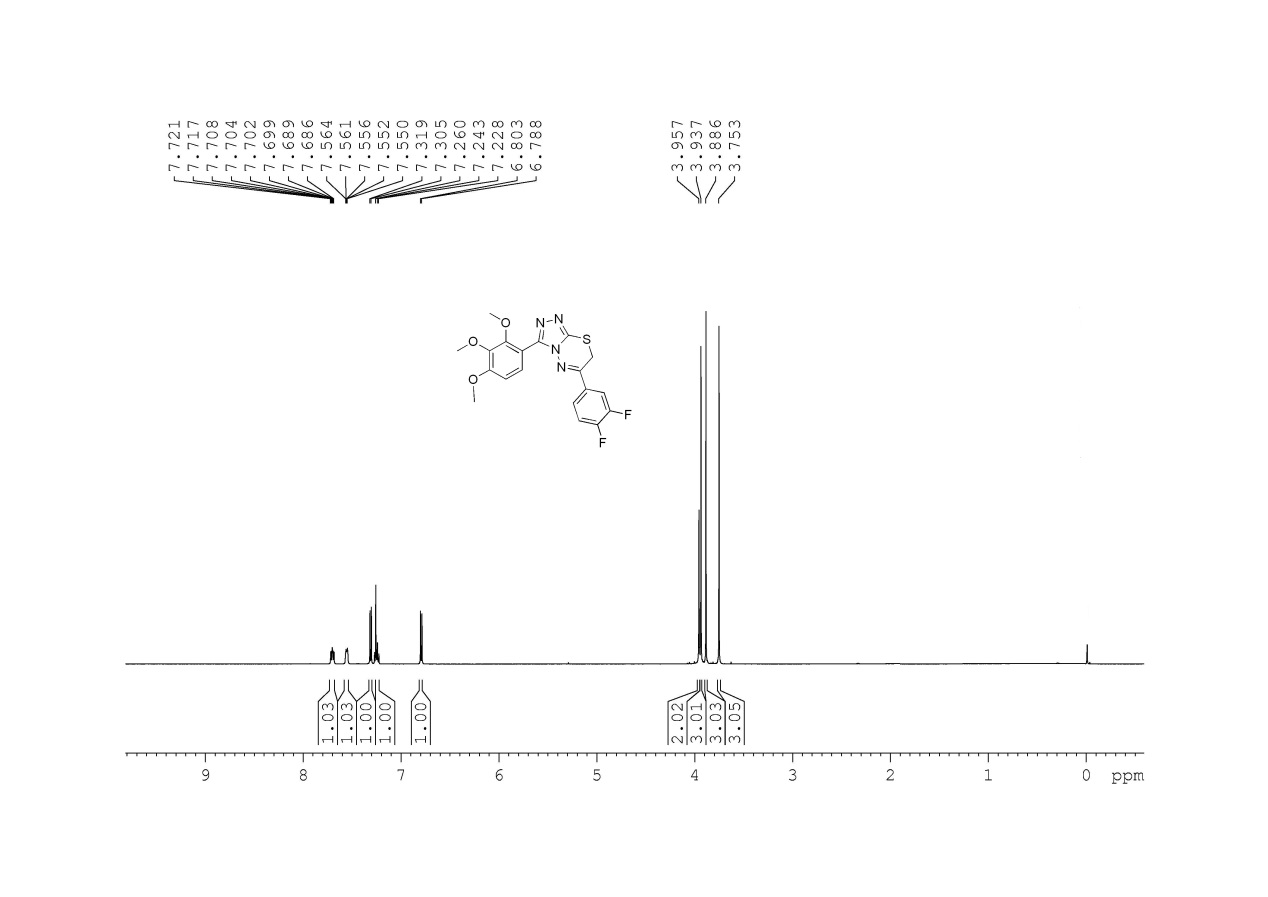

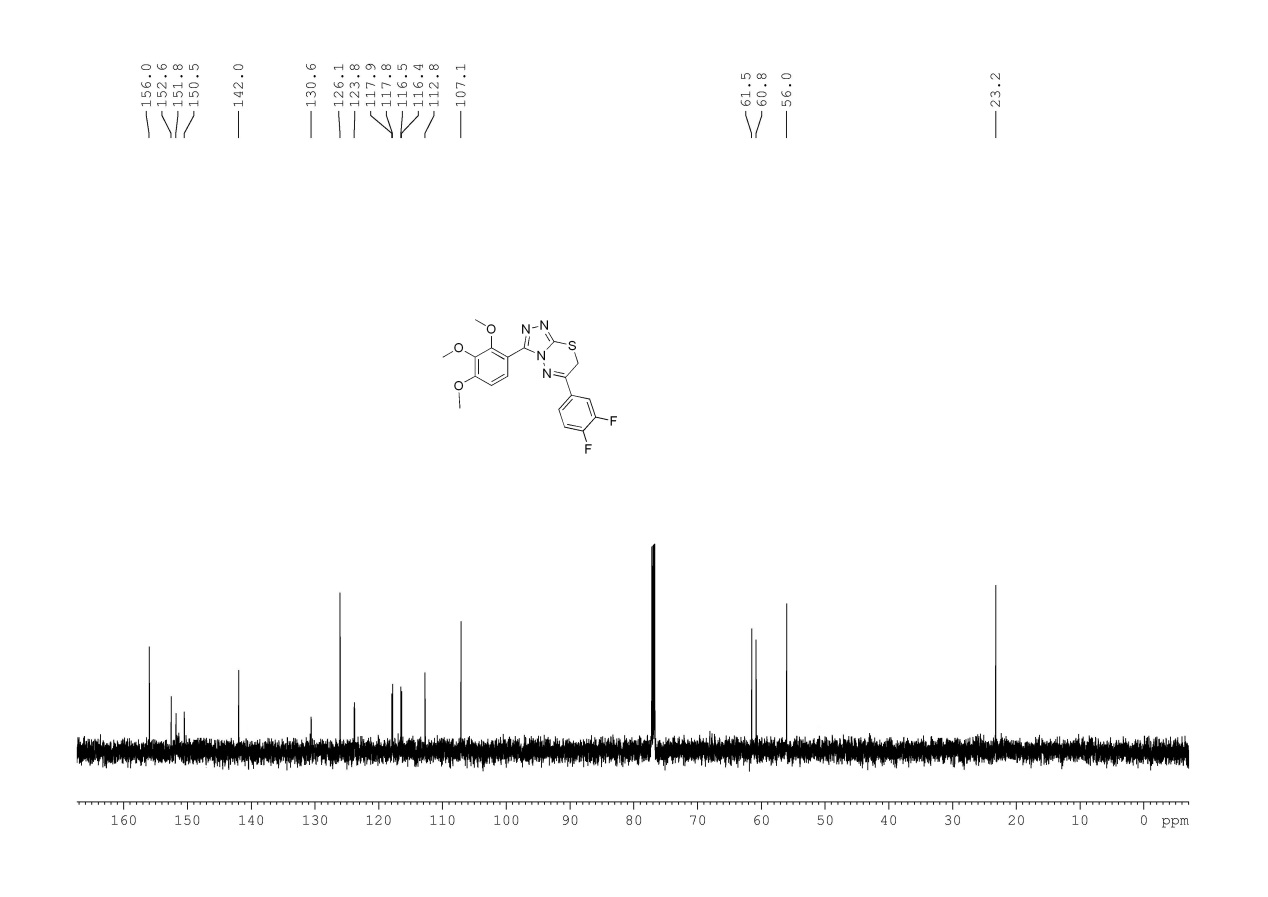


3-(3,4,5-Trimethoxyphenyl)-6-(4-chlorophenyl)-*7H*-[1,2,4]triazolo[3,4-b][1,3,4]thiadiazine (**5a**).


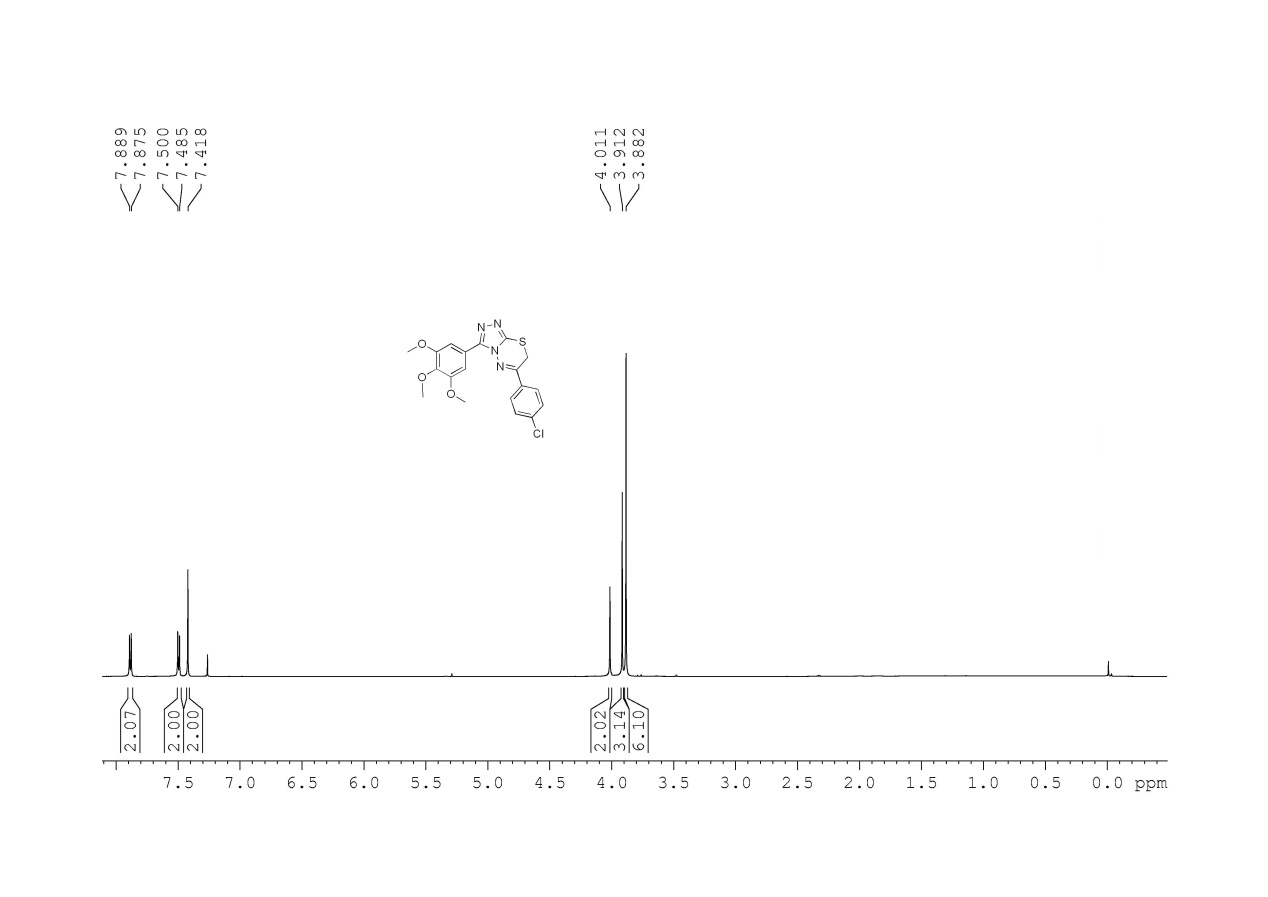

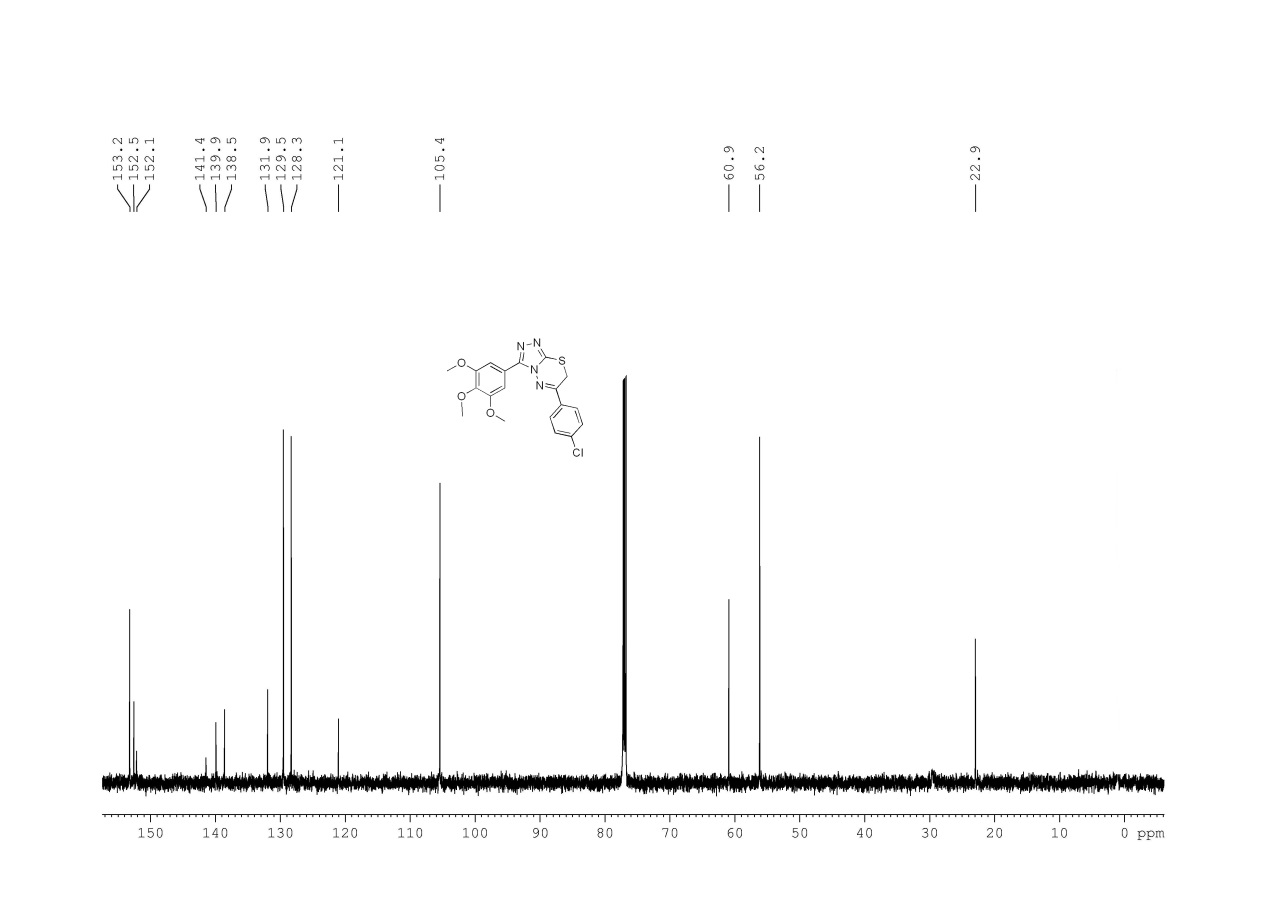


3-(3,4,5-Trimethoxyphenyl)-6-(4-methyphenyl)-*7H*-[1,2,4]triazolo[3,4-b][1,3,4]thiadiazine (**5b**).


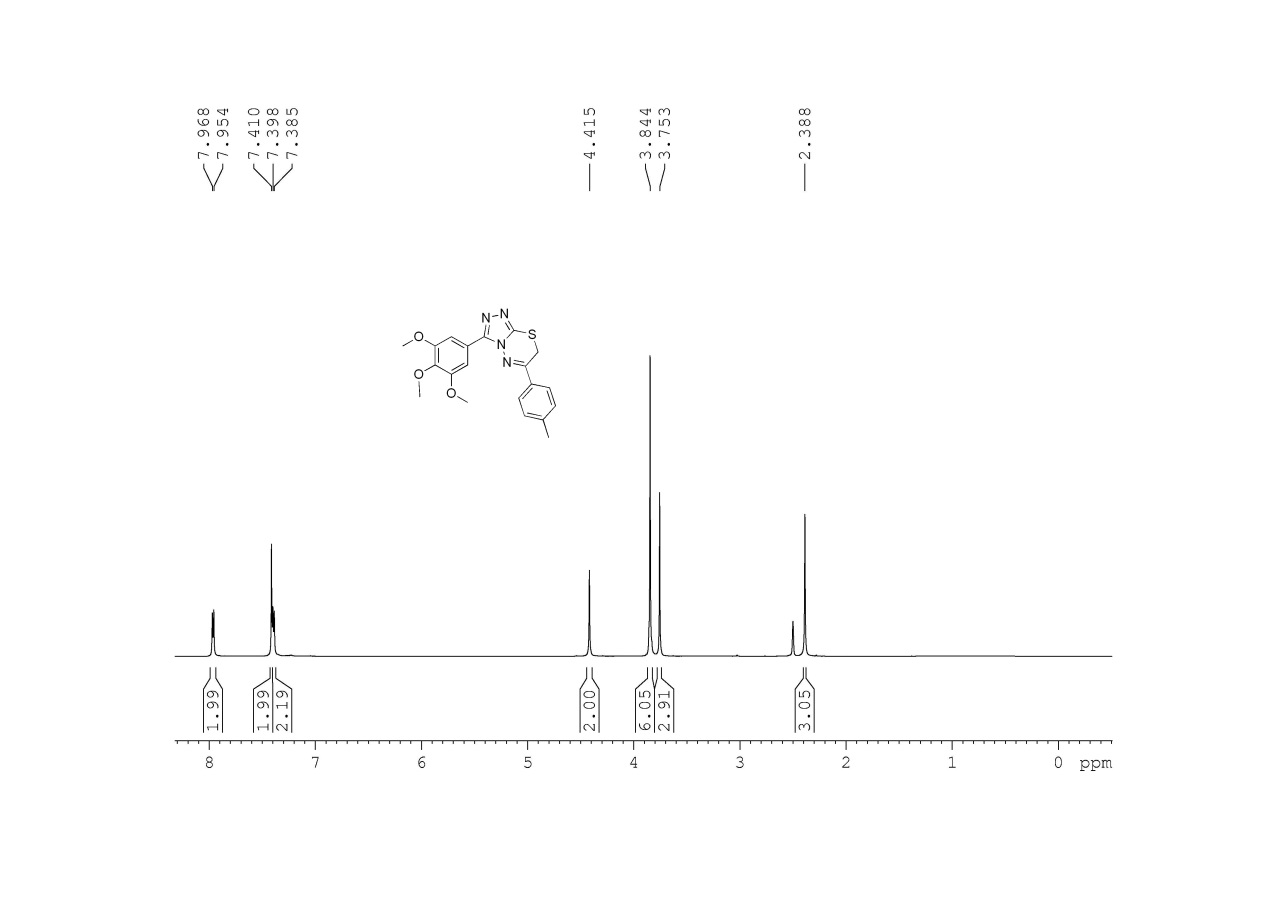

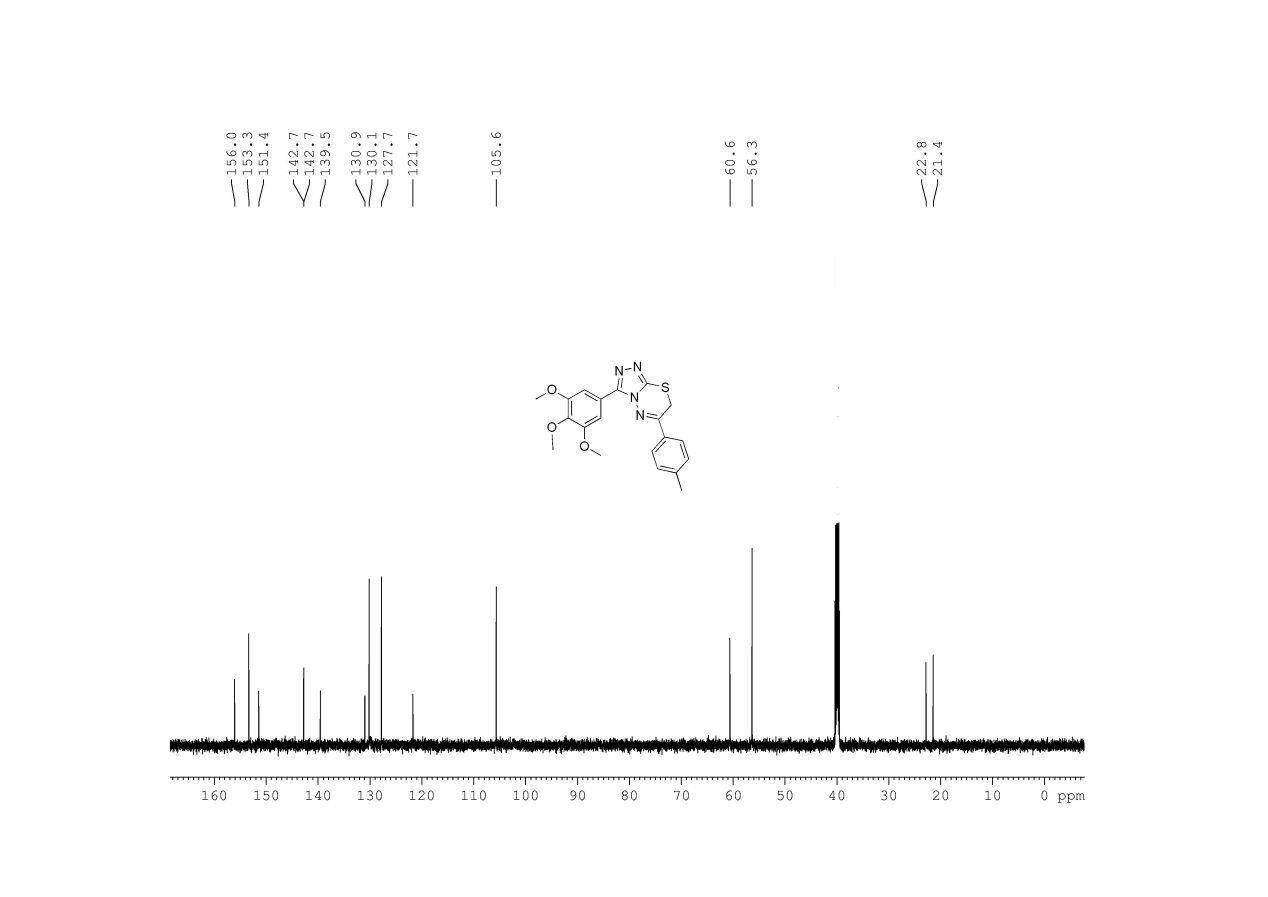


3-(3,4,5-Trimethoxyphenyl)-6-(4-(trifluoromethyl)phenyl)-*7H*-[1,2,4]triazolo[3,4-b][1,3,4]thiadiazine (**5c**).


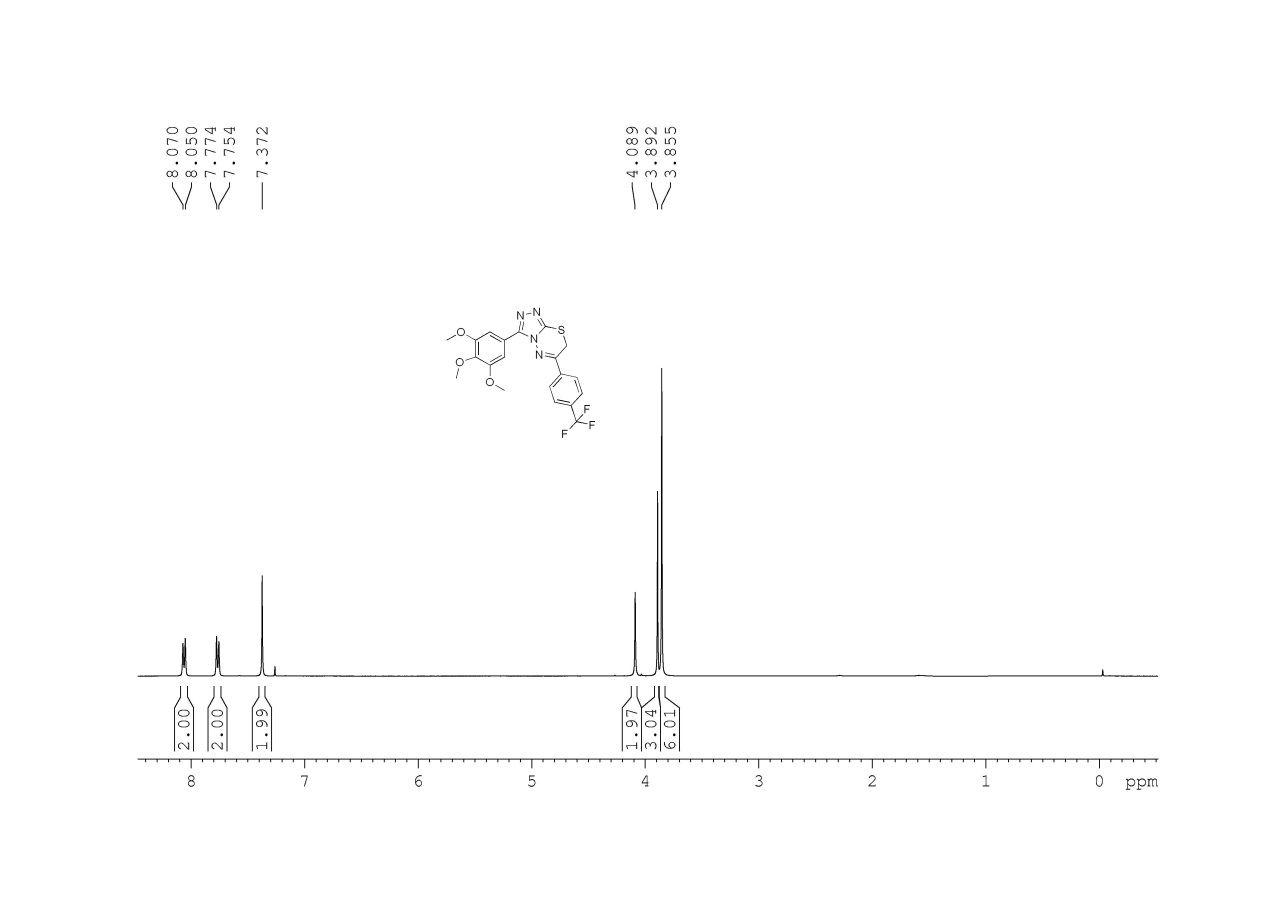

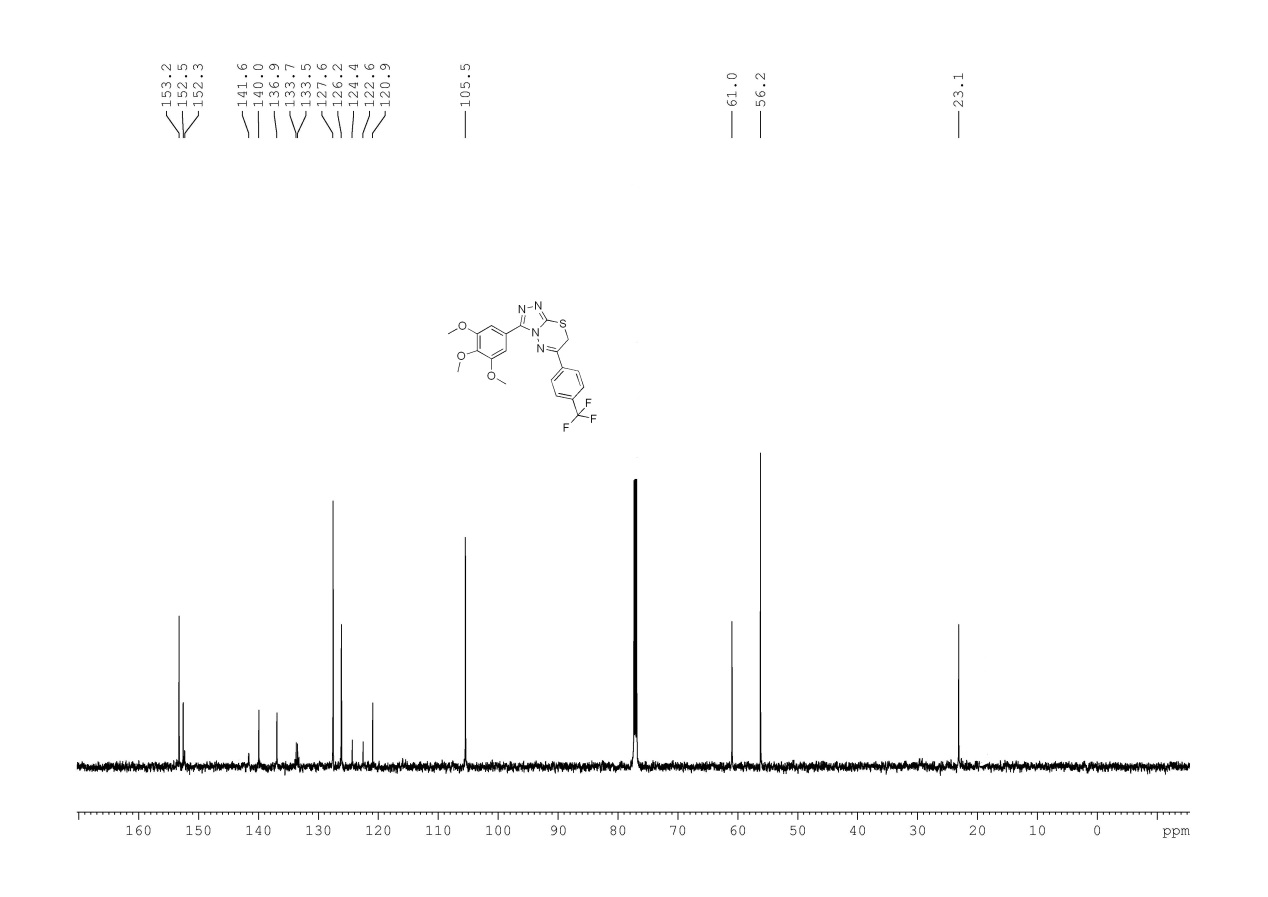


3-(3,4,5-Trimethoxyphenyl)-6-(4-methylthiophenyl)-*7H*-[1,2,4]triazolo[3,4-b][1,3,4]thiadiazine (**5d**).


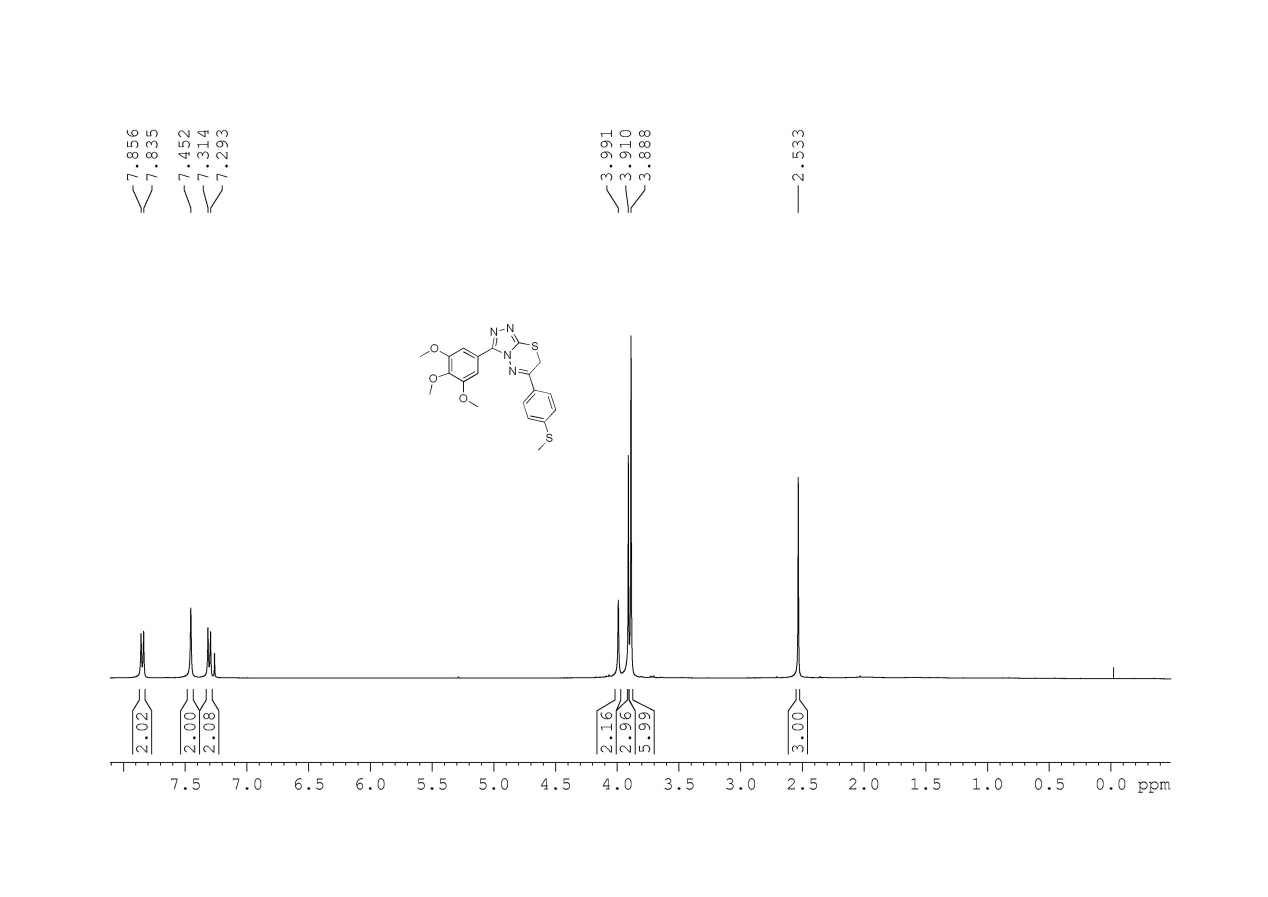

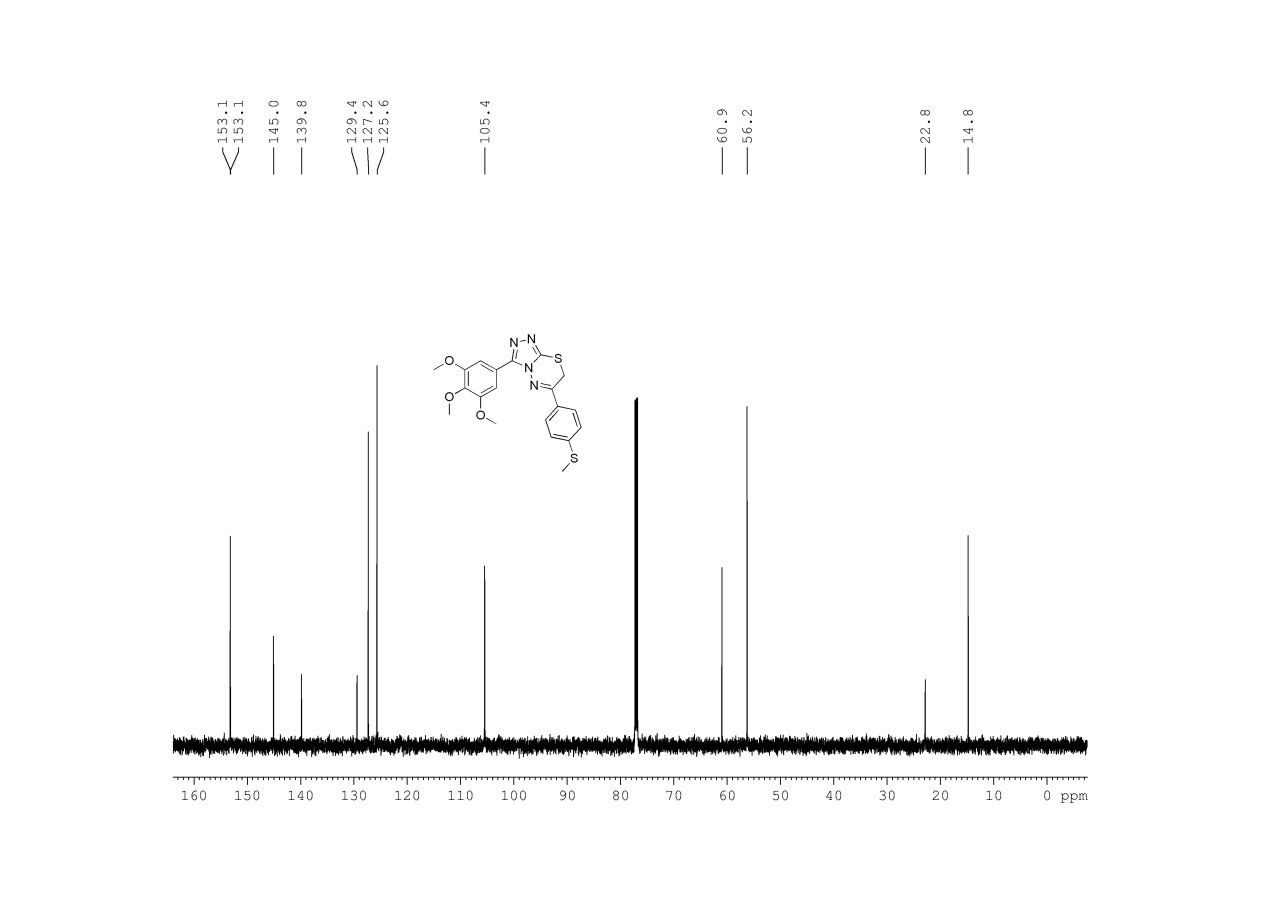


3-(3,4,5-Trimethoxyphenyl)-6-(3-fluoro-4-methoxyphenyl)-*7H*-[1,2,4]triazolo[3,4-b][1,3,4]thiadiazine (**5e**).


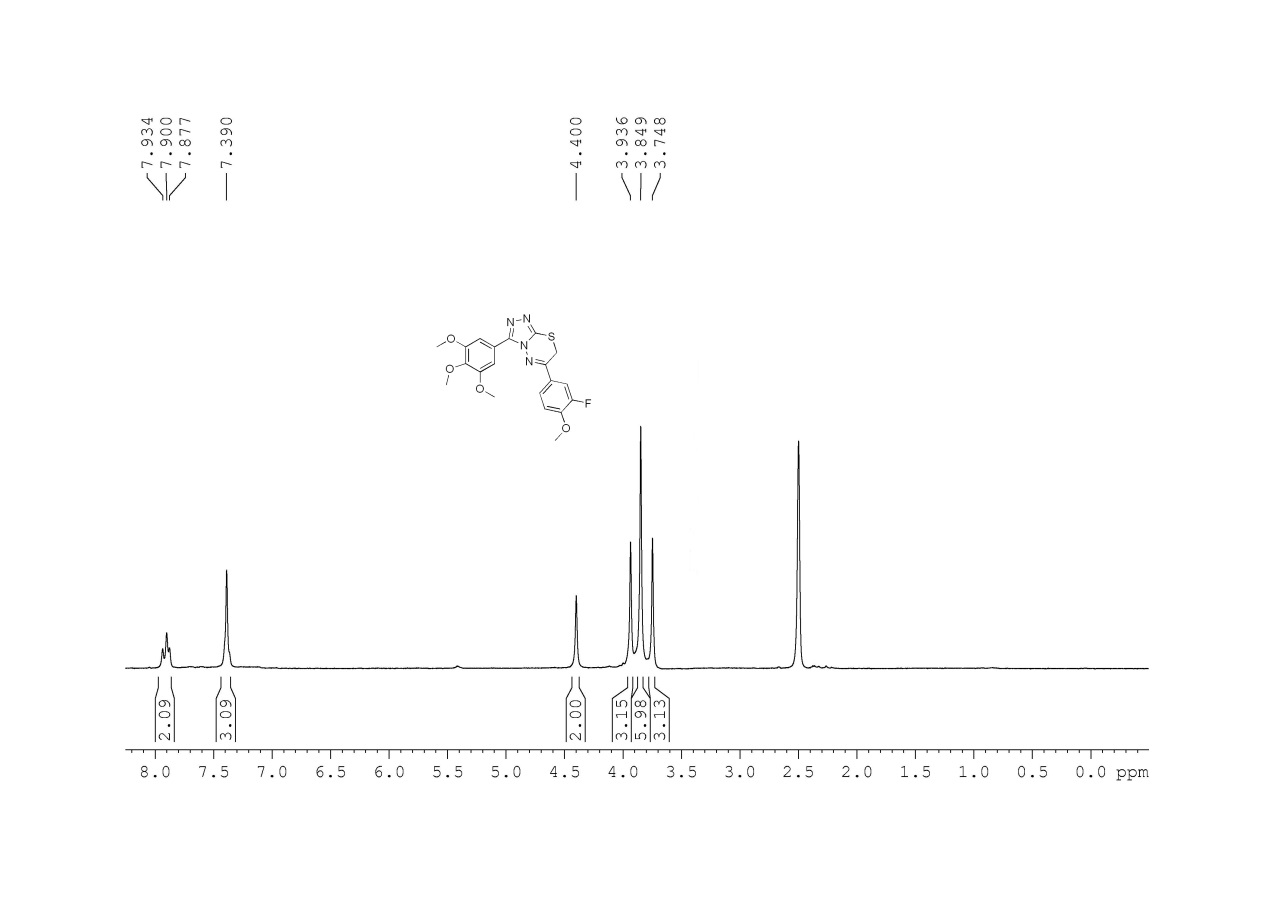

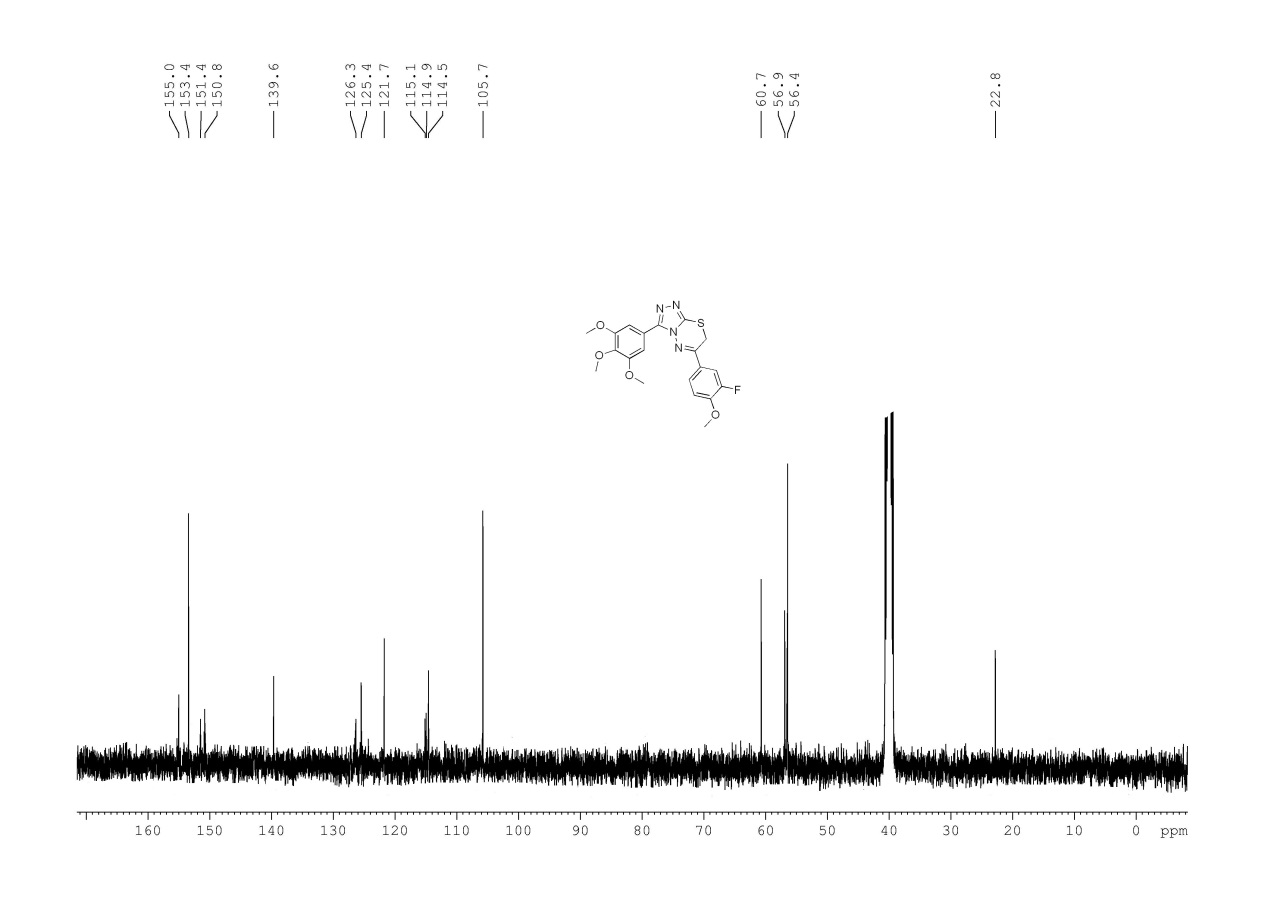


3-(3,4,5-Trimethoxyphenyl)-6-(3-nitro-4-methoxyphenyl)-*7H*-[1,2,4]triazolo[3,4-b][1,3,4]thiadiazine (**5f**).


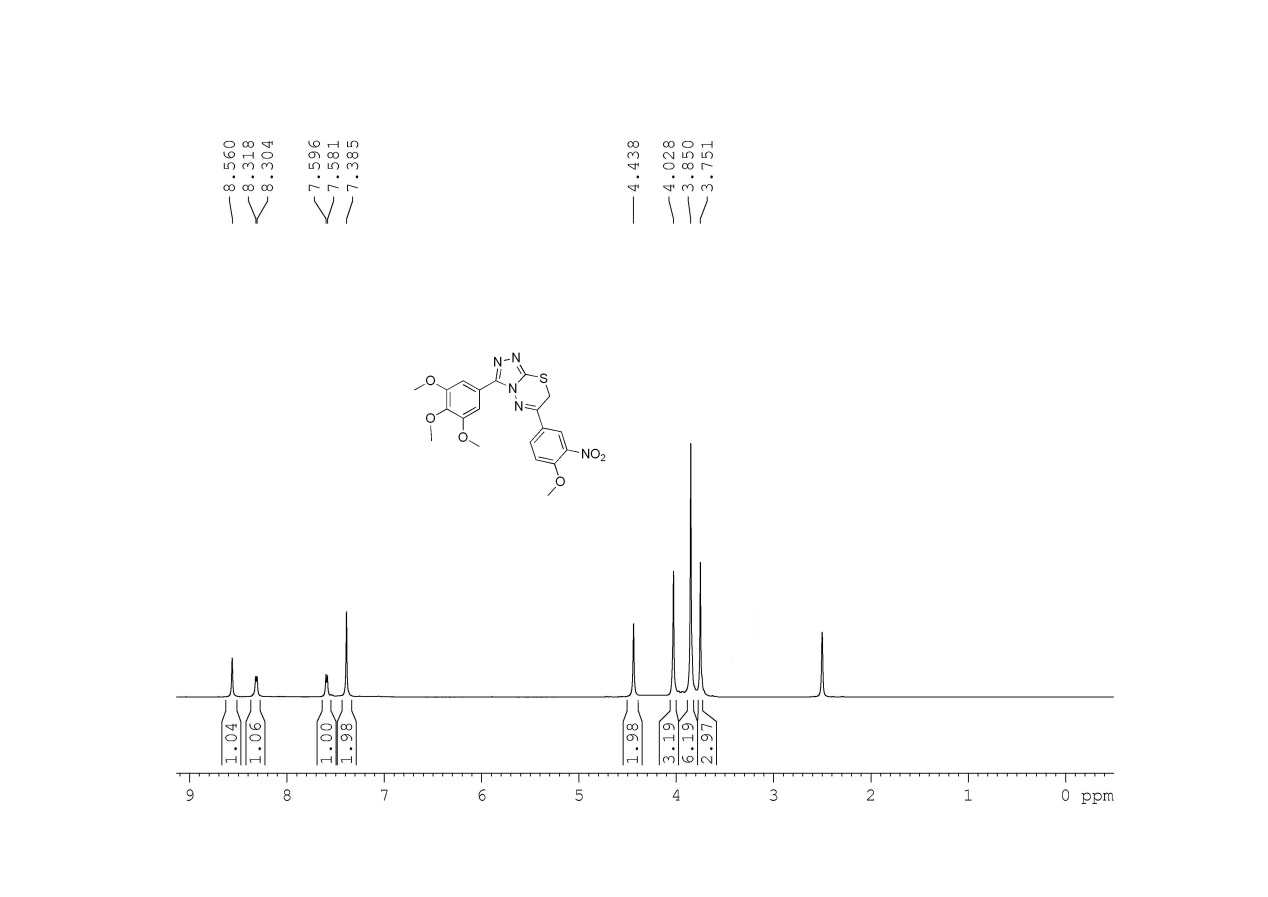

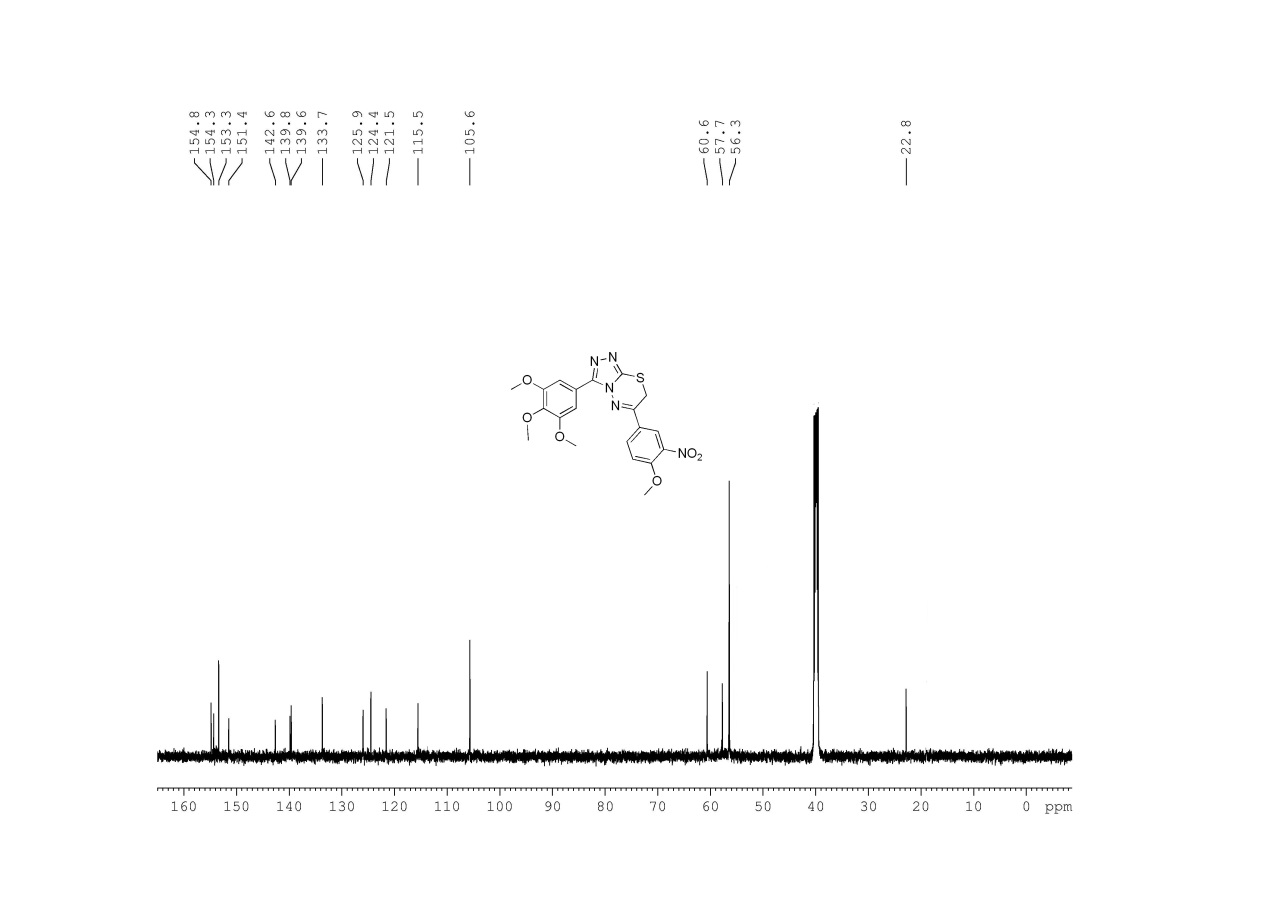


3-(3,4,5-Trimethoxyphenyl)-6-(3-amino-4-methoxyphenyl)-*7H*-[1,2,4]triazolo[3,4-b][1,3,4]thiadiazine (**5g**).


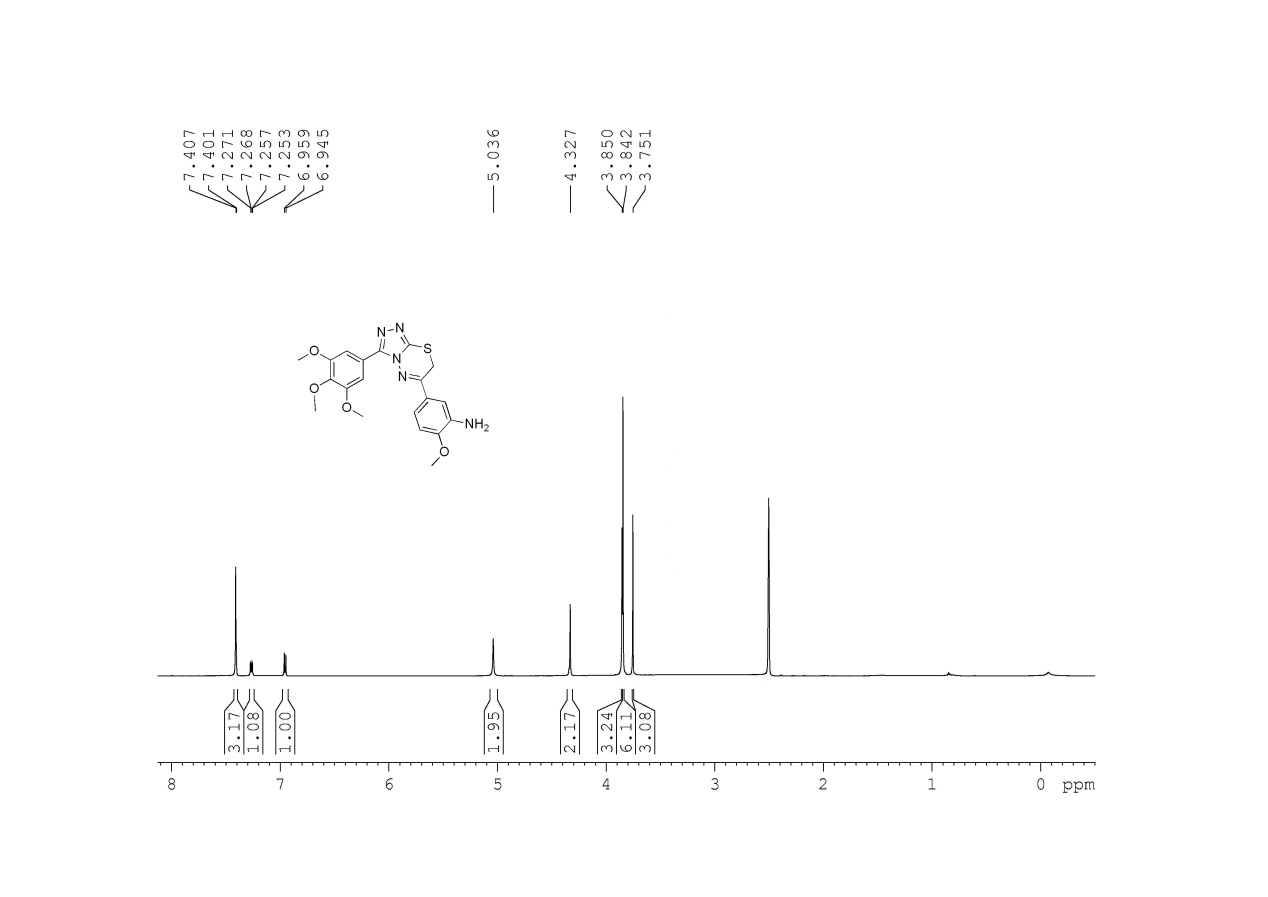

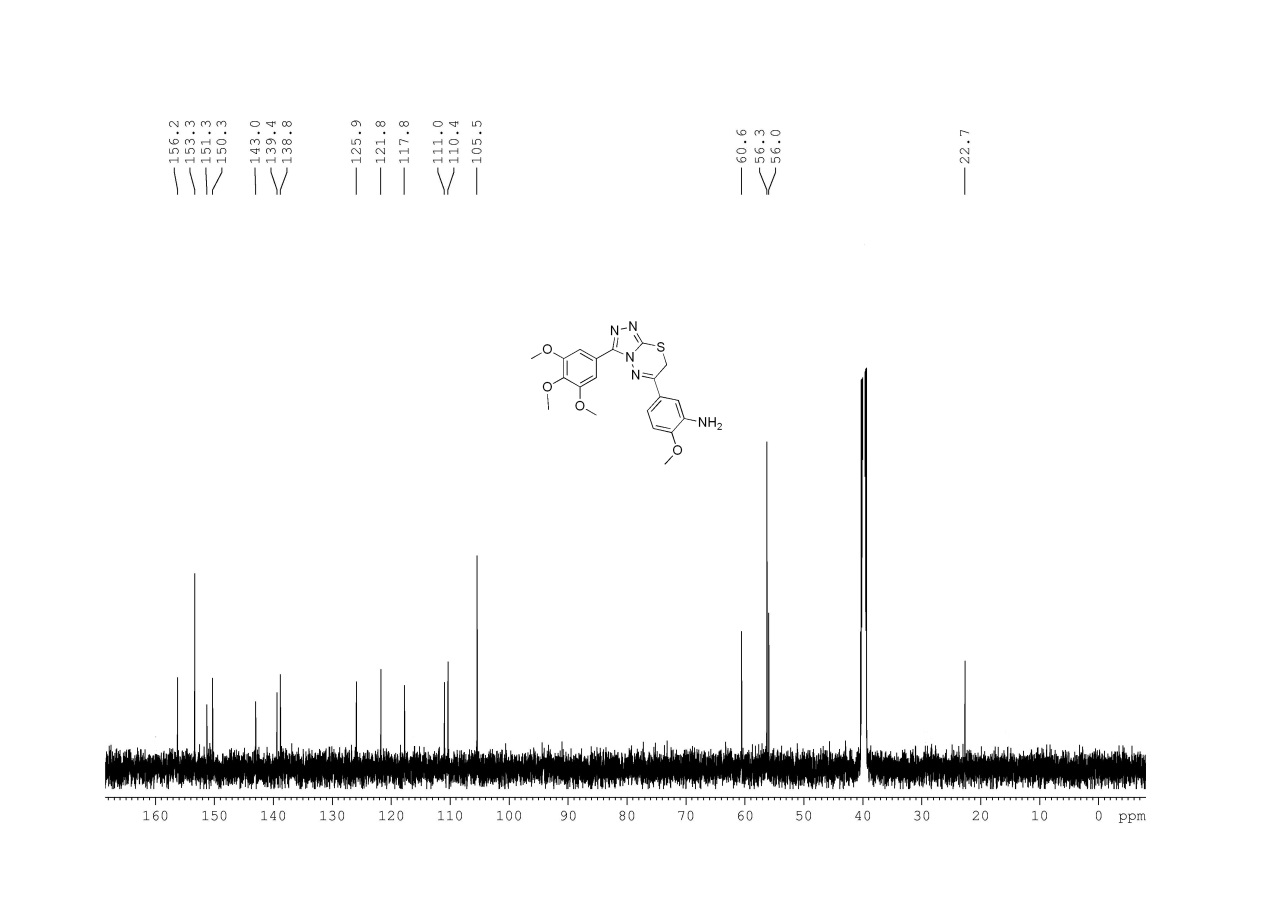


3-(3,4,5-Trimethoxyphenyl)-6-(3-benzyloxy-4-methoxyphenyl)-*7H*-[1,2,4]triazolo[3,4-b][1,3,4]thiadiazine (**5h**).


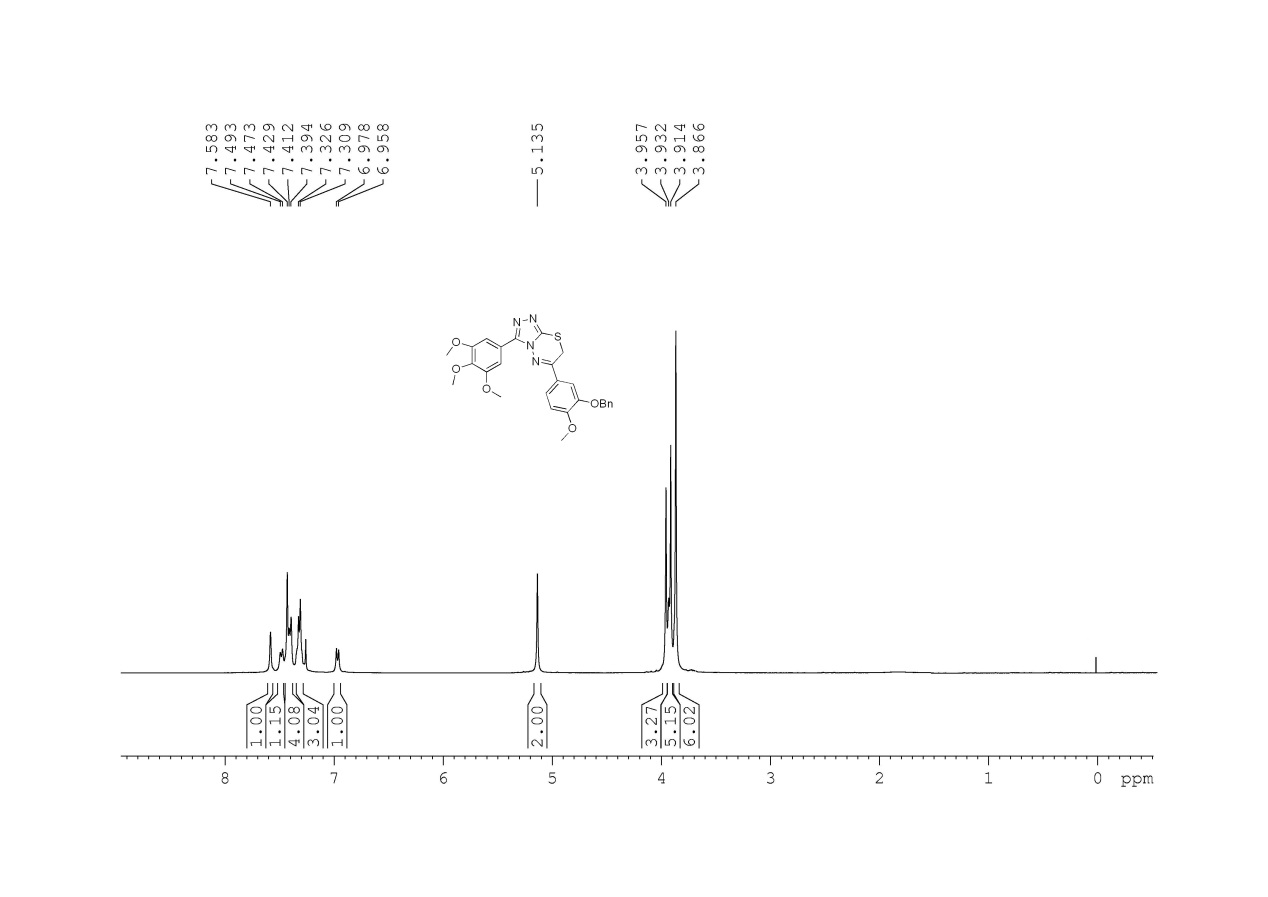

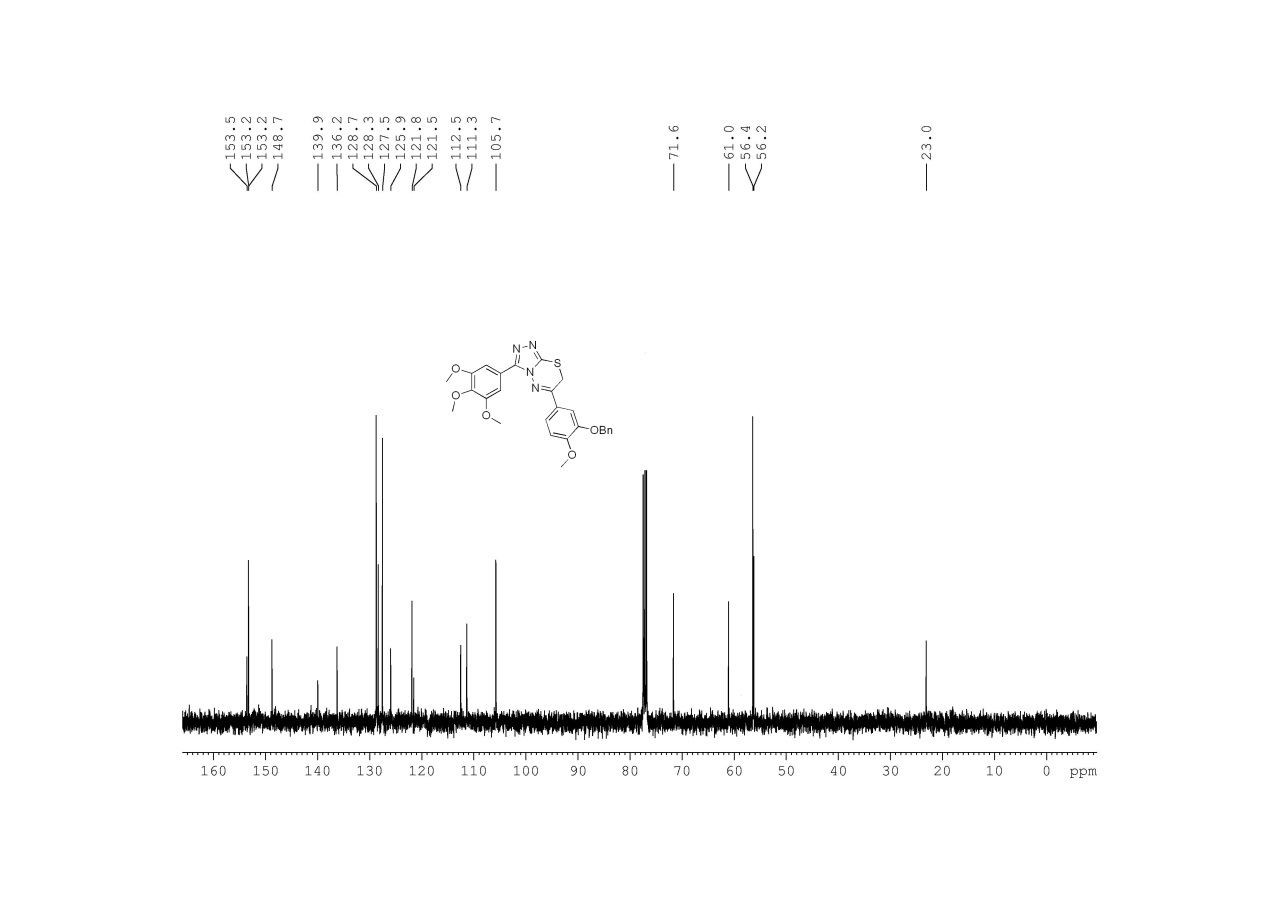


3-(3,4,5-Trimethoxyphenyl)-6-(3-hydroxy-4-methoxyphenyl)-*7H*-[1,2,4]triazolo[3,4-b][1,3,4]thiadiazine (**5i**).


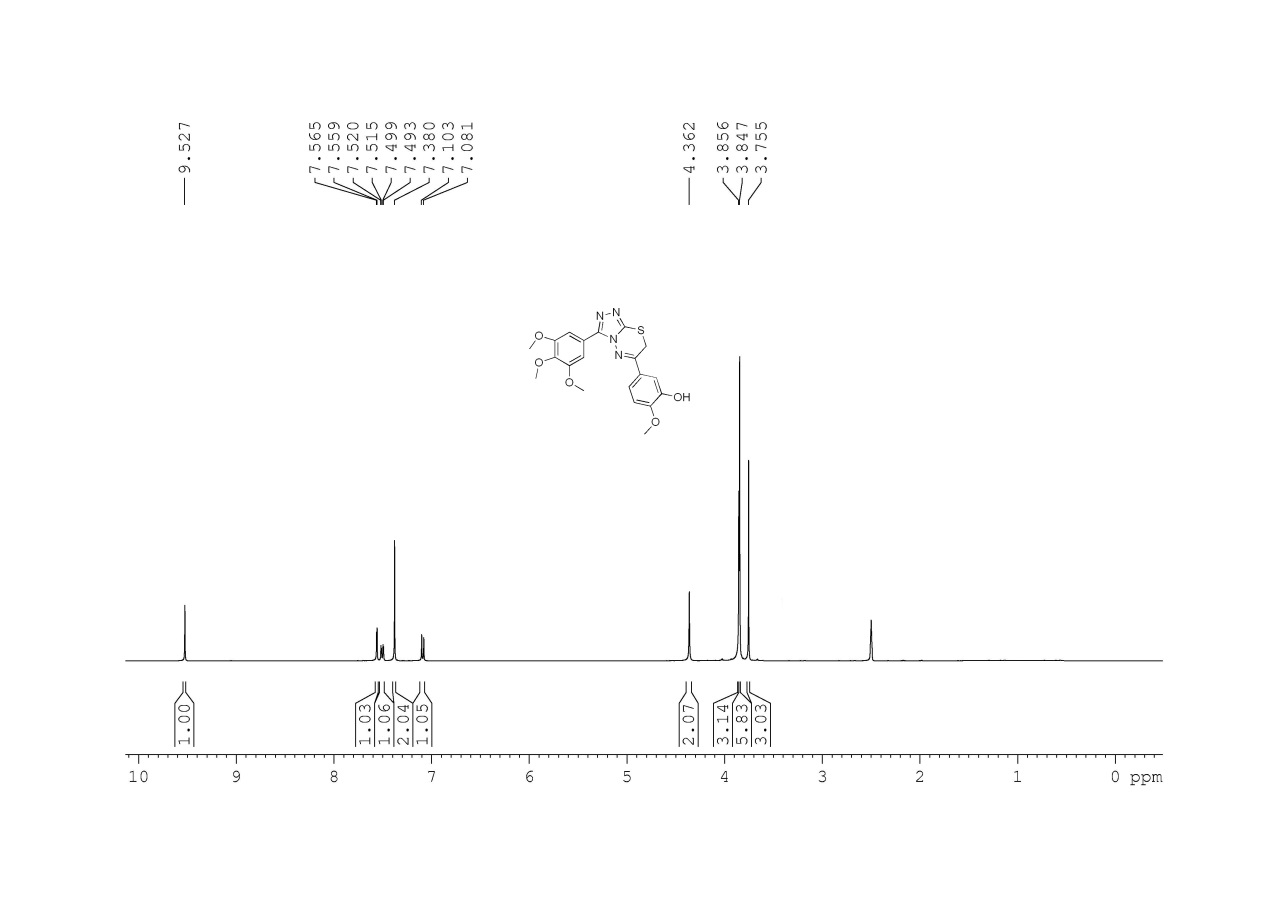

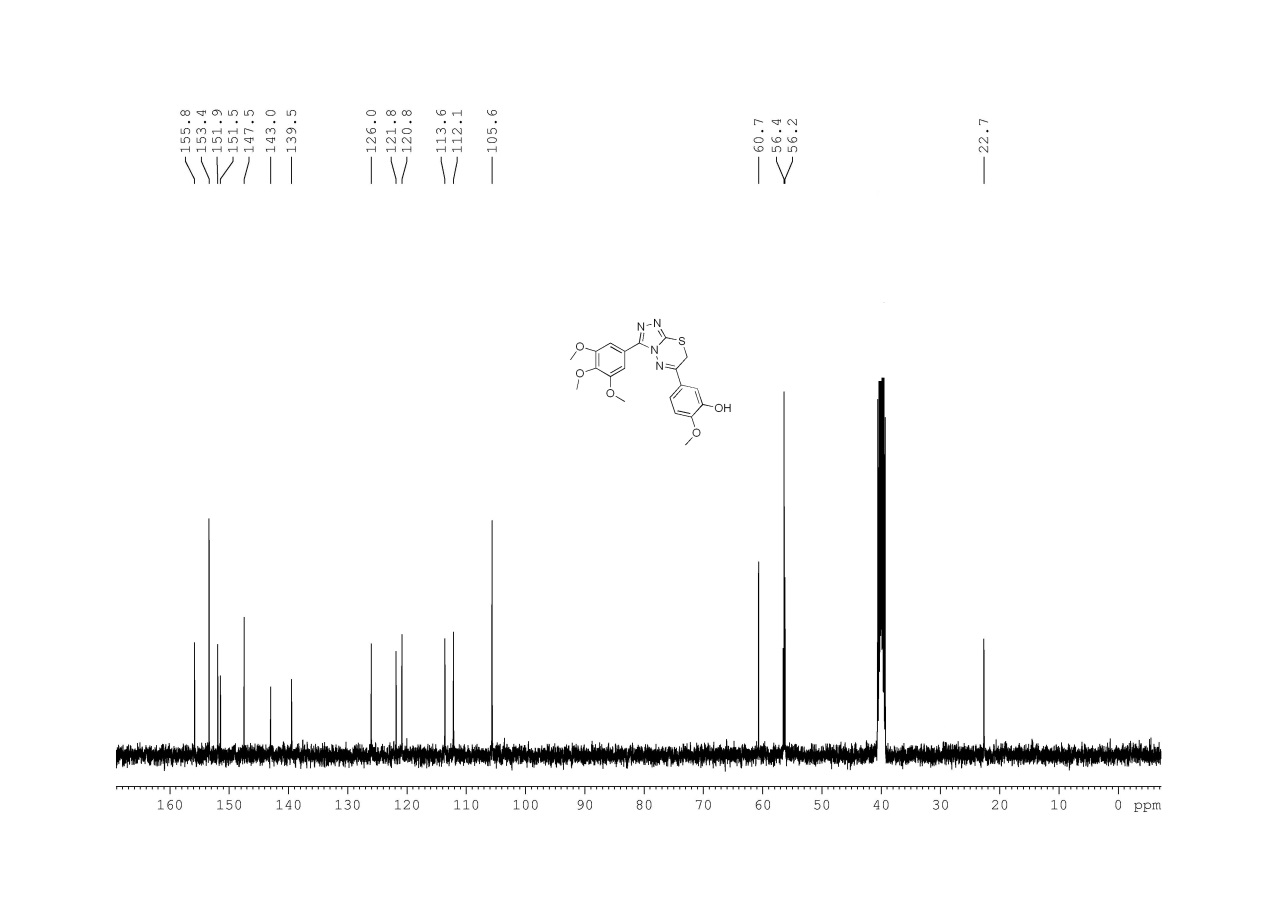


3-(3,4-Methylenedioxyphenyl)-6-(3-fluoro-4-methoxyphenyl)-*7H*-[1,2,4]triazolo[3,4-b][1,3,4]thiadiazine (**6a**).


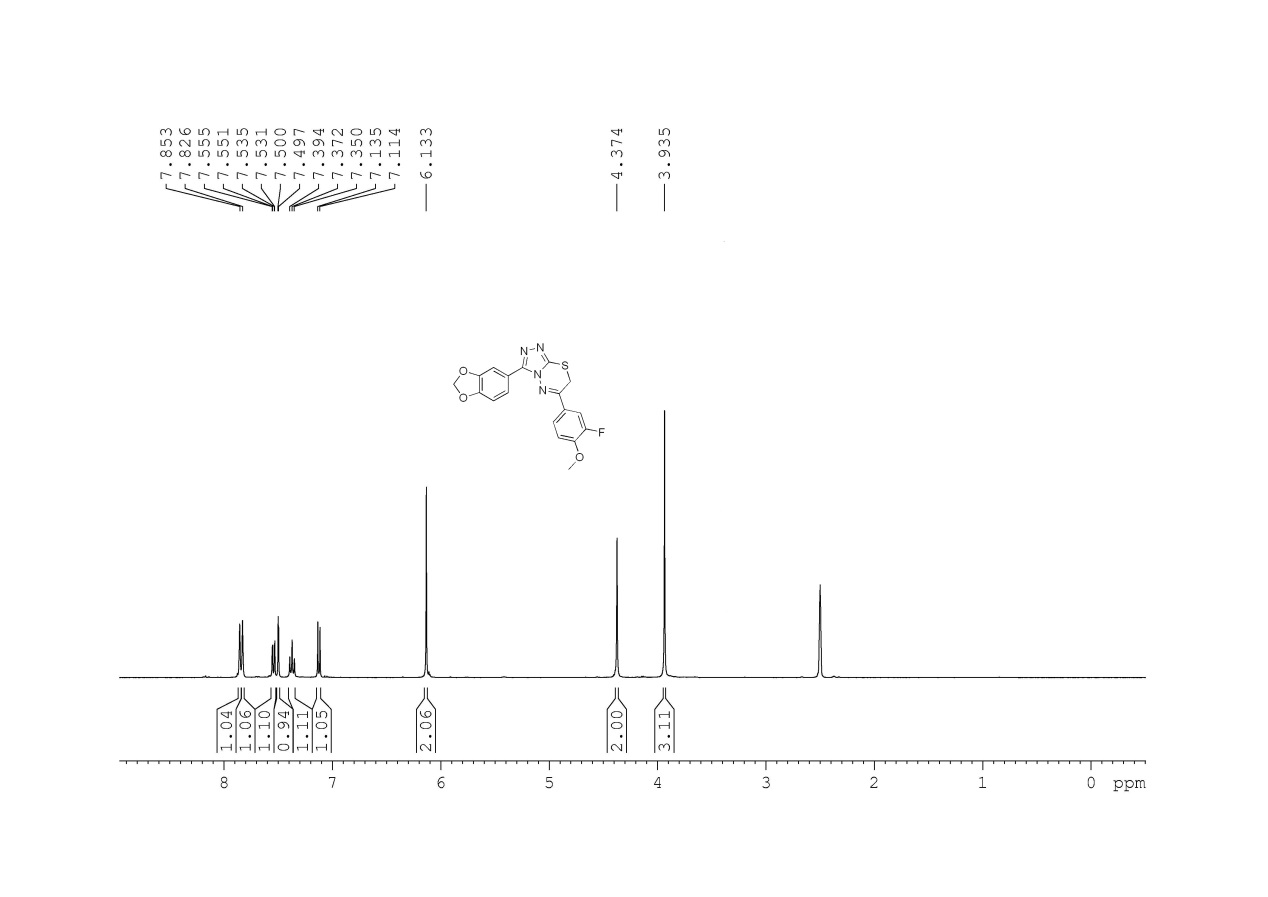

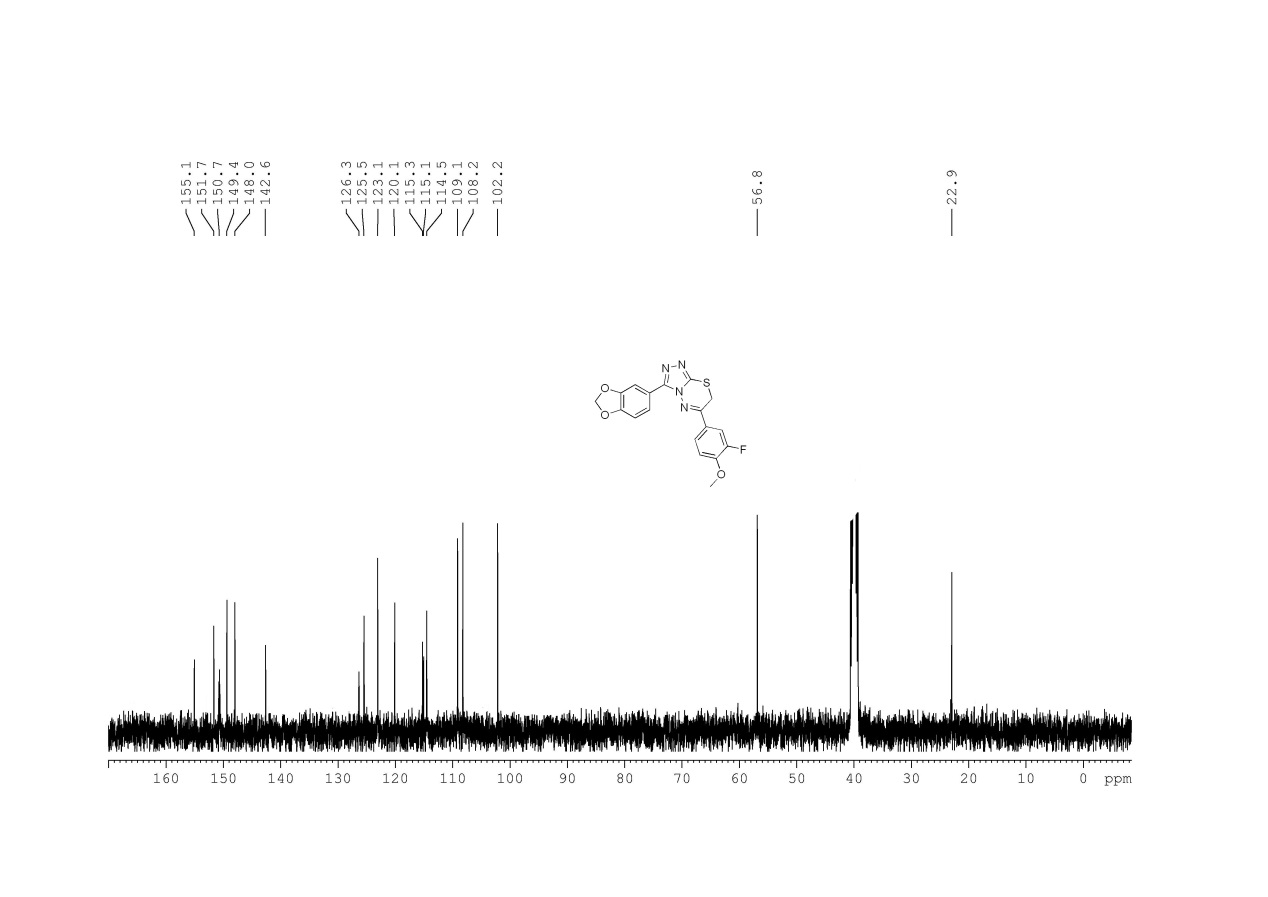


3-(3,4-Methylenedioxyphenyl)-6-(3-nitro-4-methoxyphenyl)-*7H*-[1,2,4]triazolo[3,4-b][1,3,4]thiadiazine (**6b**).


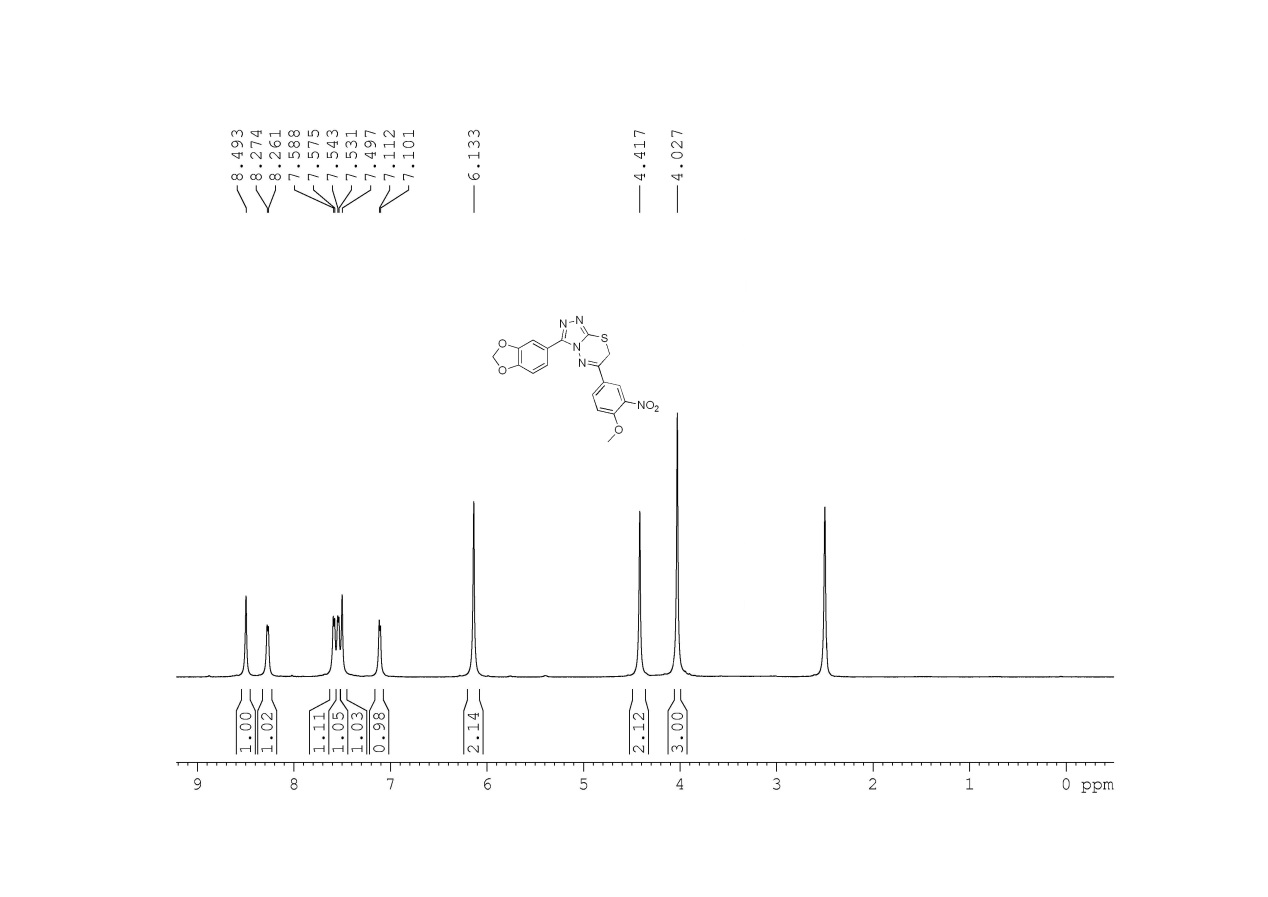

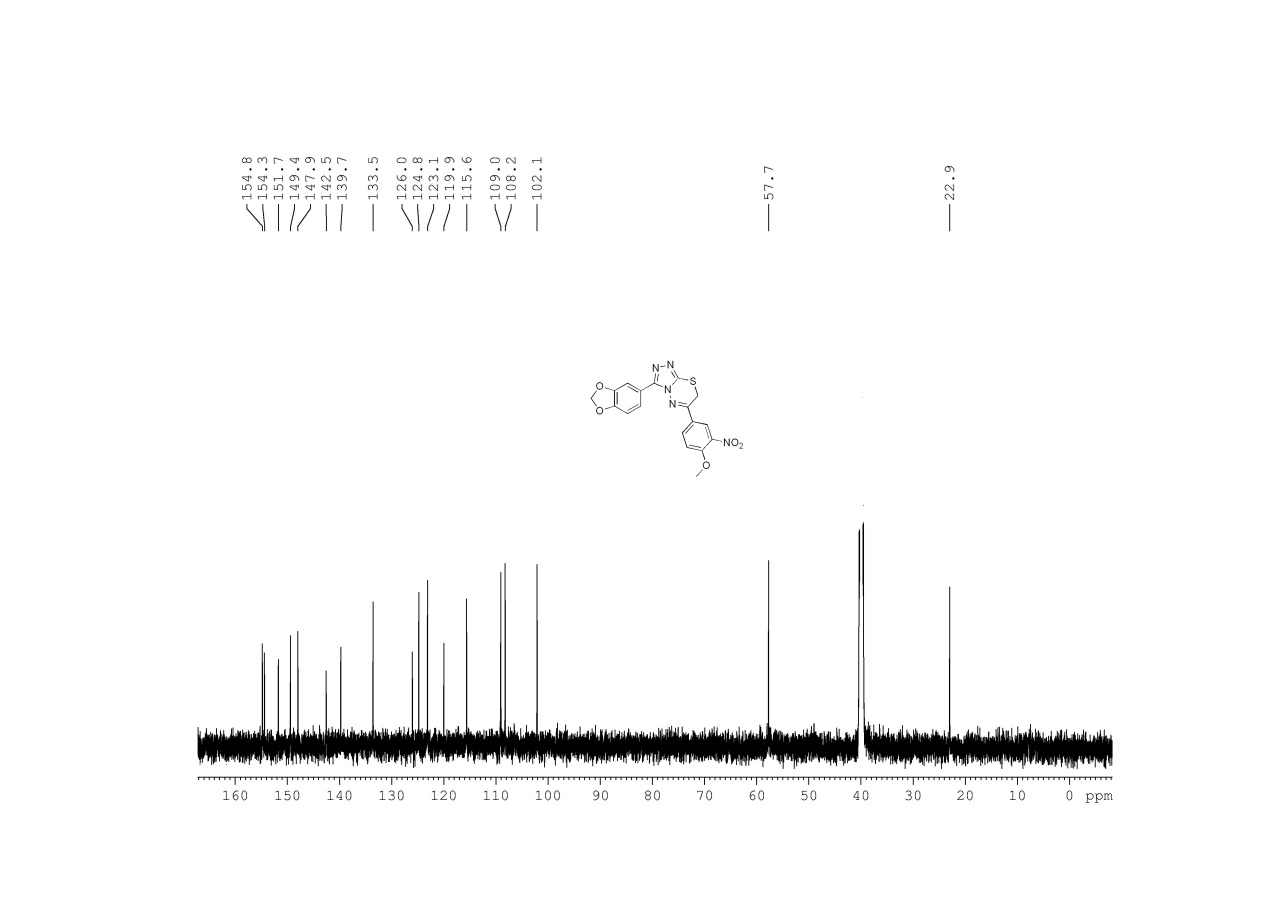


3-(3,4-Methylenedioxyphenyl)-6-(3-amino-4-methoxyphenyl)-*7H*-[1,2,4]triazolo[3,4-b][1,3,4]thiadiazine (**6c**).


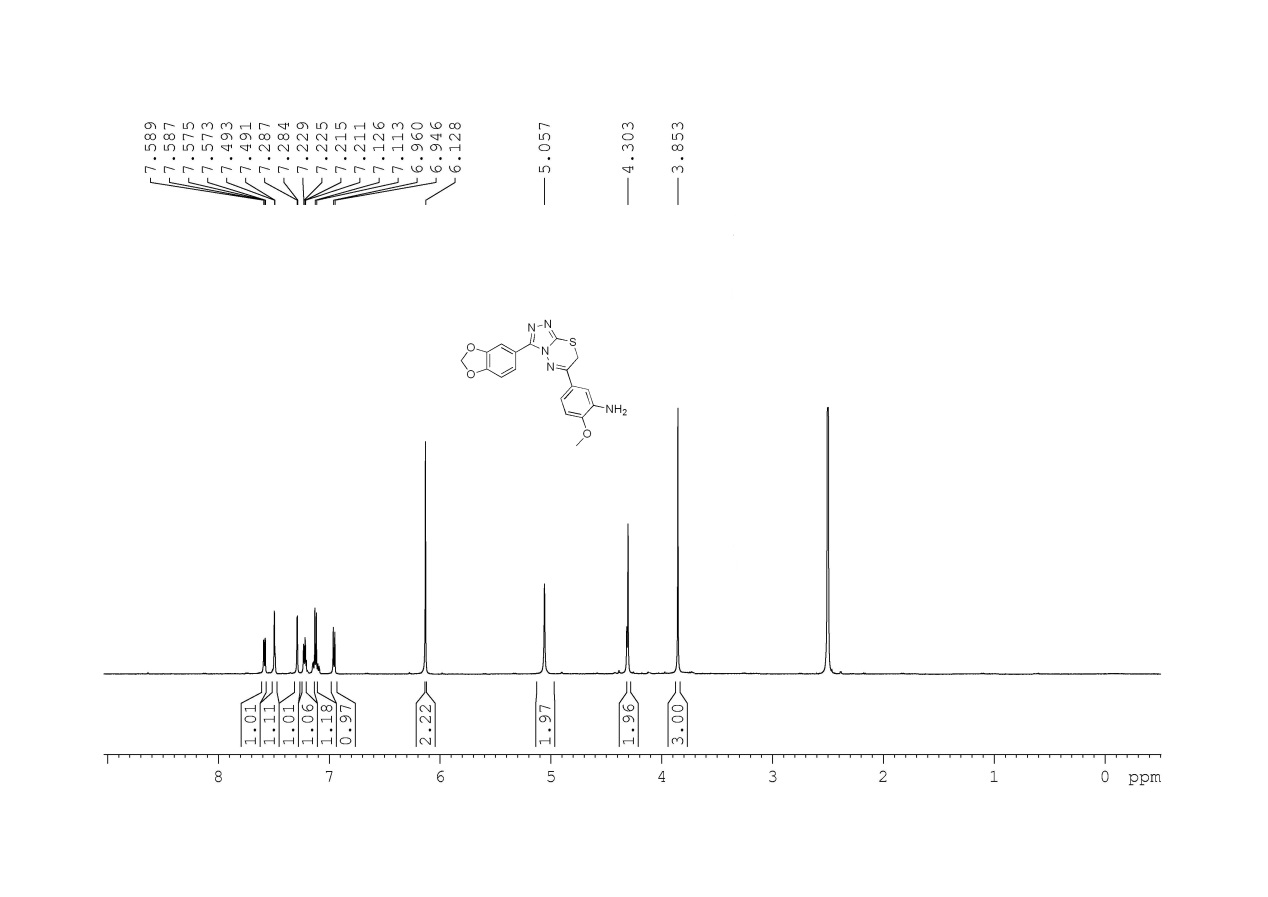

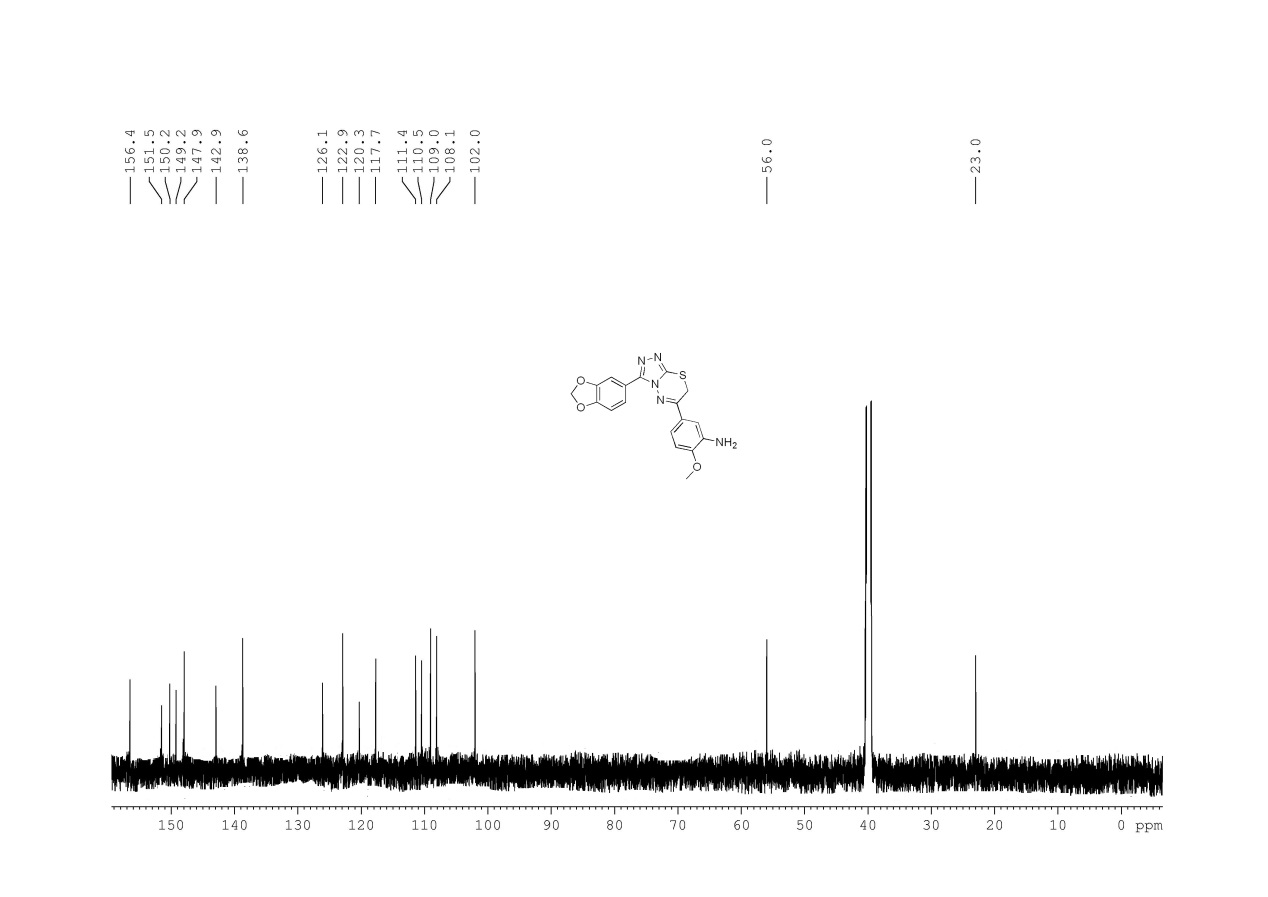


3-(3,4-Methylenedioxyphenyl)-6-(3-benzyloxy-4-methoxyphenyl)-*7H*-[1,2,4]triazolo[3,4-b][1,3,4]thiadiazine (**6d**).


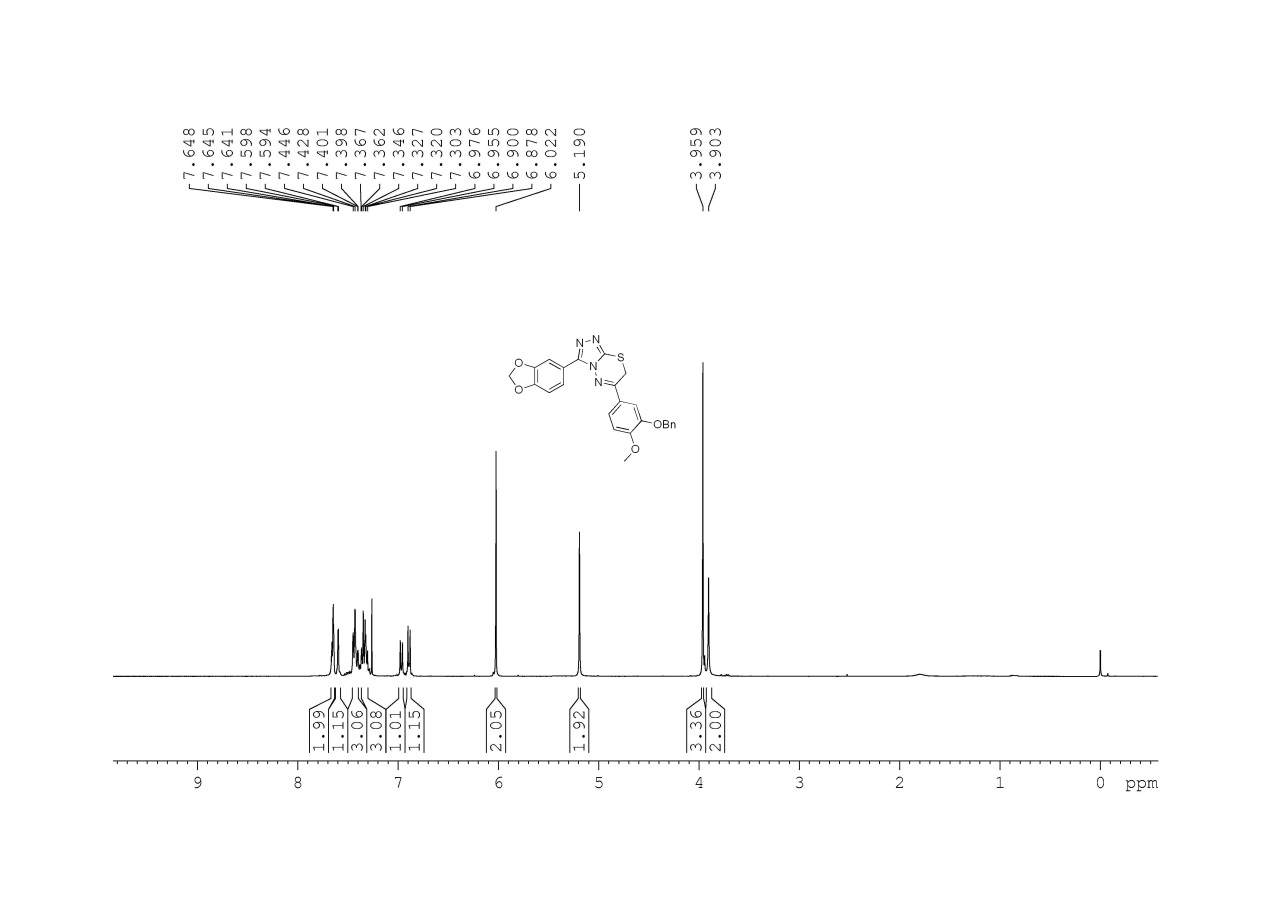

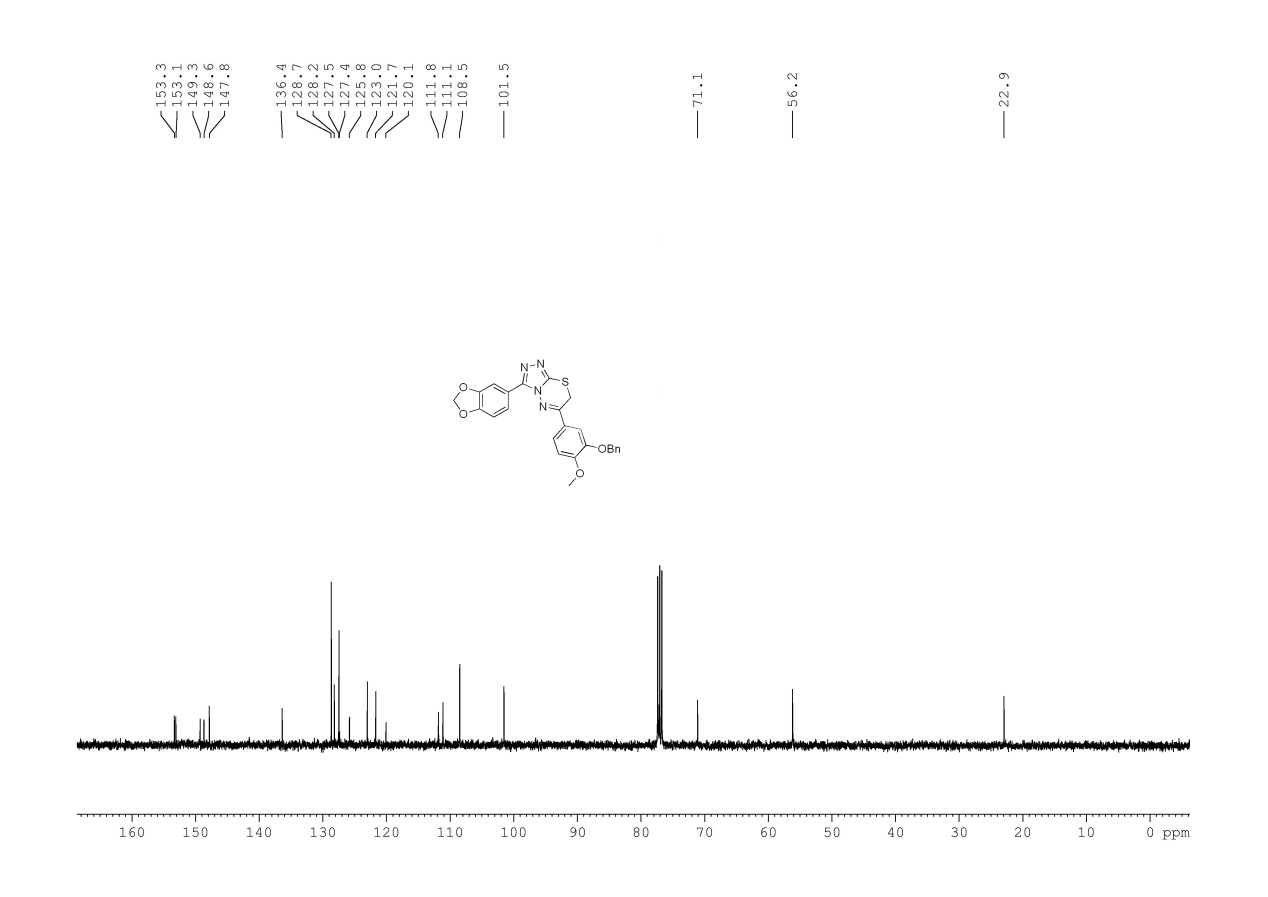


3-(3,4-Methylenedioxyphenyl)-6-(3-hydroxy-4-methoxyphenyl)-*7H*-[1,2,4]triazolo[3,4-b][1,3,4]thiadiazine (**6e**).


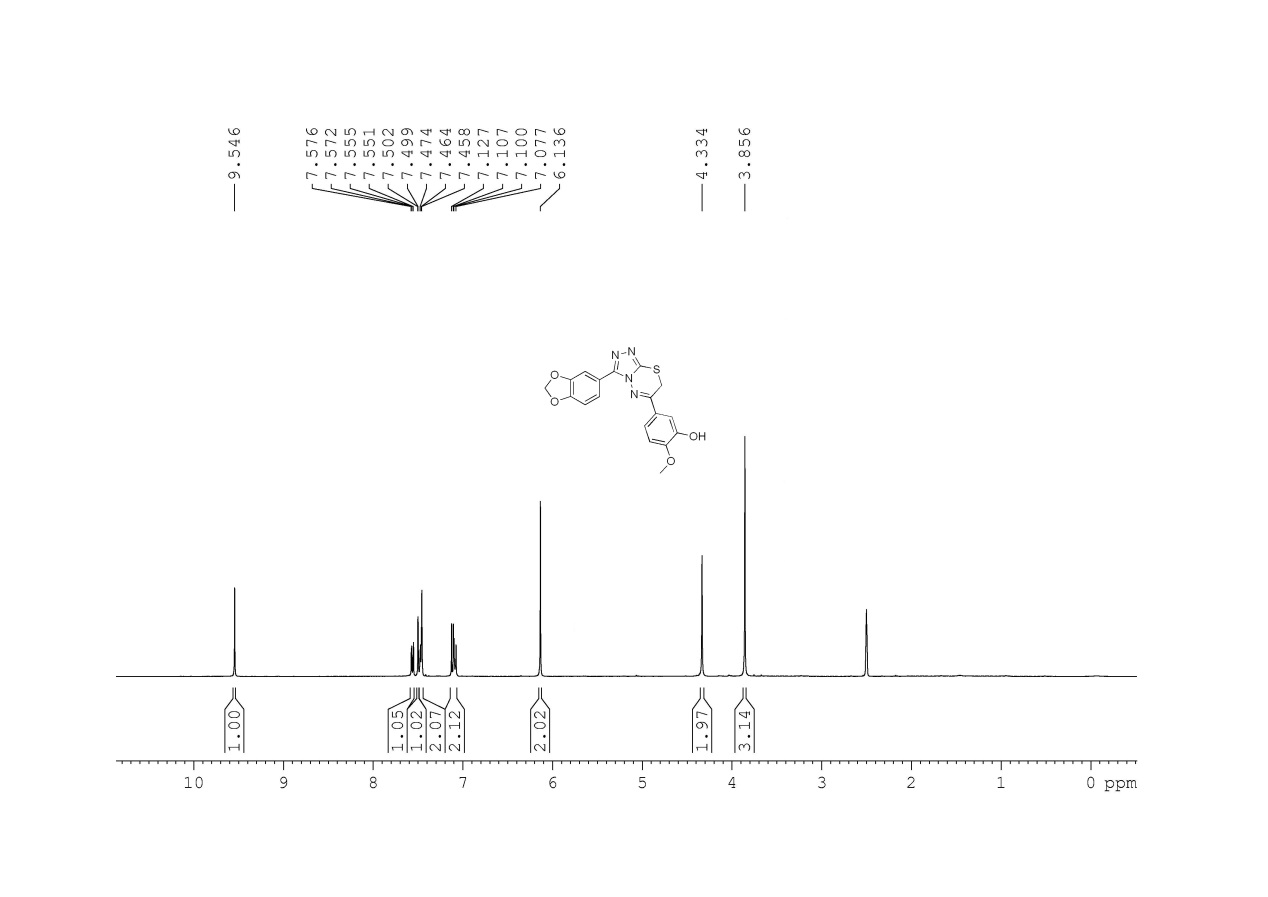

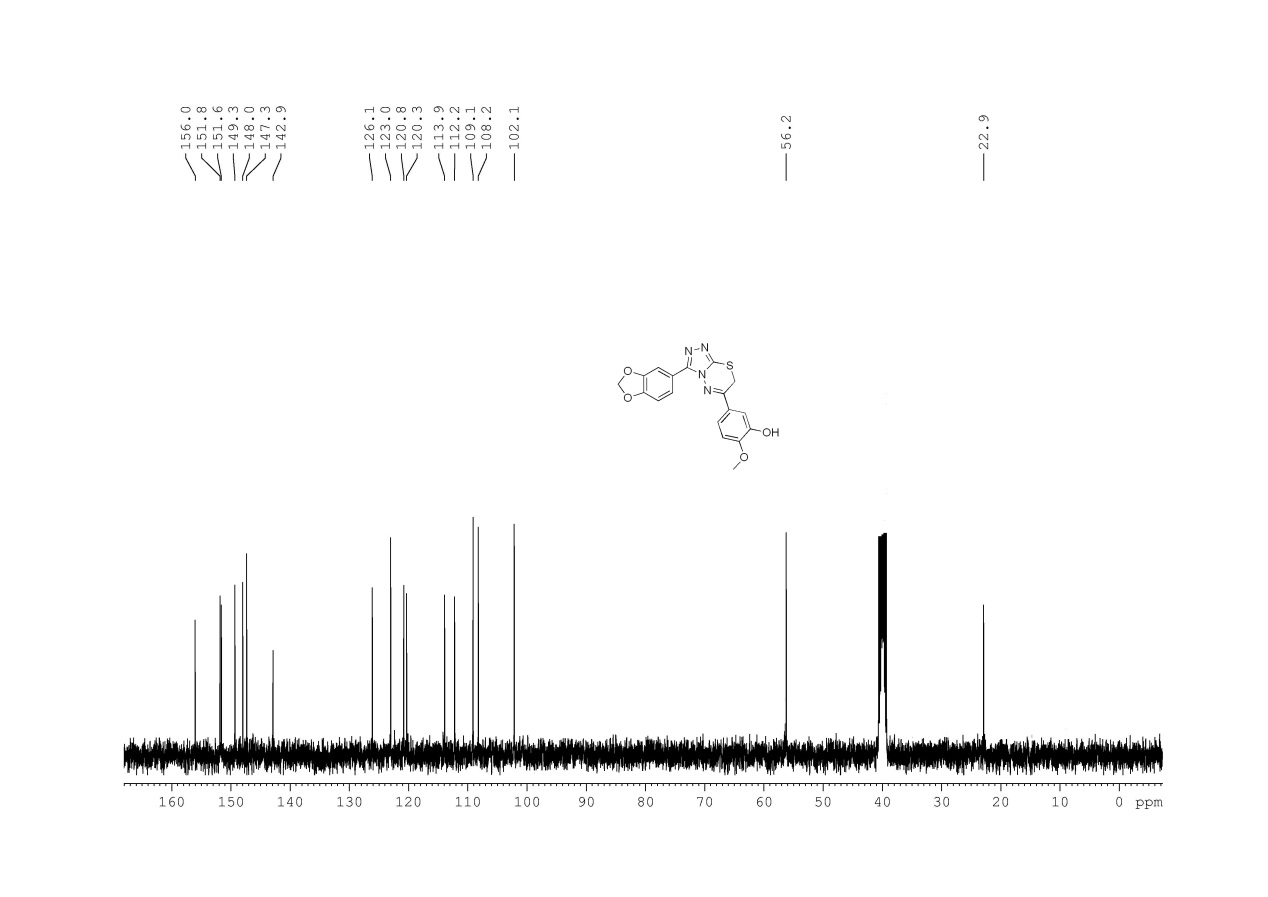


3-(3,4-Dimethoxyphenyl)-6-(3-nitro-4-methoxyphenyl)-*7H*-[1,2,4]triazolo[3,4-b][1,3,4]thiadiazine (**6f**).


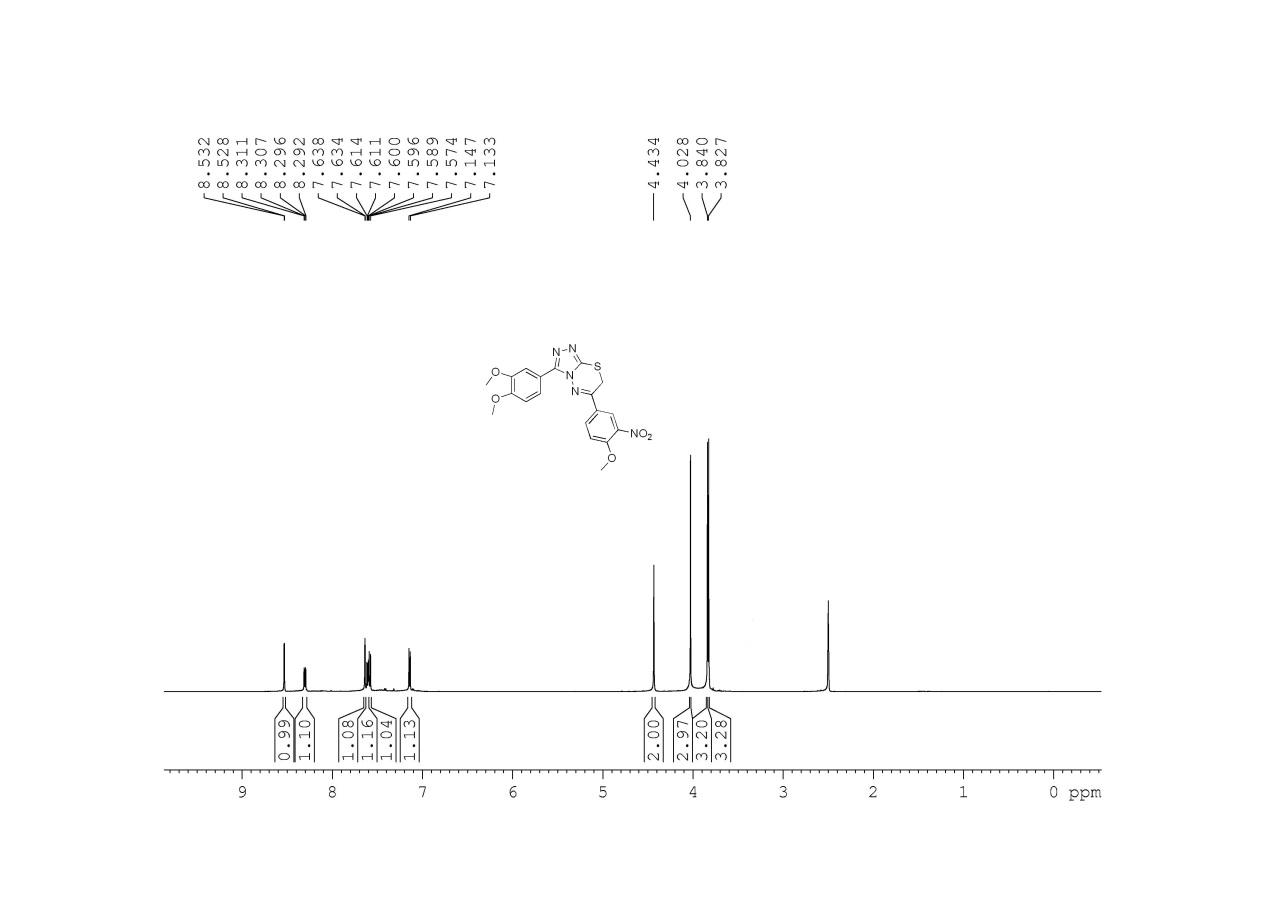

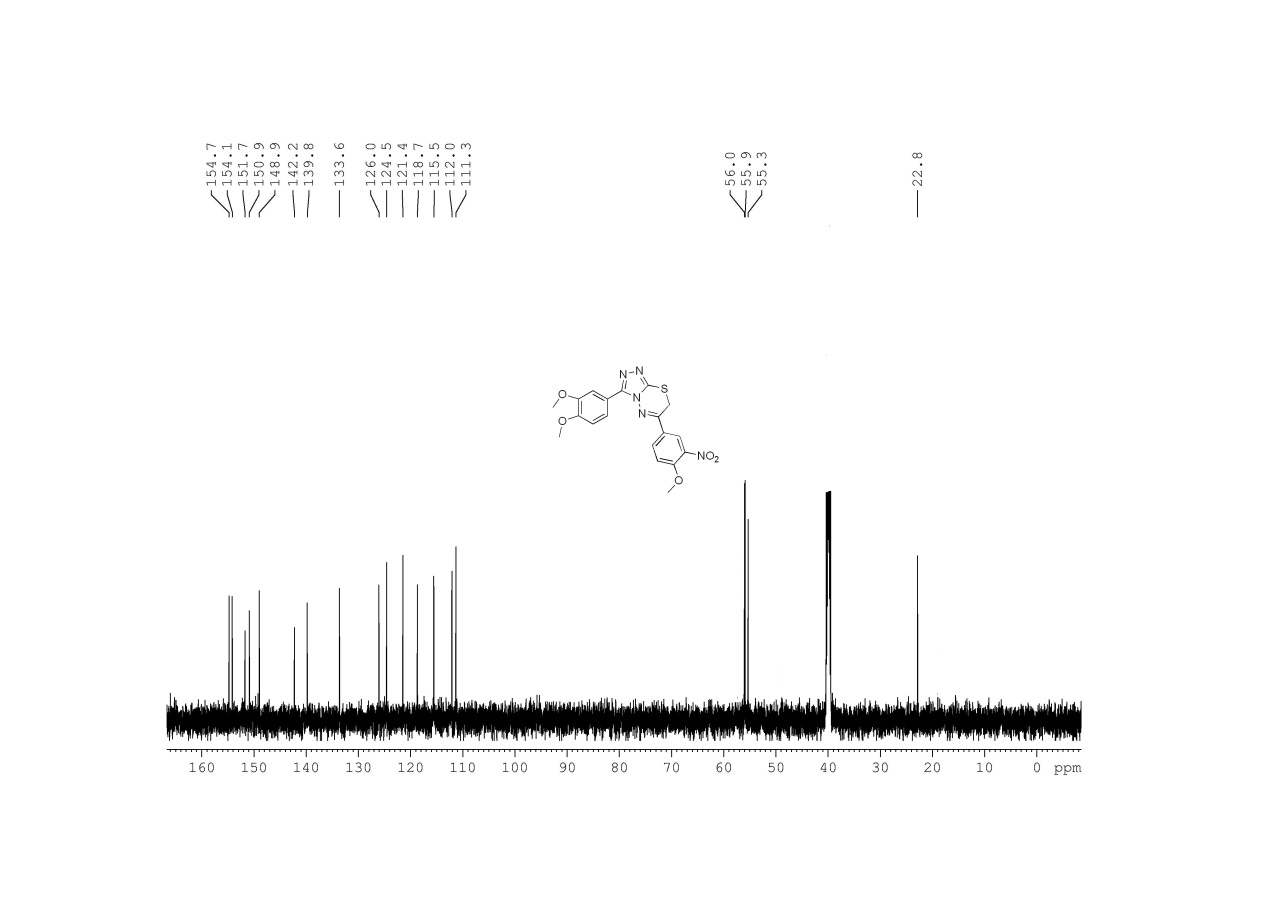


3-(3,4-Dimethoxyphenyl)-6-(3-amino-4-methoxyphenyl)-*7H*-[1,2,4]triazolo[3,4-b][1,3,4]thiadiazine (**6g**).


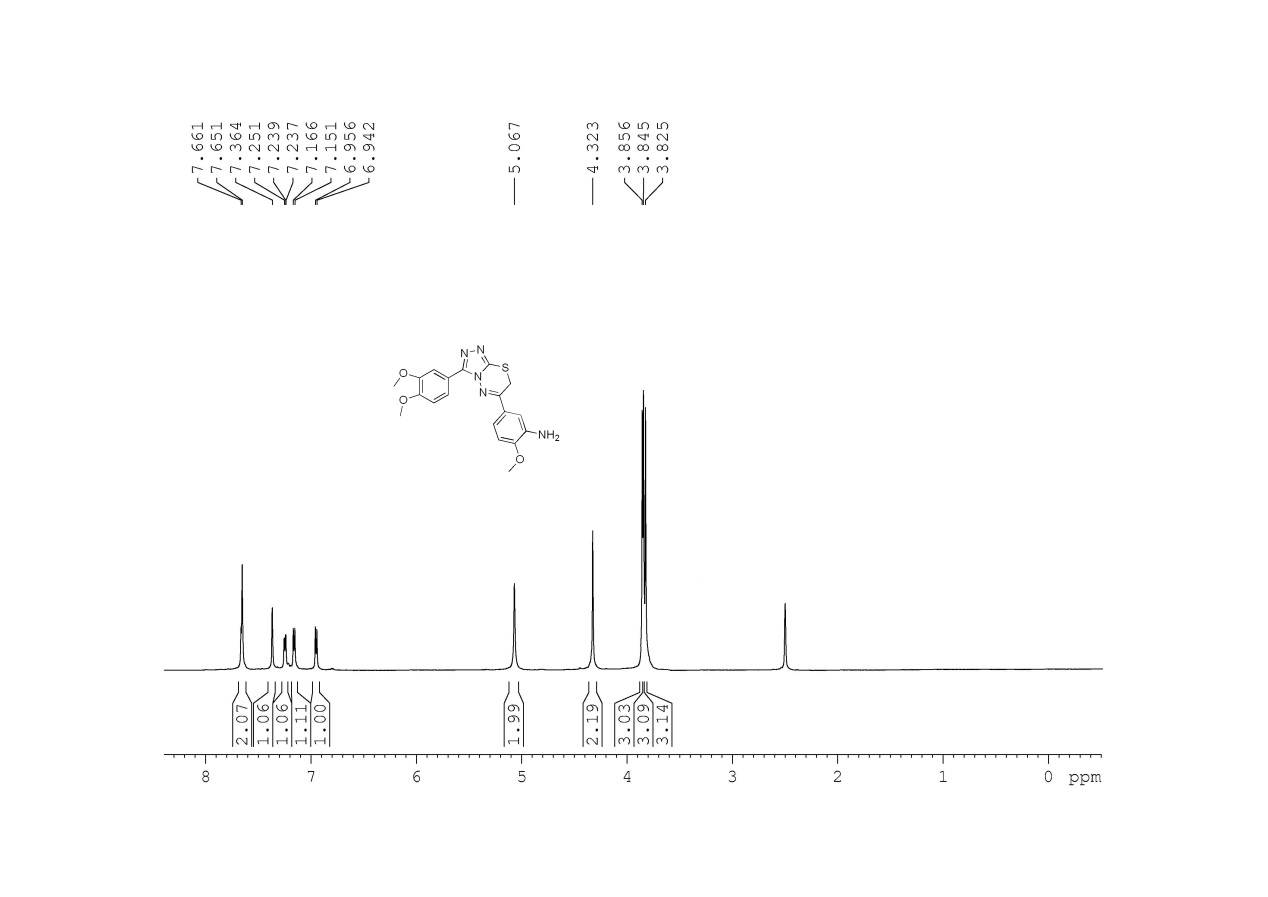

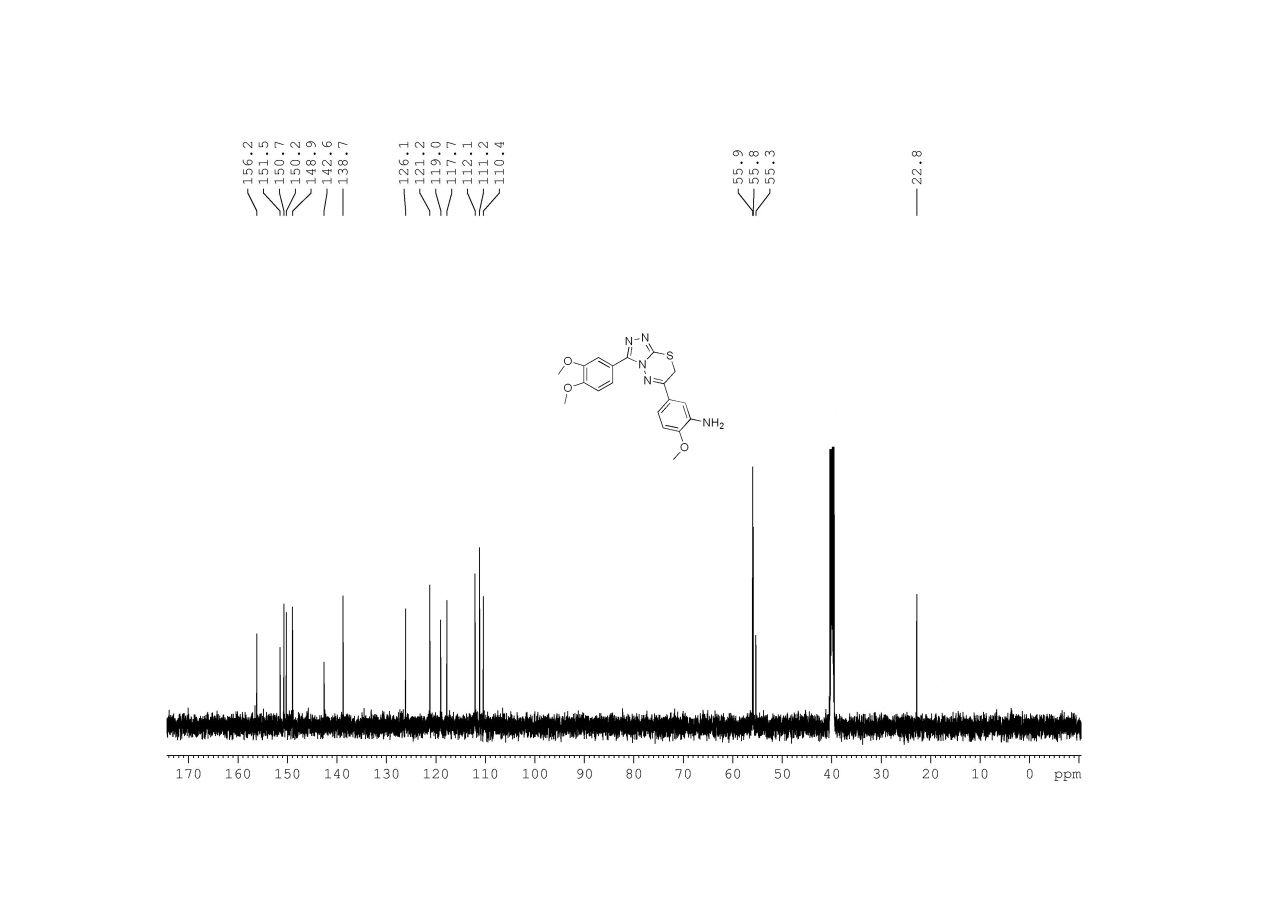


3-(3-Methoxyphenyl)-6-(3-nitro-4-methoxyphenyl)-*7H*-[1,2,4]triazolo[3,4-b][1,3,4]thiadiazine (**6h**).


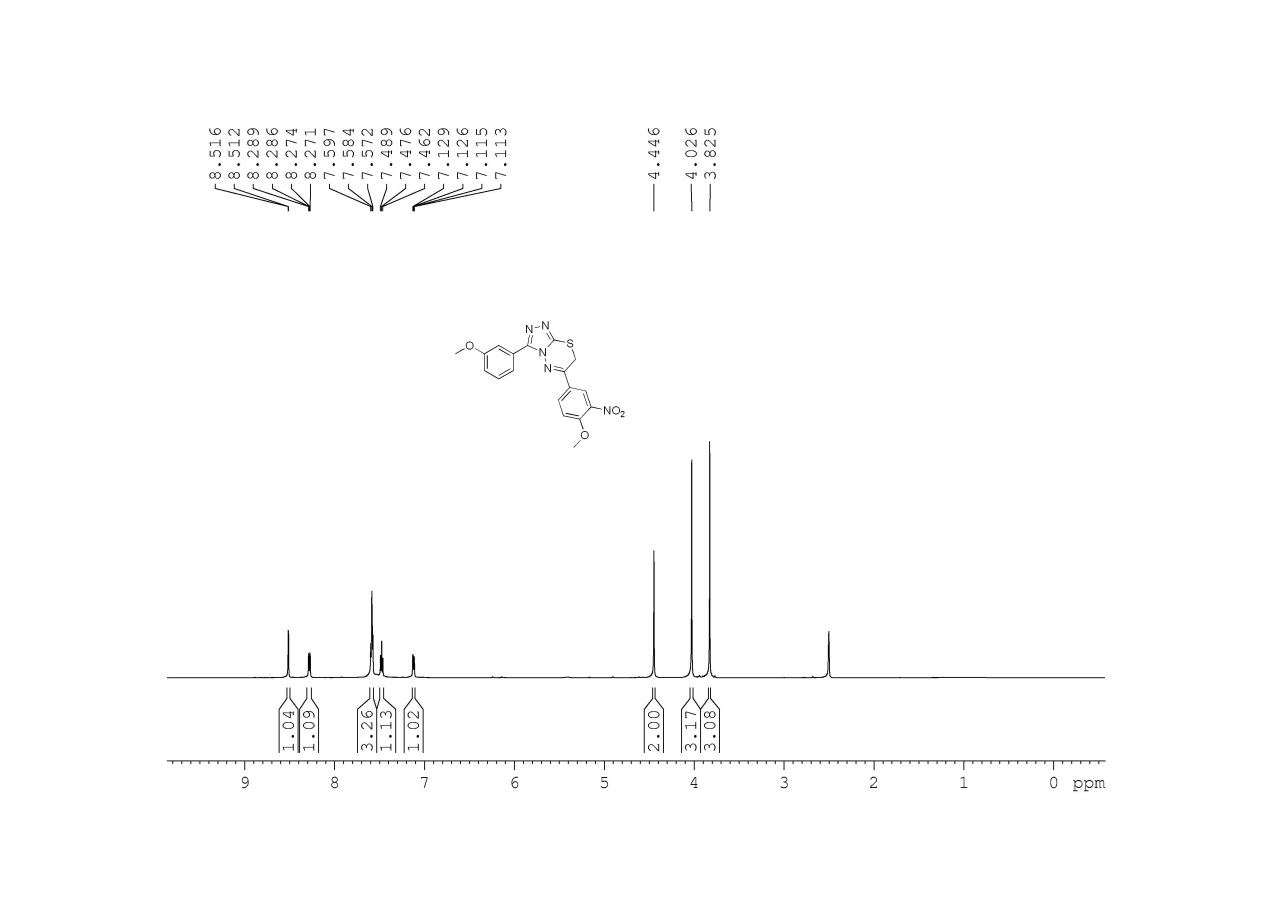


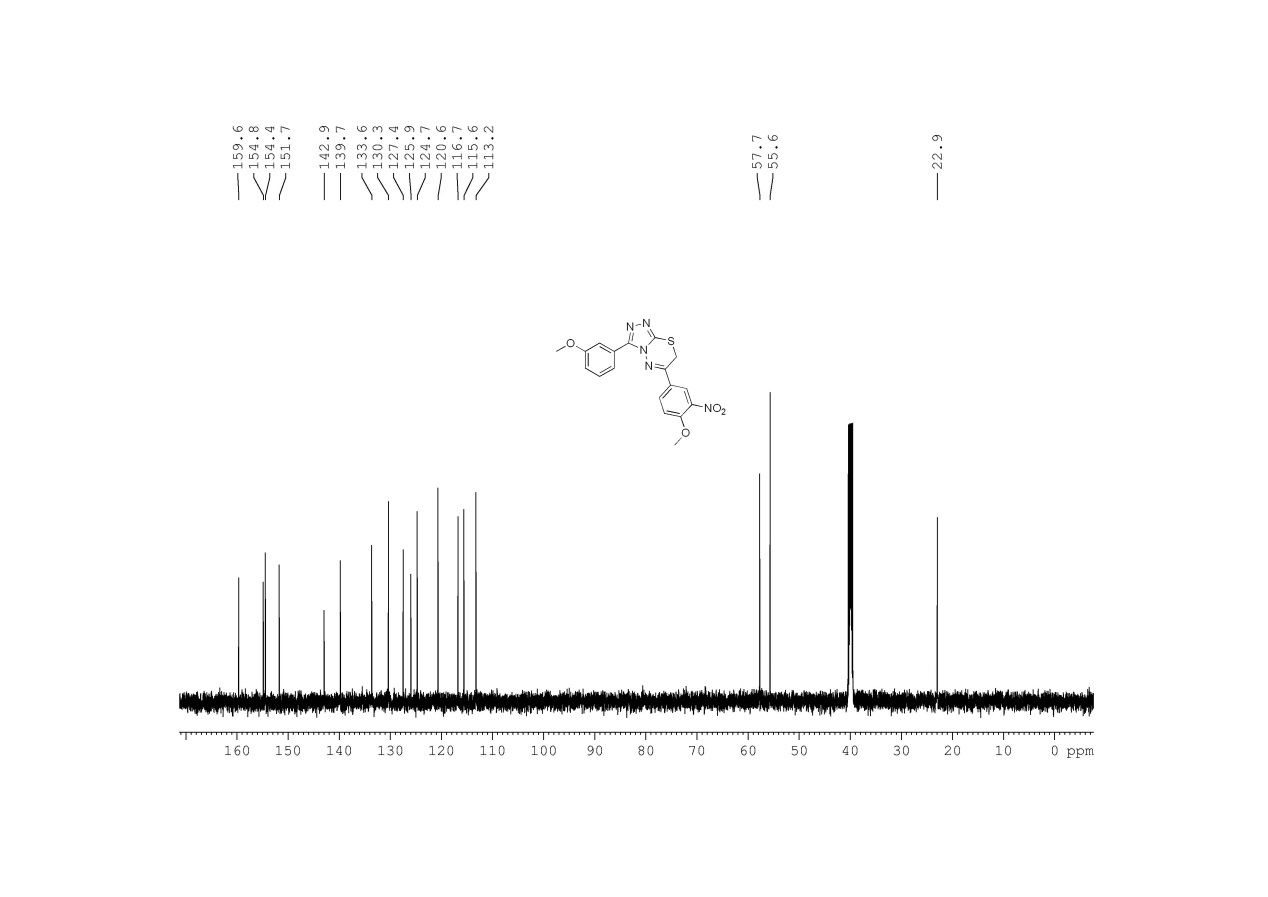


3-(3-Methoxyphenyl)-6-(3-amino-4-methoxyphenyl)-*7H*-[1,2,4]triazolo[3,4-b][1,3,4]thiadiazine (**6i**).


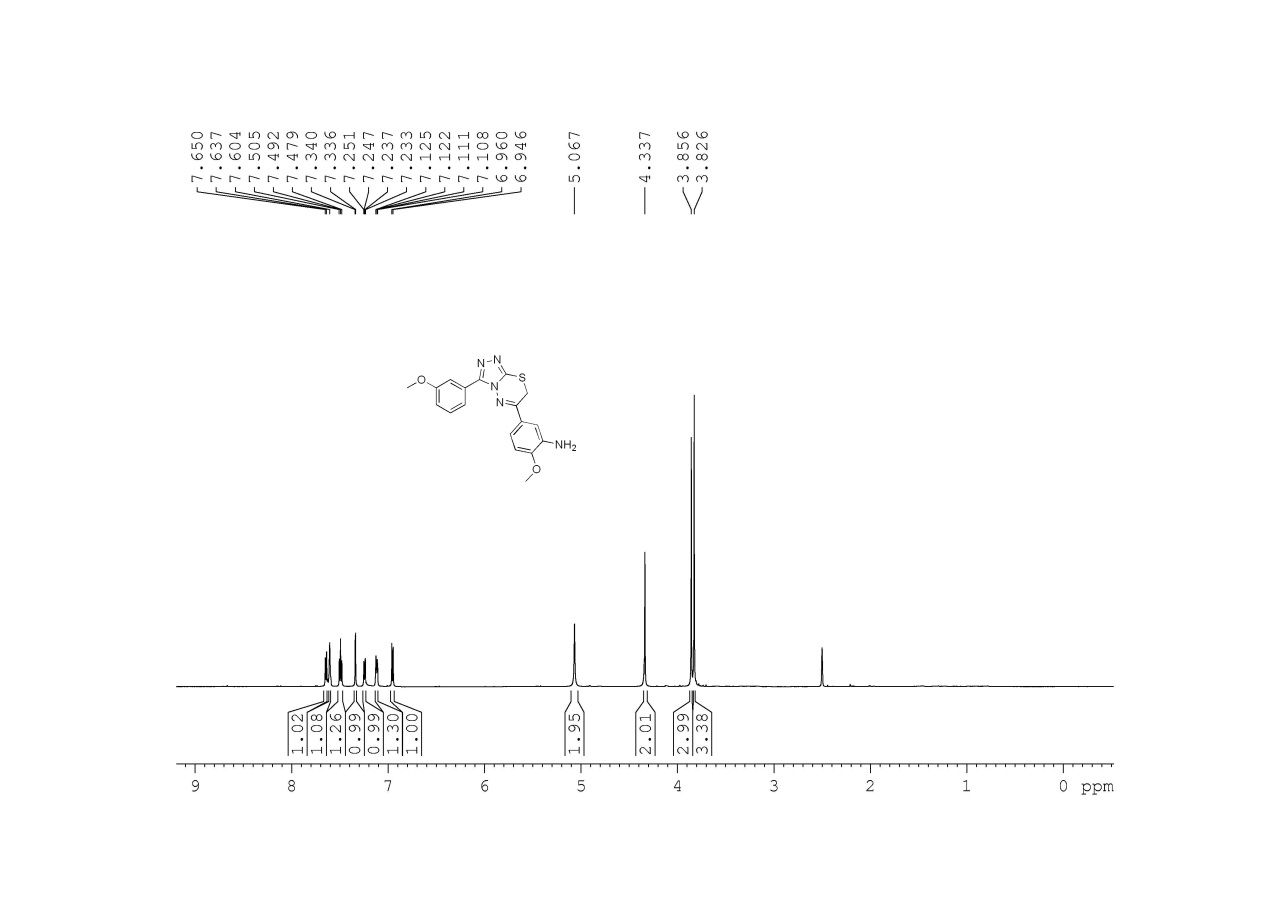

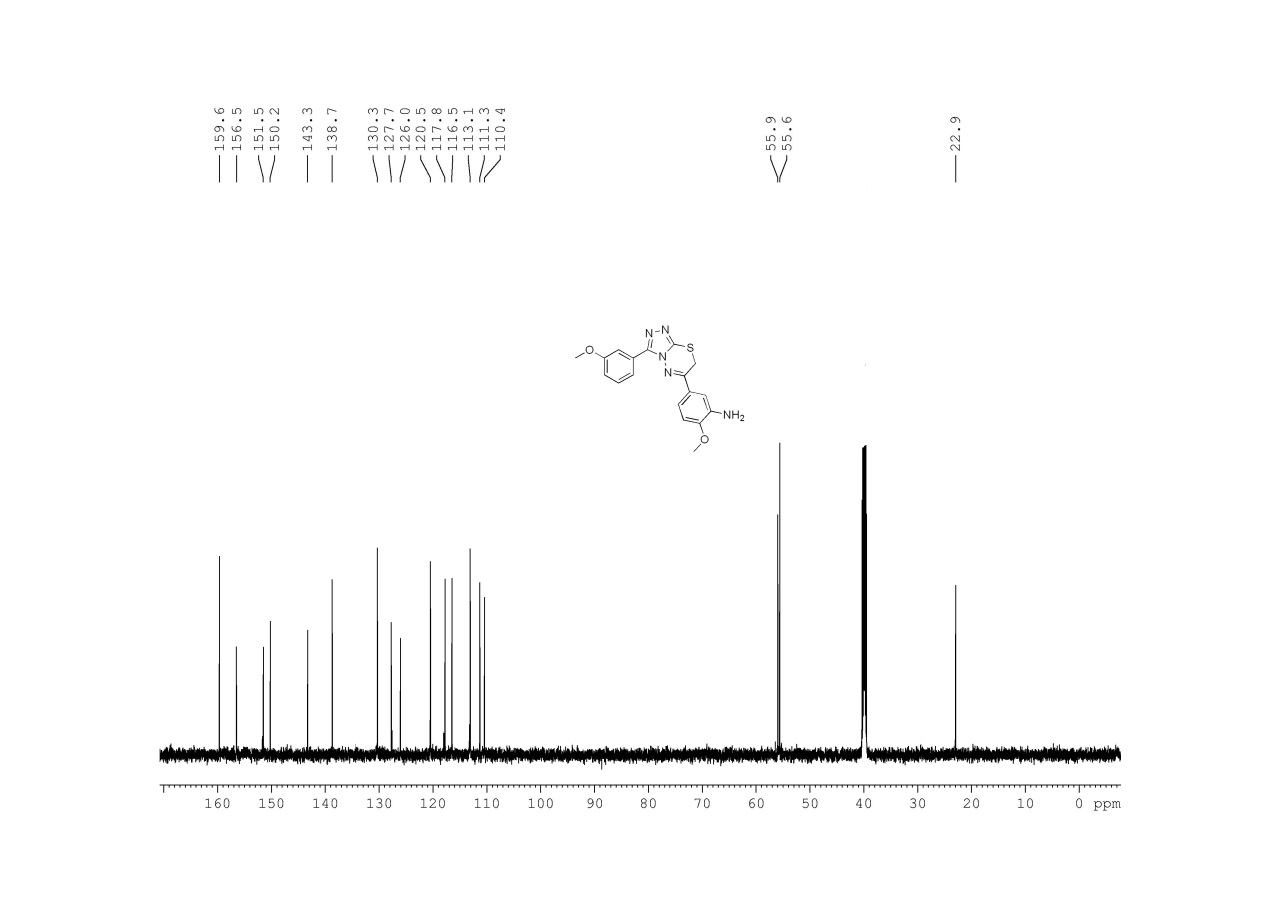


3-(4-Methoxyphenyl)-6-(3-nitro-4-methoxyphenyl)-*7H*-[1,2,4]triazolo[3,4-b][1,3,4]thiadiazine (**6j**).


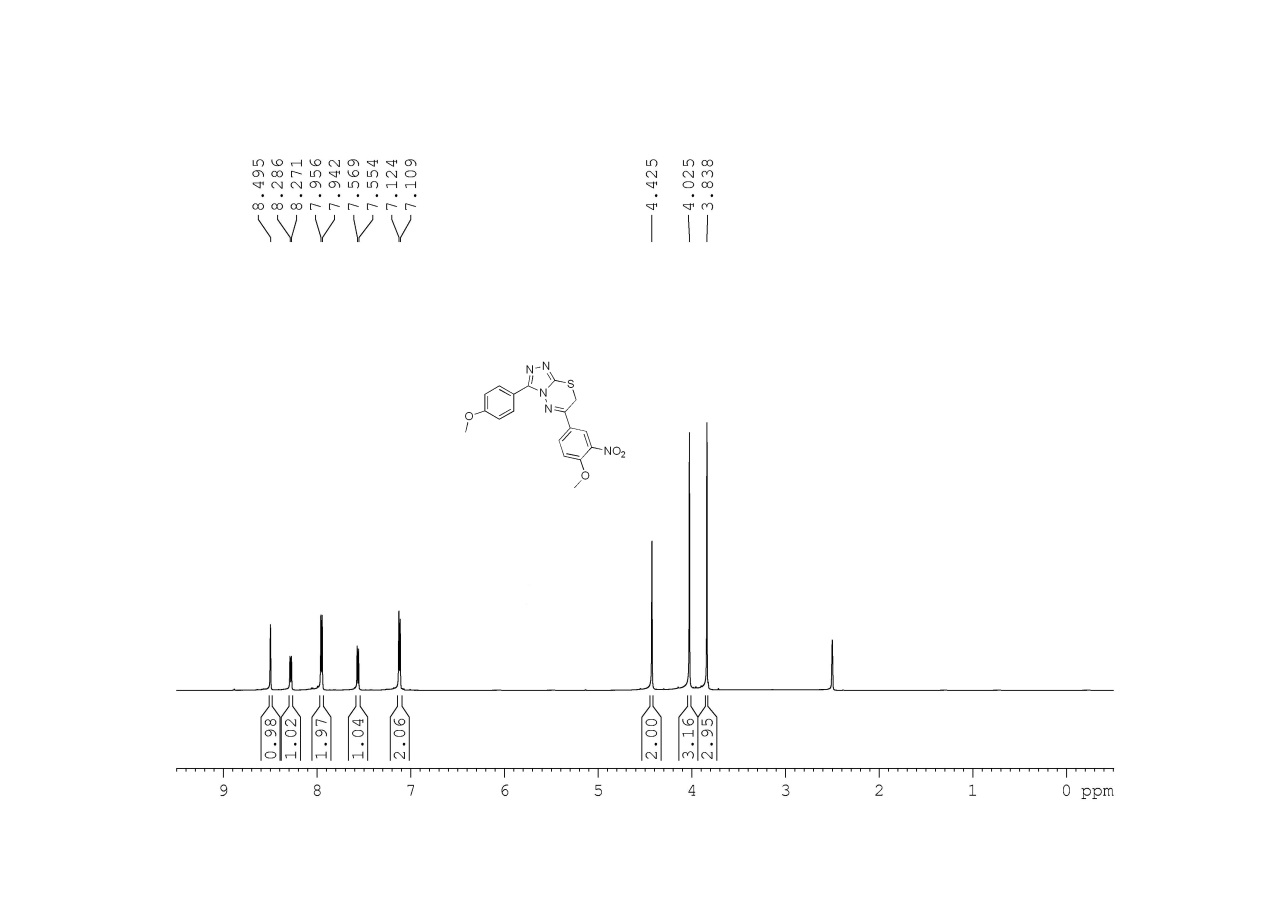

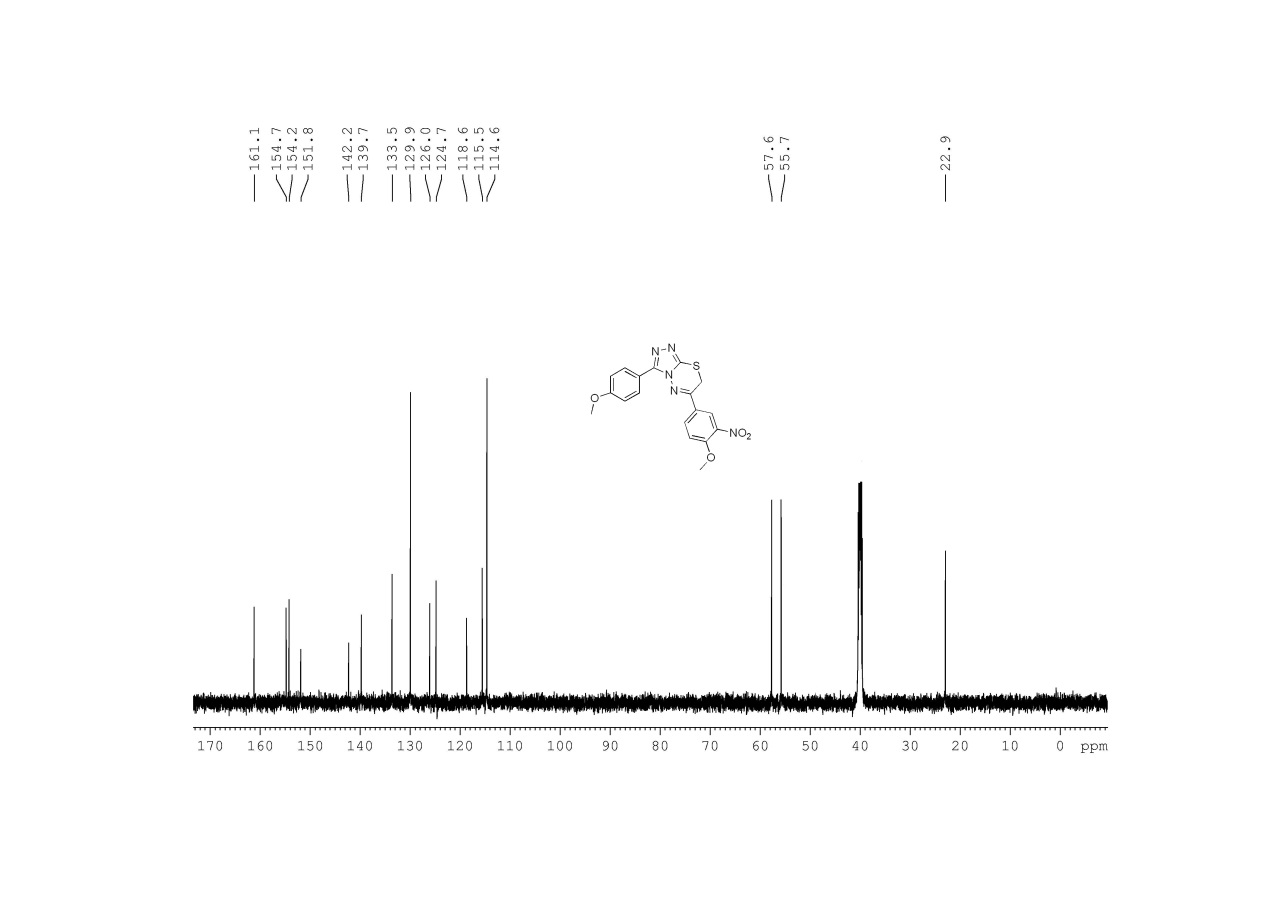


3-(4-Methoxyphenyl)-6-(3-amino-4-methoxyphenyl)-*7H*-[1,2,4]triazolo[3,4-b][1,3,4]thiadiazine (**6k**).


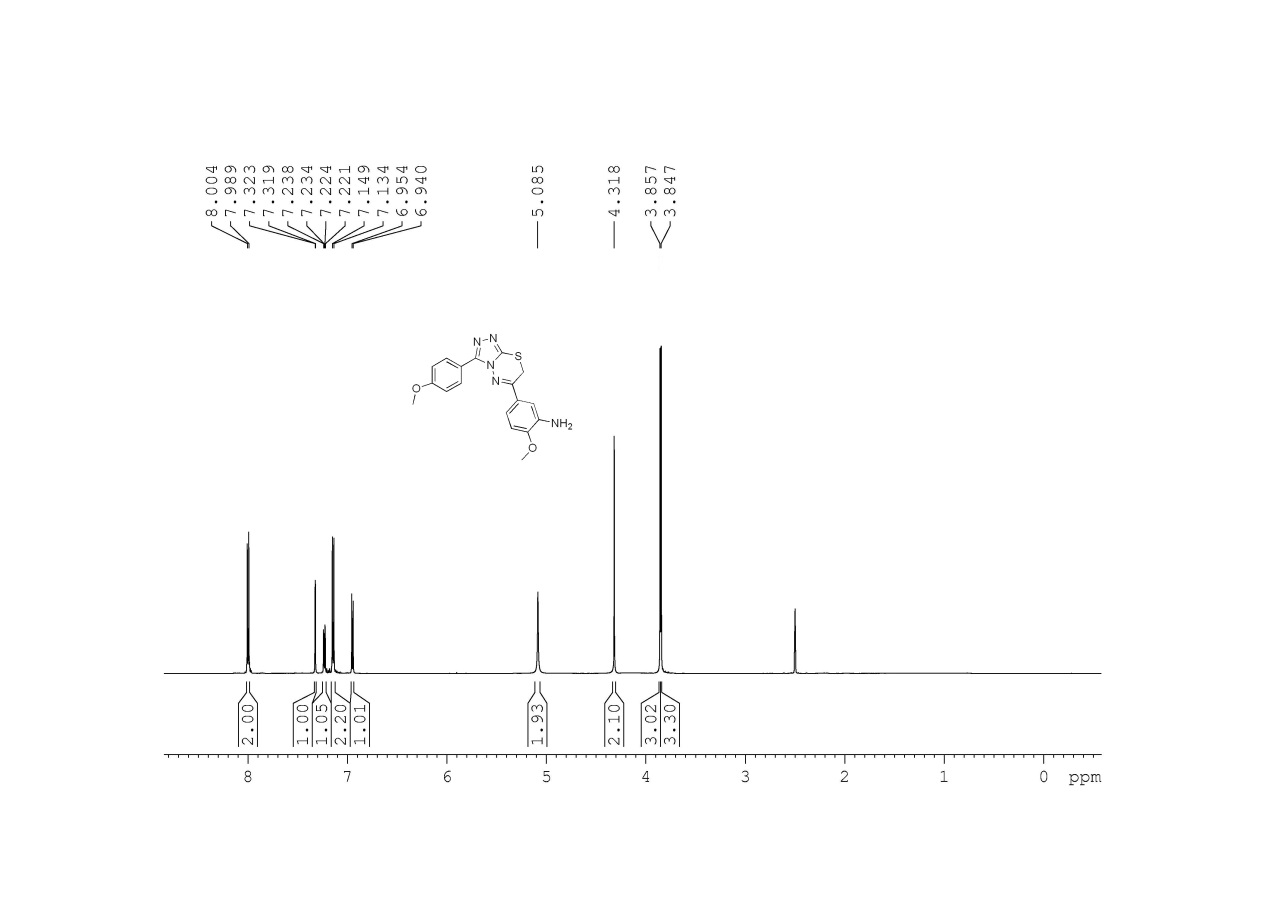

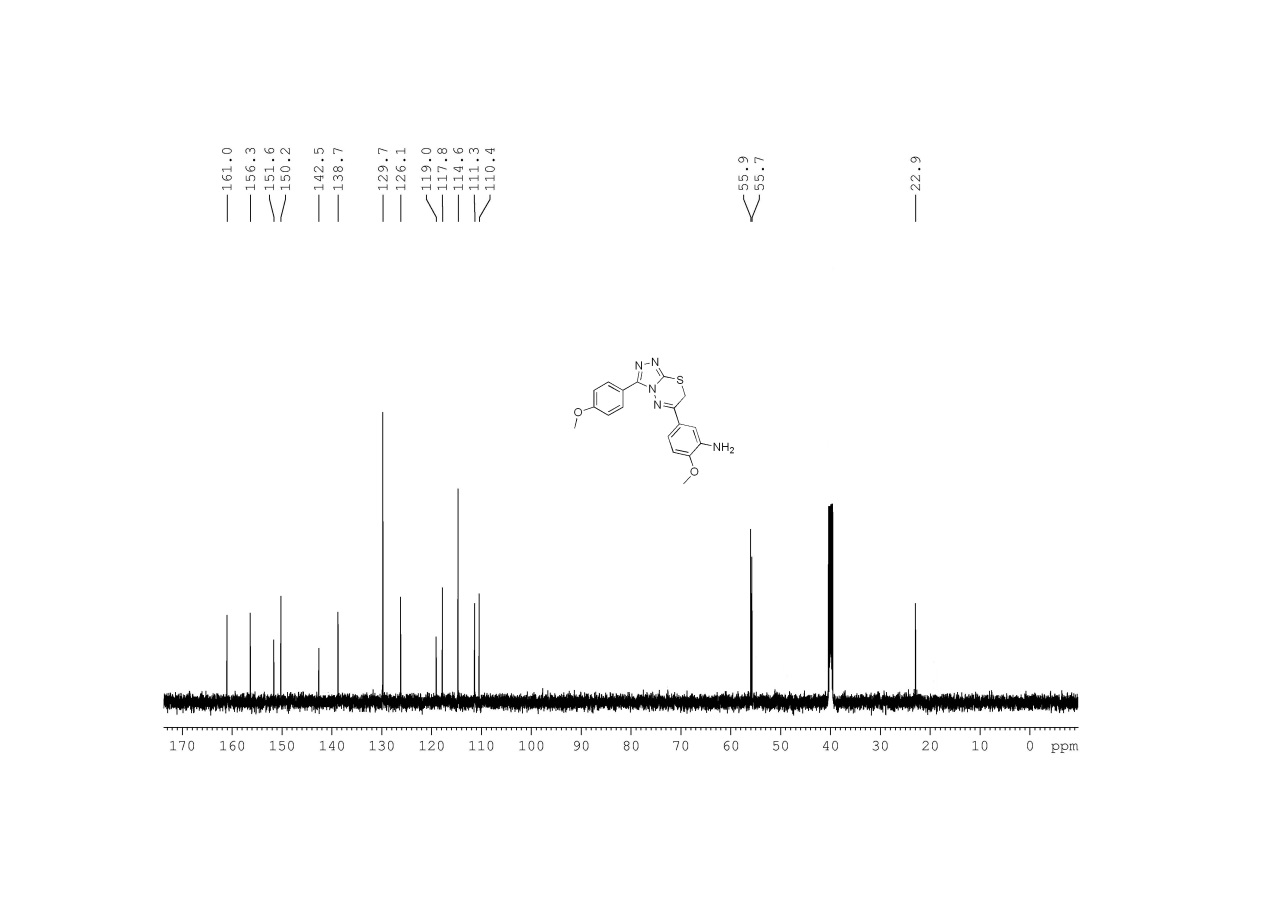

Supplement: Supplementary file 1 — Supplementary information [file 41598_2017_10860_MOESM1_ESM.doc]
